# Direct metathesis of carbon–carbon *σ*-bonds at a versatile macrocycle-supported diiron platform

**DOI:** 10.1039/d5sc10054b

**Published:** 2026-02-06

**Authors:** Tianchang Liu, Jared E. Gonder, Ryan P. Murphy, Alexandra M. Bacon, Michael R. Gau, Neil C. Tomson

**Affiliations:** a Department of Chemistry, University of Pennsylvania 231 S 34th St Philadelphia Pennsylvania 19104 USA tomson@upenn.edu

## Abstract

The metathesis of C–C *σ*-bonds holds potential as an atom-economical strategy for molecular engineering. While this reaction has been achieved in industrial and research settings, the former is energy-intensive and the latter has only been reported to operate either indirectly, proceeding through well-known *π*-bond metathesis chemistry, or through the use of structural/electronic factors that assist in C–C bond activation (*e.g.* anchimeric effects, directing groups, photochemical effects, *etc.*). Herein, we report the first example of a direct, thermodynamically favored C–C *σ*-bond metathesis reaction in which the C(sp)–C(sp^2^) bonds of diarylacetylenes are selectively cleaved before their fragments are metathesized and C(sp)–C(sp^2^) linkages are re-formed. Building on a known macrocycle-supported diiron system that undergoes selective C–C *σ*-bond oxidative addition, Lewis acid catalysts are shown to reversibly abstract Fe-bound aryl groups and perform aryl group exchange between diiron sites. Oxidatively induced reductive elimination reactions then re-form the C(sp)–C(sp^2^) *σ*-bonds to generate metathesis products. These studies culminated in a stoichiometric process for C–C *σ*-bond metathesis between diphenylacetylene and various diarylacetylenes.

## Abstract 2

The first example of a direct C(sp)–C(sp^2^) *σ*-bond metathesis occurs at a diiron complex. Following *σ*-bond oxidative addition, catalytic aryl-group exchange by Lewis acids precedes oxidatively induced reductive elimination to yield the products.

## Graphical abstract

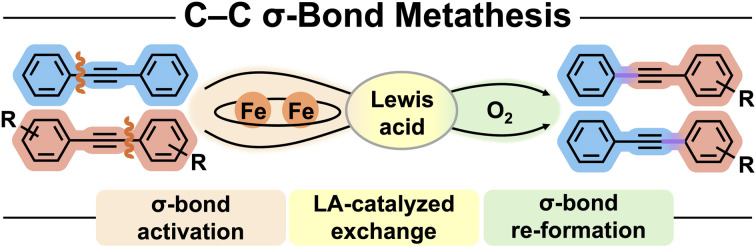

## Introduction

**Fig. 1:**
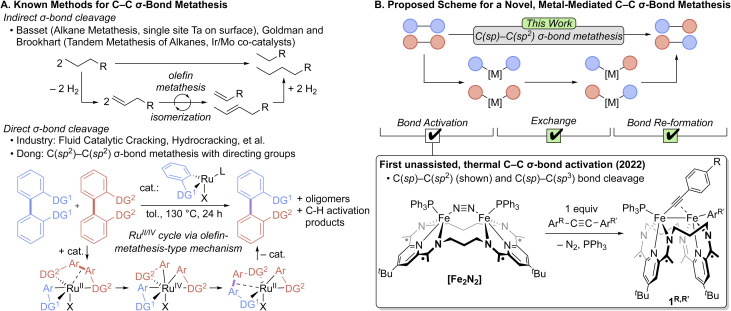
Carbon–carbon *σ*-bond metathesis processes. (A) Known methods for C–C *σ*-bond metathesis. (B) Proposed scheme for a novel, metal-mediated C–C *σ*-bond metathesis.

Olefin and alkyne metathesis have had an enormous impact on academic and industrial chemistry by allowing for the facile and selective rearrangement of C–C linkages (Fig. 1A) that were once thought to be inert to such transformations.^1–4^ Comparatively little is known about selective C–C *σ*-bond metathesis, in which C(sp^*x*^)–C(sp^*y*^) (*x*, *y* = 1, 2, 3) *σ*-bond partners are rearranged in a controlled manner.^5^ Industrial cracking chemistry, while effective, is energy-intensive and offers low selectivity.^6,7^ A pair of impressive methods for molecular, metal-mediated C(sp^3^)–C(sp^3^) *σ*-bond metathesis have been reported (Fig. 1A).^8,9^ However, these systems notably require forcing conditions and operate through key olefin metathesis steps, thus limiting their scope and selectivity. A few methods have recently been developed to effect the metathesis of specific types of C–C *σ*-bonds, provided certain assistive conditions are present, like anchimeric effects to aid in bond activation^10–12^ and the presence of a diketone chromophore to promote a metal-free, photochemical process.^13^ In work most relevant to the present study, Dong and co-workers reported Ru catalysts that perform *σ*-bond metathesis of the inter-aryl C(sp^2^)–C(sp^2^) bond of substituted biphenyls.^14^ They determined that this reaction operates through an olefin-metathesis type mechanism, in which the catalyst contains a fragment of the metathesis reaction and uses this fragment to accomplish the exchange process. This is an exciting development, but these reactions have notable drawbacks, including the need for forcing conditions and 2,2′-based directing groups on the substrates. These systems also produce substantial quantities of oligomeric and C–H activation-based side products, highlighting the difficulty of achieving selectivity in such reactions. The development of novel strategies for selective C–C *σ*-bond metathesis for any type of C(sp^*x*^)–C(sp^*y*^) linkage remains a significant outstanding challenge in synthetic chemistry.

We previously demonstrated the thermal activation and hydrofunctionalization of C(sp)–C(sp^2/3^) *σ*-bonds by the macrocycle-supported diiron complex, (^3^PDI_2_)Fe_2_(*µ*-N_2_)(PPh_3_)_2_ ([Fe_2_N_2_], Fig. 1B).^15,16^ Doing so formed the isolable dinuclear complexes (^3^PDI_2_)Fe_2_(*µ*-CCE)(E′)(PPh_3_) (E/E′ = aryl, alkyl), which feature bridging acetylide and terminal aryl/alkyl groups. This bond activation process was proposed to proceed through a *µ*-1,2-*η*^2^-bound alkyne complex, as observed with an isolable dicobalt analog,^17^ and was aided by the redox capacity of the pyridyldiimine units in the macrocycle. We reasoned that the diiron system could form the basis for a *σ*-bond metathesis process. To realize this goal, puzzles associated with acetylide/aryl/alkyl-group exchange between diiron centers and C–C bond re-formation would need to be overcome. Herein, we report on our initial success with these pursuits, yielding a C–C *σ*-bond metathesis process that operates through direct, thermodynamically favored C–C *σ*-bond activation, intermolecular exchange, and *σ*-bond re-formation steps. The chemistry, when applied to diarylacetylenes, is selective for metathesis at C(sp)–C(sp^2^) linkages and is successful toward a broad range of substrates with diverse electronic and structural profiles.

## Result and discussion

**Scheme 1:**
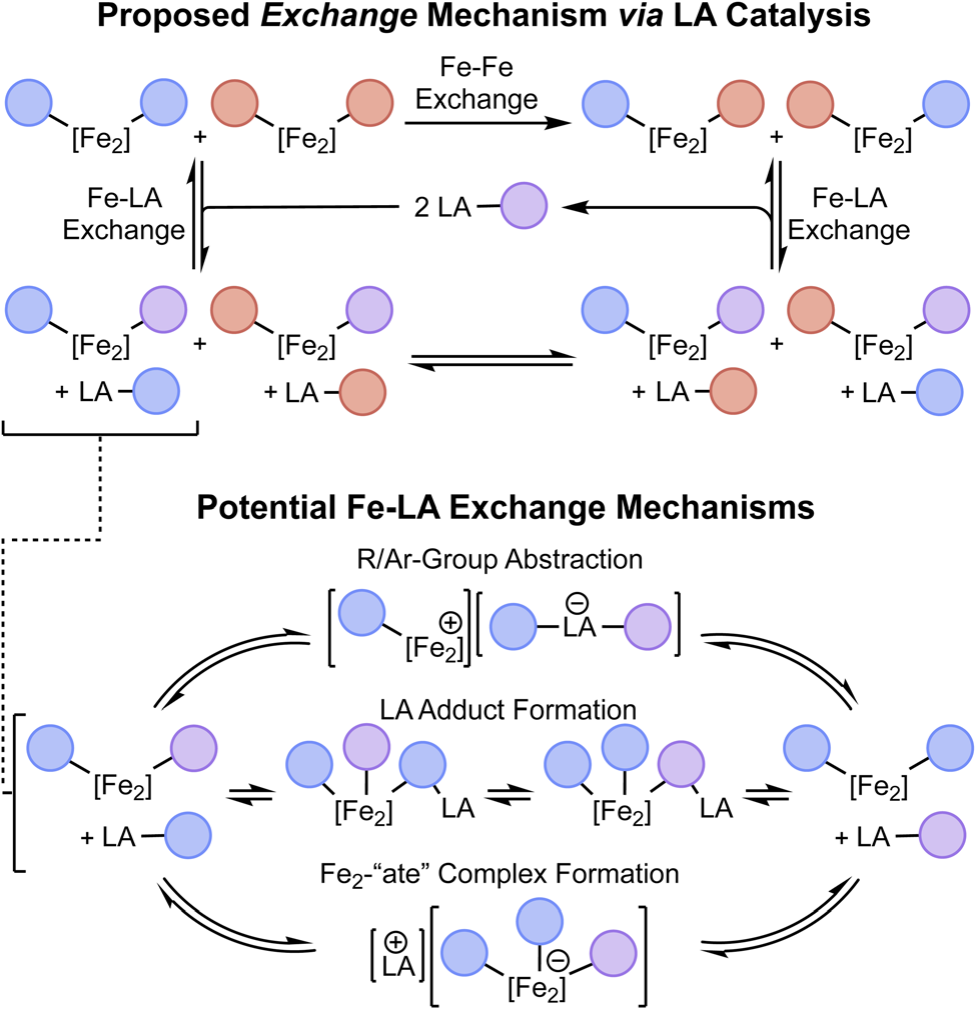
Top: Graphical illustration of the proposed Fe–Fe exchange reaction as catalyzed by a LA through sequential Fe–LA exchange reactions. Bottom: Various proposed Fe–LA exchange mechanisms.

The proposed scheme outlined in Fig. 1B envisions three steps for direct *σ*-bond metathesis: bond activation, exchange, and bond re-formation. Having previously demonstrated the bond activation step through the oxidative addition of C(sp)–C(sp^2^) bonds at [Fe_2_N_2_] to form (^3^PDI_2_)Fe_2_(*µ*-CCAr^R^)(Ar^R′^)(PPh_3_) (1^R,R′^; Ar^R^ = 4-R-C_6_H_4_), we next sought to identify a method of exchanging the cleaved organic fragments between 1^R,R′^ diiron complexes as a way to achieve the exchange portion of the proposed scheme. As outlined in Scheme 1 (top), we have termed this exchange of Fe-bound aryl/acetylide groups between two different C–C activation complexes as “Fe–Fe exchange” (*e.g.*1^R,R^ + 1^R′,R′^ → 1^R,R′^ + 1^R′,R^) and note that it presents a unique challenge from that of olefin/alkyne metathesis, for which the activation and exchange components of the process occur in reactions akin to the microscopic reverse of one another (*i.e.* cycloaddition/cycloreversion). In the present system, it was trivial to determine that the desired exchange of aryl/acetylide groups between diiron complexes does not occur spontaneously under thermal conditions in the absence of additives. We next considered the use of a Lewis acid (LA) to catalyze the Fe–Fe exchange reaction. This presents yet another layer of exchange, so to further disambiguate the complex series of reactions under discussion, the exchange of aryl/acetylide groups between a diiron complex and a Lewis acid is referred to here as “Fe–LA exchange” (Scheme 1).

We envisioned that Fe–LA exchange could operate through various potential mechanisms, including the direct abstraction of an aryl/acetylide group from 1, the formation of a LA adduct with 1, or the delivery of an anion to 1 to generate an “ate” complex (Scheme 1, bottom). To evaluate the feasibility of this process as a whole, we initially performed stoichiometric reactions between 1^H,H^ and traditional organometallic reagents. As will be described presently, we were gratified to find that this Fe–LA exchange process proceeded rapidly and achieved an appreciable equilibrium as would be required for a LA to operate within an Fe–Fe exchange scheme. The specific mechanism by which the LA mediates this exchange will be discussed below.

**Scheme 2:**
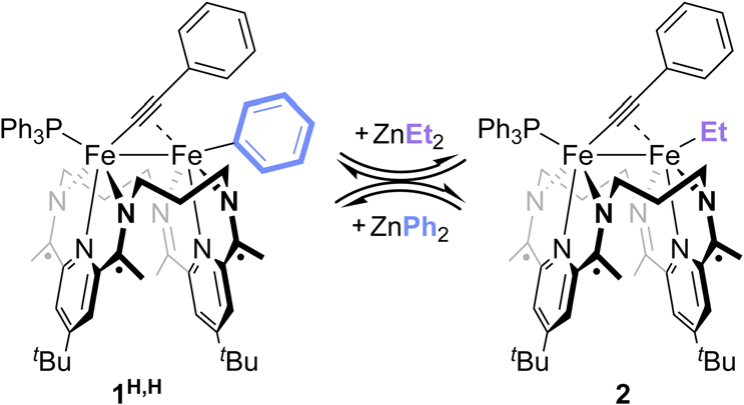
Stoichiometric Fe–LA exchange with organometallic reagents.

Treatment of 1^H,H^ with 1 equiv. of ZnEt_2_ resulted in the rapid appearance of (^3^PDI_2_)Fe_2_(*µ*-CCPh)(Et)(PPh_3_) (2) in the mixture's ^1^H and ^31^P{^1^H} NMR spectra. This result is indicative of a successful Fe–LA exchange reaction (Scheme 2 and Fig. S54) and suggests that the Fe-bound aryl group is more available to exchange than the acetylide group. The conversion of 1^H,H^ to 2 reached a maximum of 31% in 1 h. Further reaction monitoring revealed that the concentrations of 1^H,H^ and 2 maintained a *ca.* 2 : 1 ratio over time. Treatment of 1^H,H^ with superstoichiometric amounts of ZnEt_2_ resulted in increased conversion to 2, suggesting the presence of an equilibrium reaction mixture, though this could not be determined quantitatively due to slow *β*-H elimination from the Fe–Et unit (Fig. S55–58). Finally, treatment of isolated 2 with ZnPh_2_ led to the formation of 1^H,H^ in a similar, apparent equilibrium mixture, with 2 not being completely consumed until 3 equiv. of ZnPh_2_ had been added (Fig. S59). These data suggest that Zn^2+^ ions and organozinc reagents undergo Fe–LA exchange, prompting us to investigate the extent to which Lewis acids can catalyze Fe–Fe exchange through a series of reversible Fe–LA exchange processes.

**Fig. 2:**
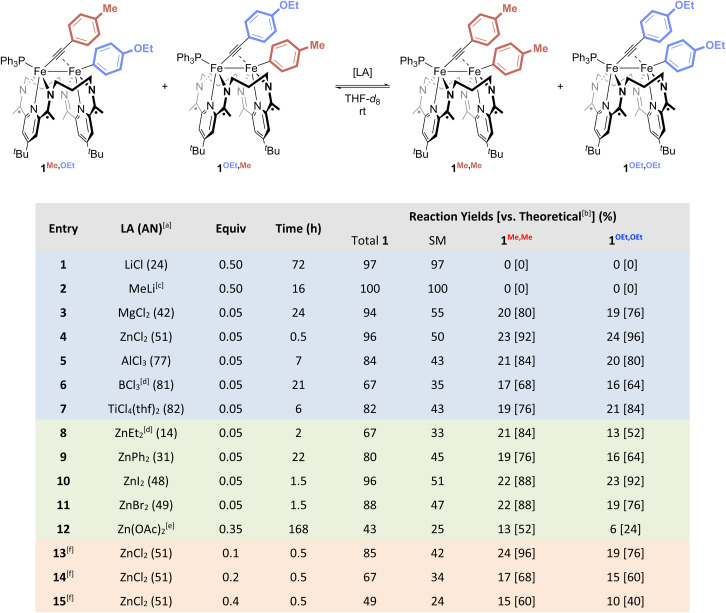
Lewis acid screening for the Fe–Fe exchange reaction, showing various reaction yields based on the use of different metals (top, blue), anions (middle, green), and loadings (bottom, orange) of Lewis acids. ^*a*^Lewis acid acceptor number, as determined by the Gutmann–Beckett method^18^ (see SI). ^*b*^Percent yield with respect to the theoretical yield of 25% that would be obtained from a 1 : 1 : 1 : 1 distribution of starting materials and products. ^*c*^Reaction performed in C_6_D_6_. ^*d*^Used as *n*-hexane solution. ^*e*^Sparingly soluble. ^*f*^Data obtained by the sequential addition of ZnCl_2_ from entries 13–15, followed by time to achieve a new equilibrium.

Next, to perform the Fe–Fe exchange reaction screening, we sought ready access to a mixture of two activation complexes, 1^R,R′^ and 1^R′,R^, which, following exposure to an appropriate Lewis acid, would generate the two exchange products 1^R,R^ and 1^R′,R′^ (Fig. 2, top). The desired mixture was generated through the treatment of [Fe_2_N_2_] with 1 equiv. of Ar^Me^CCAr^OEt^ (see SI). Doing so resulted in a gradual color change from red to green, along with the formation of two new signals in the mixture's ^31^P{^1^H} NMR spectrum at 62.2 and 62.4 ppm in a *ca.* 55 : 45 ratio. Further NMR spectroscopic and crystallographic studies revealed these signals to result from a mixture of 1^Me,OEt^ and 1^OEt,Me^, respectively, each of which was formed by the activation of one of the C(sp)–C(sp^2^) bonds present within the starting alkyne.

**Fig. 3:**
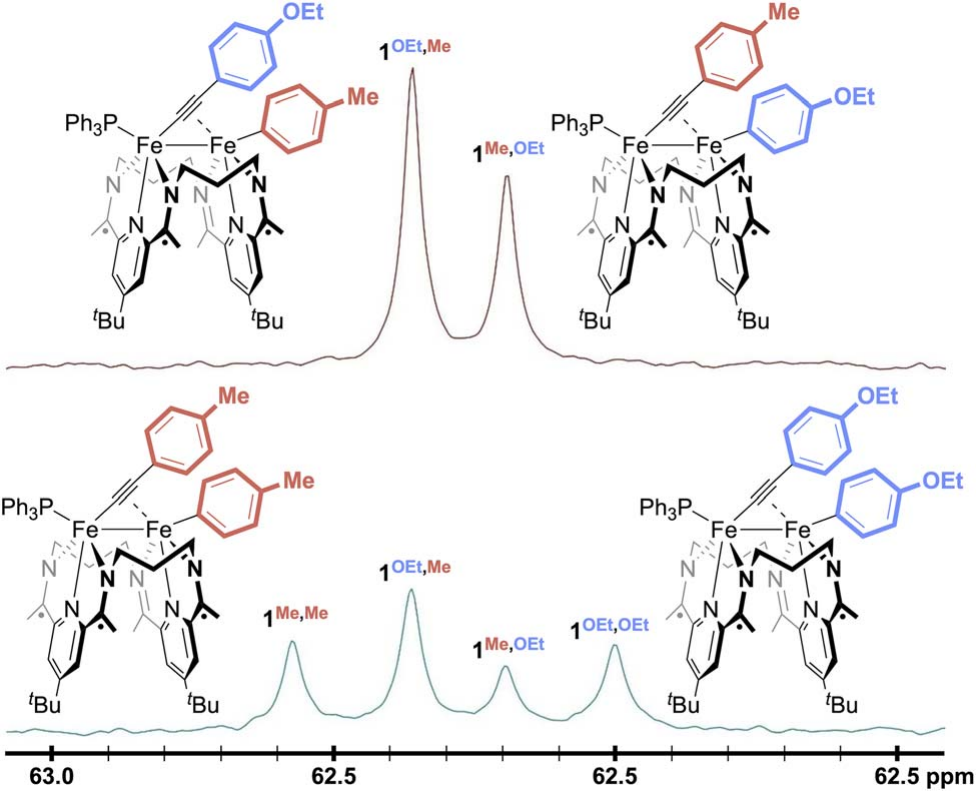
^31^P{^1^H} NMR spectra of a mixture of 1^OEt,Me^ and 1^Me,OEt^ before (top) and after (bottom) the addition of 5 mol% ZnCl_2_ in THF-*d*_8_ at 298 K.

No reactivity to form 1^Me,Me^ and 1^OEt,OEt^ was observed between 1^Me,OEt^ and 1^OEt,Me^ in solution (Fig. 3, top), but treatment of the roughly equimolar mixture with catalytic loadings of various Lewis acid chloride salts (5 mol% with respect to the total starting material) caused rapid changes in the reaction mixture (Fig. 3, bottom; Fig. S43–53). Monitoring the solutions by ^31^P{^1^H} NMR spectroscopy revealed the formation of two new features (62.0, 62.6 ppm). These were confirmed through independent syntheses to result from the presence of 1^Me,Me^ and 1^OEt,OEt^, indicative of successful Fe–Fe exchange processes. In all cases, the signals for 1^Me,OEt^ and 1^OEt,Me^ decreased in intensity during the reaction but did not disappear. The final ratios of the concentrations of 1^Me,OEt^, 1^OEt,Me^, 1^Me,Me^, and 1^OEt,OEt^ were close to 1 : 1 : 1 : 1, suggesting the system is under thermodynamic control and that the Ar^Me^ and Ar^OEt^ groups exert comparable steric and electronic effects on the thermodynamics of the system. This means the maximum yield expected for each of the products, 1^Me,Me^ and 1^OEt,OEt^, would be *ca.* 25%.

Alkali metal ions (Fig. 2, entries 1 and 2) were unable to mediate Fe–Fe exchange under the reaction conditions investigated. The chloride salts of several higher valent ions – MgCl_2_, ZnCl_2_, AlCl_3_, and TiCl_4_ – were found to be competent for Fe–Fe exchange (entries 3–5 and 7), but ZnCl_2_ provided superior yields in substantially shorter reaction times. A screen of the identity of the anion associated with Zn(ii) revealed that chloride is not essential to the Fe–Fe exchange process. Other halide and carboxylate anions provide exchange products (entry 8–12), though chloride was the most effective of those studied to date. Comparison of the Gutmann acceptor numbers (ANs, see SI) for these Lewis acids revealed optimal performance at mid-range ANs (∼35–70). These data are consistent with an Fe–Fe exchange mechanism that relies primarily on the electron-accepting nature of the Lewis acid, presumably through involvement of the Lewis acid in the formation and cleavage of Fe–Ar *σ*-bonds. Notably, on screening the catalyst loading up to 40 mol%, we observed a progressive decrease in the yield of 1^Me,Me^ and 1^OEt,OEt^ at higher catalyst loadings, albeit without loss of catalytic activity (entry 13–15, see SI). Higher catalyst loadings were found, more generally, to decrease the amount of all four C–C activation complexes, suggesting that ZnCl_2_ reversibly reacts with members of compound class 1^R,R′^ to form a complex 1^R,R′^ : LA. To gain further information on the nature of 1^R,R′^ : LA, we performed stoichiometric studies and investigated the products through spectroscopic and crystallographic analyses.

**Fig. 4:**
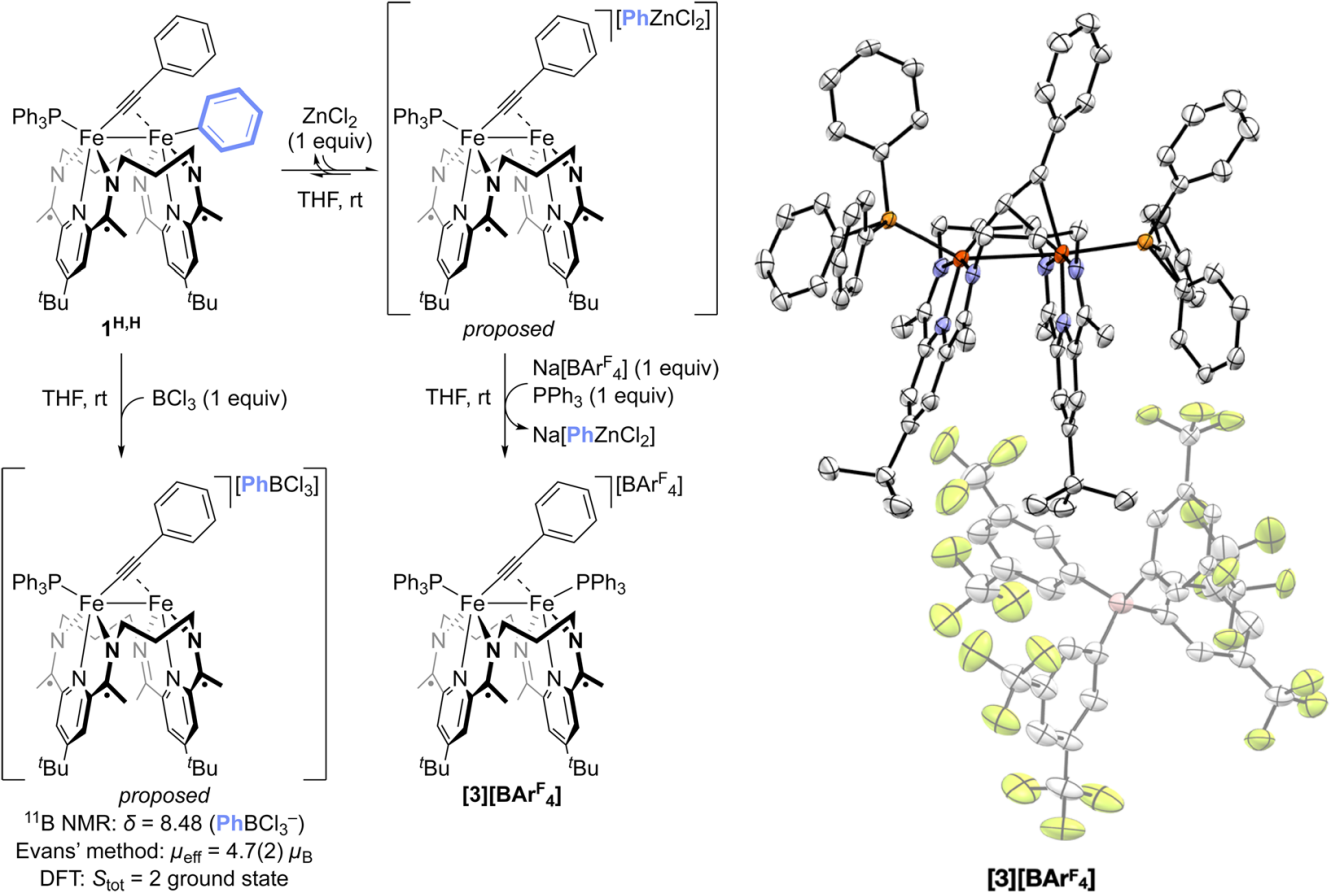
Fe–Fe exchange process overview. Left: Supporting stoichiometric reactivity, including aryl group abstraction from 1^H,H^ by different Lewis acids. Right: Thermal ellipsoid plot (50% probability level) of [3][BAr^F^_4_]. Hydrogens and solvent are removed for clarity.

On addition of an equimolar amount of ZnCl_2_ to a THF solution of a roughly 1 : 1 mixture of 1^Me,OEt^ and 1^OEt,Me^, the system became paramagnetic as determined by ^1^H NMR spectroscopy. The subsequent addition of 1^H,H^ led to the formation of 1^Me,Me^ and 1^OEt,OEt^, as determined by ^31^P{^1^H} NMR spectroscopy (Fig. S23), indicating that the product mixture of ZnCl_2_ and 1^Me,OEt^ and 1^OEt,Me^ remained competent for Fe–Fe exchange. This suggests that the interaction between 1^R,R′^ and ZnCl_2_ is reversible. Further, the treatment of 1^H,H^ with 1 equiv of BCl_3_, a stronger Lewis acid than ZnCl_2_, resulted in the rapid formation of a new species with a sharp signal in the material's ^11^B NMR spectrum at 8.48 ppm (THF-*d*_8_, 298 K). This signal is consistent with the reported chemical shift of the PhBCl_3_^−^ anion^19^ (Fig. 4 and S24) and is indicative of the abstraction of a Ph^−^ moiety from 1^H,H^ by BCl_3_. The paramagnetic nature of the system (*µ*_eff_ = 4.7(2)*µ*_B_), presumed to contain the cationic monophosphine species [(^3^PDI_2_)Fe_2_(*µ*-CCPh)(PPh_3_)]^+^ (*S*_tot_ = 2 ground state by DFT), prevented identification of the diiron portion of the sample by ^1^H NMR spectroscopy. However, treatment of the 1^H,H^ : ZnCl_2_ system with 1 equiv. of NaBAr^F^_4_ and 1 equiv. of PPh_3_ allowed for the isolation of the cationic diphosphine complex [(^3^PDI_2_)Fe_2_(*µ*-CCPh)(PPh_3_)_2_][BAr^F^_4_] ([3][BAr^F^_4_], Fig. 4 and S39; *µ*_eff_ = 2.6(2)*µ*_B_). This result is indicative of the reversible abstraction of an Fe-bound Ph^−^ moiety by ZnCl_2_, followed by anion exchange of PhZnCl_2_^−^ with BAr^F^_4_^−^ and coordination of an additional equivalent of PPh_3_. Since both BCl_3_ and ZnCl_2_ were found to be competent for mediating Fe–Fe exchange, these results indicate that aryl group abstraction is available to the Fe–Fe exchange process. Finally, the addition of PPh_3_ to the catalytic reaction mixture slows the rate of the Ar group exchange process, consistent with the identification of [3]^+^ as an off-cycle intermediate in Fe–Fe exchange.

**Fig. 5:**
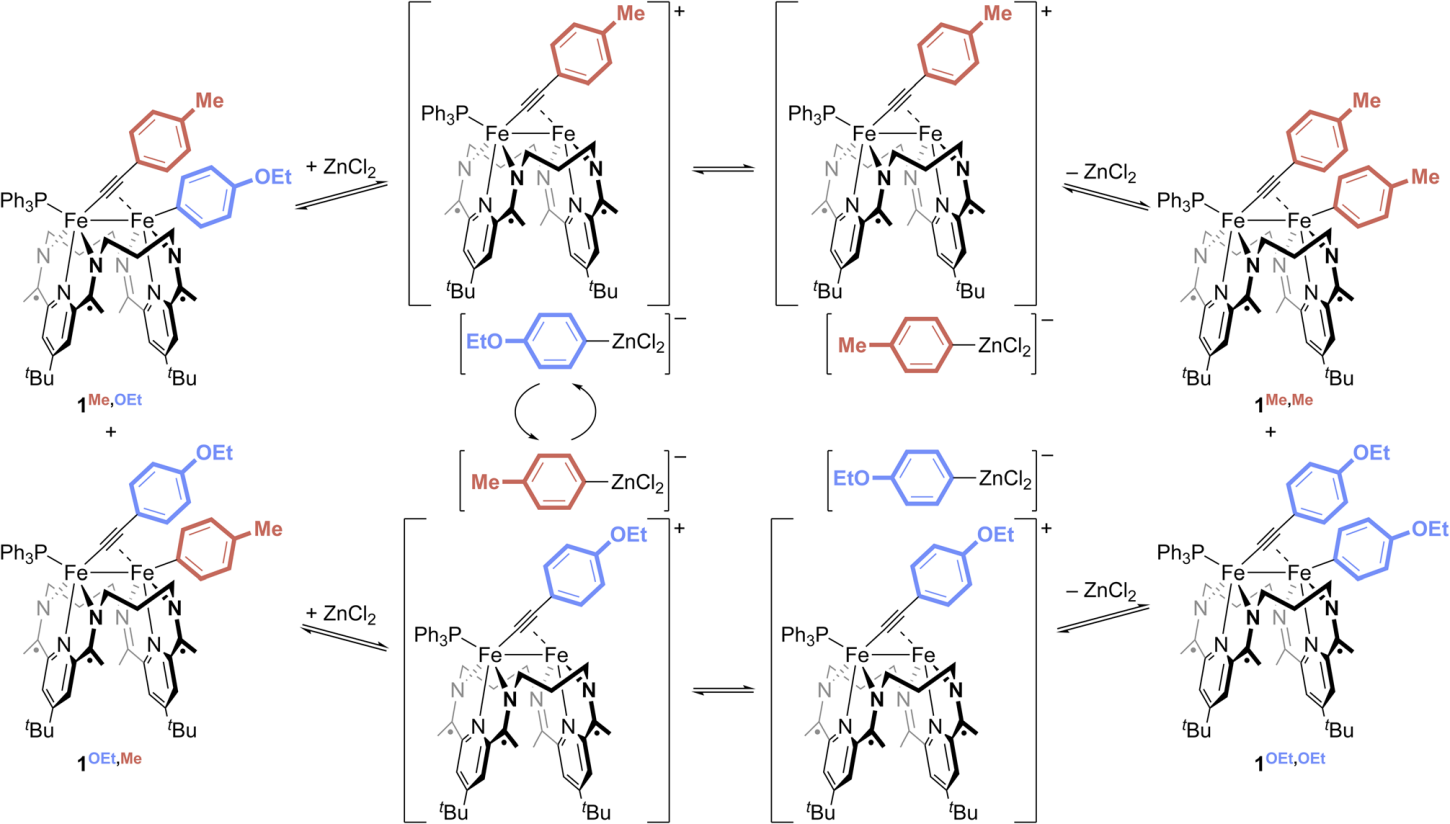
Proposed reaction scheme for Fe–Fe exchange.

DFT computations were used to validate the feasibility of aryl group abstraction from 1^H,H^ to form [3]^+^. All computations were performed on model complexes for which the ligand ^*t*^Bu groups were truncated to H, a modification that we have previously found to have little impact on the predictive capacity of the system.^15,16^ The abstraction of a Ph group from 1^H,H^ by ZnCl_2_ to form both the cationic, open coordination site complex [(^3^PDI_2_)Fe_2_(*µ*-CCPh)(PPh_3_)]^+^ and the zincate anion [PhZnCl_2_]^−^ was calculated to result in a 2.6 kcal mol^−1^ increase in the free energy of the system. Varying the identity of the Lewis acid revealed qualitative trends that scale with the acid's acceptor number (Fig. S66). Attempts to identify a transition state for the abstraction were unsuccessful, but [(^3^PDI_2_)Fe_2_(*µ*-CCPh)(PPh_3_)]^+^ is predicted to be favorably saturated by coordinating an additional equivalent of PPh_3_ (Δ*G* = −6.4 kcal mol^−1^). Finally, computations were used to probe the potential for alternative mechanisms by which the Lewis acid salt may activate complexes 1^R,R′^. In addition to the general mechanisms outlined in the bottom of Scheme 1, we also investigated the potential for Lewis-acid mediated abstraction of the phosphine to open a reactive coordination site. None of these alternative pathways provided reasonable energy profiles (see SI), which, together with the experimental data supporting the ability of Lewis acids to abstract an aryl group from 1^R,R′^, support an Fe–Fe exchange mechanism involving reversible aryl group abstraction and [Ar–LA]^−^ exchange (Fig. 5).

**Fig. 6:**
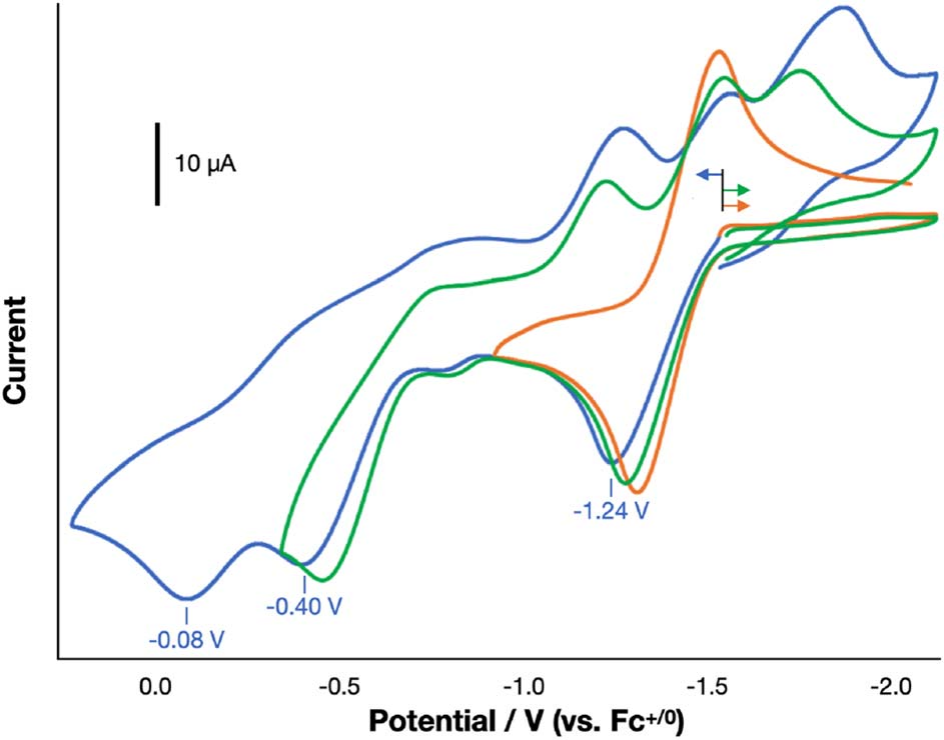
Cyclic voltammogram data of the full window-sweep (blue), the sweep containing first- and second-oxidation features (green), and the isolated first-oxidation feature (orange) of 1^H,H^ under scan rate of 100 mV s^−1^ in THF at 298 K. Tetrabutylammonium hexafluorophosphate ([^*n*^Bu_4_N][PF_6_]) was used as the electrolyte. The plots show one trace from multi-cycle sweeps.

We next sought the regeneration of a free alkyne from 1^R,R′^, as needed to complete a *σ*-bond metathesis process. The complexes 1^R,R′^, derived from diarylacetylenes have not been observed to undergo thermally accessible reductive elimination. We thus turned to oxidatively induced reductive elimination as a means of biasing the energy landscape toward reductive elimination.^20–23^ A cyclic voltammogram of 1^H,H^ in THF revealed oxidation features of interest at −1.24, −0.40, and −0.08 V *vs.* Fc^+/0^ (Fig. 6), and we were gratified to find that treatment of a THF solution of 1^H,H^ with 3 equiv. of AgOTf (*E*_1/2_ = +0.41 V *vs.* Fc^+/0^)^24^ resulted in the formation of PhCCPh in 38% yield as determined by GC-MS (Fig. S25).

To identify the specific level of oxidation needed to achieve this result, we undertook an in-depth investigation into the oxidation chemistry. The treatment of a THF solution of 1^H,H^ with 1 equiv. of [Cp_2_Co][OTf] (−1.33 V *vs.* Fc^+/0^)^24^ led to rapid color change from green to brown-green over 15 min. ^1^H and ^31^P{^1^H} NMR spectroscopic data indicated the full consumption of starting material to form an unidentified paramagnetic product and little formation of PhCCPh (<1%). Consistent with this outcome, a selective CV scan about the first oxidation feature revealed it to be quasi-reversible, suggesting that the oxidation of 1^H,H^ with [Cp_2_Co][OTf] yields [1^H,H^]^+^, which does not reductively eliminate PhCCPh on the timeframe of the CV scan.

**Fig. 7:**
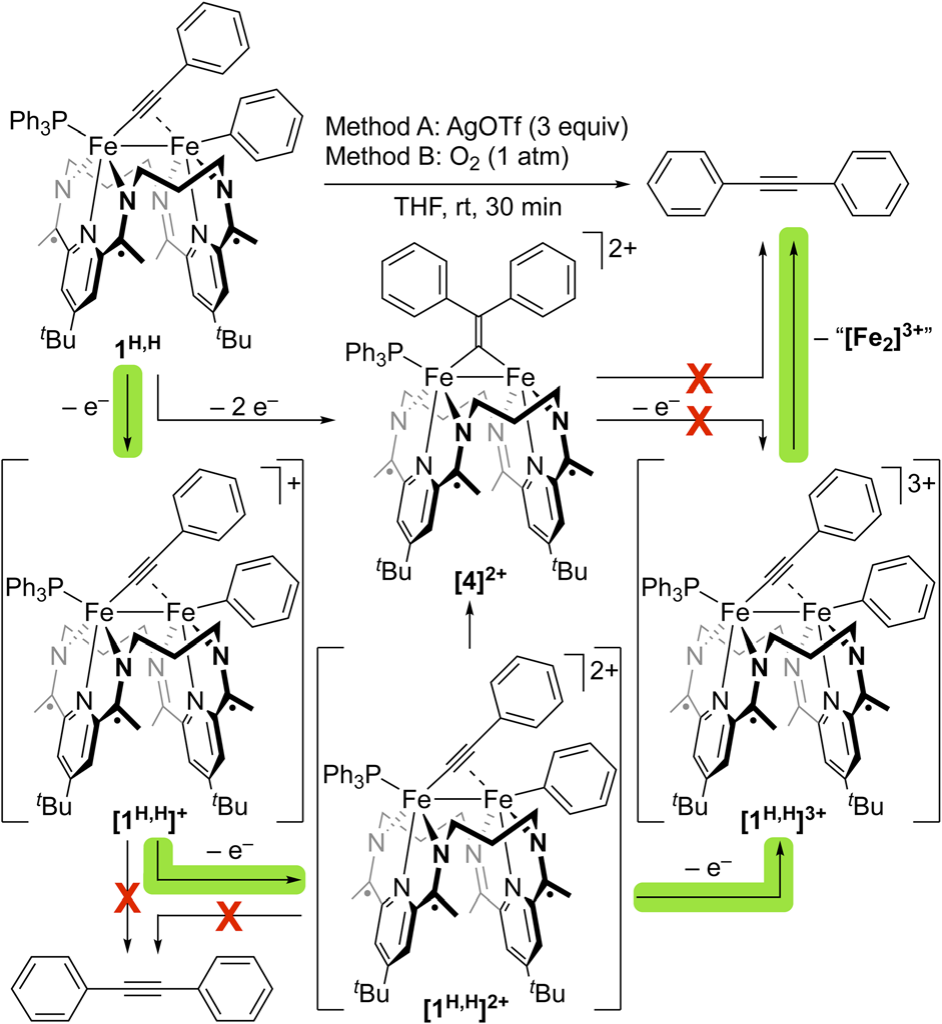
Overview of the potential pathways through which chemical oxidation of 1^H,H^ could generate PhCCPh *via* oxidatively induced reductive elimination.

The treatment of a THF solution of 1^H,H^ with 2 equiv. of FcPF_6_ led to a rapid color change from green to reddish brown. PhCCPh was again not observed by NMR spectroscopy, and a crystallographic study revealed the formation of a diiron bridging vinylidene species [(^3^PDI_2_)Fe_2_(*µ*-C=CPh_2_)(PPh_3_)](PF_6_)_2_ ([4][PF_6_]_2_, Fig. S40). This product appears to result from the migration of the Fe-bound Ph moiety to the *β*-position of the acetylide fragment and, importantly, indicates that a 3e^−^ oxidation from 1^H,H^ is needed to prompt the reductive elimination of PhCCPh. The appearance of [4]^2+^, however, raises a question about which species, [1^H,H^]^2+^ or [4]^2+^, is the immediate precursor to the C–C bond formation step that generates the alkyne. The migration of a vinylidene R/Ar-group to the *α*-carbon position has been shown previously to generate an *η*^2^-coordinated alkyne.^25^ However, the chemical oxidation of [4]^2+^ does not form PhCCPh (Fig. S60–63). This suggests that aryl-group migration to form [4]^2+^ from [1^H,H^]^2+^ competes with oxidation to [1^H,H^]^3+^, with the latter being the immediate precursor to the C–C reductive elimination process that forms PhCCPh (Fig. 7 and S60–63).

Finally, we sought to determine if O_2_, a low-cost and innocuous chemical oxidant, would be suitable for generating alkyne from 1^R,R′^. Exposure of a THF solution of 1^H,H^ to 1 atm of O_2_ for 30 min at room temperature resulted in the formation of PhCCPh in *ca.* 50% yield (Fig. S26), as determined by GC-MS, higher than the yield obtained through oxidation with 3 equiv. of AgOTf (38%, Fig. S25). Likewise, treatment of 1^OMe,OMe^ with 1 atm O_2_ afforded Ar^OMe^CCAr^OMe^ in a 60% spectroscopic yield, a marked improvement of the 28% spectroscopic yield obtained on treatment of 1^OMe,OMe^ with 3 equiv. of AgOTf (Fig. S27 and S28). These transformations suggested that O_2_ would serve as an effective oxidant, in combination with the C–C *σ*-bond activation and Fe–Fe exchange chemistry described above, for the direct metathesis of C(s*p*)–C(sp^2^) *σ*-bonds.

**Fig. 8:**
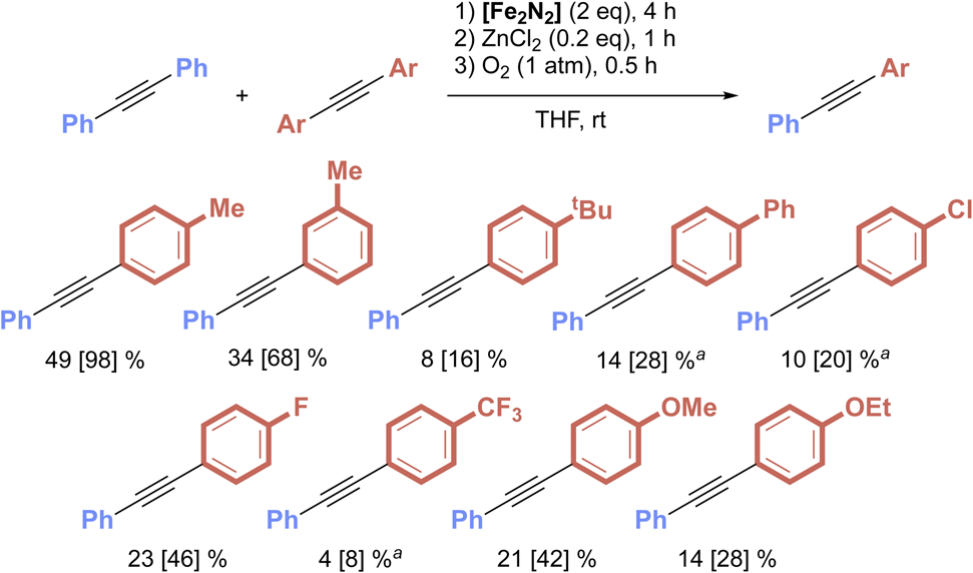
Substrate scope of the stepwise C–C *σ*-bond metathesis mediated by [Fe_2_N_2_], Lewis acid and oxidant. Yields were determined by NMR spectroscopy. The values in brackets represent the yield *vs.* a theoretical maximum of 50% and assuming thermodynamic control in the Fe–Fe exchange process. ^*a*^Yields determined by GC-MS.

Combining the elementary steps described above allowed us to perform C–C *σ*-bond metathesis between PhCCPh and various substituted diarylacetylenes, Ar^R^CCAr^R^ (Fig. 8). This was achieved by first combining the two alkynes with a stoichiometric amount of [Fe_2_N_2_] in THF, then adding, in sequence, catalytic quantities of ZnCl_2_ (10 mol%) and an excess of O_2_ (1 atm). The higher loading of ZnCl_2_ over the amount found to be optimal in the Fe–Fe exchange study (Fig. 2) was found to be needed due to the presence of free PPh_3_ generated during the *in situ* formation of 1^R,R^ from [Fe_2_N_2_]. Together, this process afforded the metathesis products PhCCAr^R^ in up to 49% yield (98% yield *vs.* expected), as determined by NMR spectroscopy and GC-MS analyses. In all cases, the presence of the starting material alkynes PhCCPh and Ar^R^CCAr^R^ were also observed, owing to the equilibrium nature of the Fe–Fe exchange reaction. The metathesis process tolerated a range of electronic differences, including electron-rich (R = OMe) and electron-poor (R = CF_3_) functionalities, as well as substitutions at the *meta*- and *para*-positions of the aryl ring. However, with either certain substituents (R = Br, NH_2_) or *ortho*-substituted aryl rings (2-Me or 2,4,6-Me_3_), the initial C–C activation process was not observed. It should also be noted that [Fe_2_N_2_] was found to be incompatible with a range of halide salts, including those that lack a Lewis acid cation (*e.g.* [^*n*^Bu_4_N][Cl]). This incompatibility will necessitate the identification of specialized Lewis acids to create a more integrated *σ*-bond metathesis process.

A trend in the product yield with respect to the electronic and steric properties of the R groups on the aryl rings was not observed. This is likely due to the intermediacy of multiple steps with comparable energy profiles that are all governed by the identity of the R groups in a non-complementary manner. Properties that favor oxidative addition may disfavor Fe–LA exchange and so on. The coordination spheres at Fe and the Lewis acid are also changing significantly during these processes, making the extent to which a given steric/electronic factor impacts each step vary from one step to the next. Furthermore, our earlier work revealed that the rate of the initial C–C oxidation addition process is highly dependent on the substrate's identity.^16^ The consequence is that variations in the rates of oxidative addition may yield mixtures that deviate from a 1 : 1 mixture of the two activation products within the 4 h timeframe given to step 1 of the metathesis process. For this reason and the other energy landscape considerations described above, the distribution of the four C–C activation products following Fe–Fe exchange may differ from a 1 : 1 : 1 : 1 ratio. Further research is needed to determine the underlying factors that control reaction selectivity in this system.

## Conclusions

In summary, this work describes the first example of a direct, metal-mediated C(sp)–C(sp^2^) *σ*-bond metathesis reaction. Using a macrocycle-supported diiron complex, [Fe_2_N_2_], in combination with a Lewis acid and an oxidant, the process achieves the one-pot bond activation, exchange, and bond re-formation steps needed to realize the metathesis of these linkages through a well-defined, thermodynamically controlled mechanism. Experimental and computational studies revealed the critical role Lewis acids play in the process by reversibly abstracting an Fe-bound aryl group to enable the key Fe–Fe exchange process at the heart of the metathesis reaction. The ability to reverse the bond activation step through oxidatively induced reductive elimination completed the *σ*-bond metathesis transformations. The reaction is tolerant of a range of electronic and steric substitutions on the aryl rings, suggesting this work may find use in an array of applications. Work is ongoing to apply these foundational studies to the development of *σ*-bond metathesis of other types of C–C linkages.

## Author contributions

T. L.: syntheses, reactivity investigation, writing – original draft and editing. J. E. G.: syntheses, reactivity investigation, writing – review. R. P. M.: theoretical investigation, writing – review. A. M. B.: structural analysis. M. R. G.: structural analysis. N. C. T.: conceptualization, supervision, project administration, funding acquisition, writing – original draft, review and editing.

## Conflicts of interest

The authors declare no competing interests.

## Supplementary Material

SC-017-D5SC10054B-s001

SC-017-D5SC10054B-s002

## Data Availability

CCDC 2375354–2375356 contain the supplementary crystallographic data for this paper.^26*a*–*c*^ The data supporting this article have been included in the supplementary information (SI). Supplementary information: compound syntheses, NMR and GC-MS spectra, molecular structures from single-crystal XRD studies, supplementary experiments, and XYZ coordinates of optimized structures. See DOI: https://doi.org/10.1039/d5sc10054b.
