# Supplementary material for: Direct metathesis of carbon–carbon σ-bonds at a versatile macrocycle-supported diiron platform
Source: Chem Sci. 2026 Feb 6;17(14):7203–10. doi: 10.1039/d5sc10054b (PMC12911874; doi:10.1039/d5sc10054b)
Supplement: SC-017-D5SC10054B-s001 [file SC-017-D5SC10054B-s001.pdf]

Supplementary Information (SI) for Chemical Science.
This journal is © The Royal Society of Chemistry 2026

Supporting Information for

Direct Metathesis of Carbon-Carbon σ-bonds at a Versatile Macrocycle-Supported Diiron Platform

Tianchang Liu, Jared E. Gonder, Ryan P. Murphy, Alexandra M. Bacon, Michael R. Gau and Neil C. Tomson

Department of Chemistry, University of Pennsylvania, Philadelphia, Pennsylvania, 19104, United States

Table of Contents

Experimental Details and Characterization Data 2
Stepwise C–C σ-bond Metathesis and GC-MS Spectroscopy Data 31
Summary of Crystal Data 40
Determination of the acceptor number of Lewis acids via NMR experiments. 43
NMR Spectroscopic Investigation of Fe-Fe Exchange Reactions. 45
Evaluation of the competence of $[4]^{2+}$ to form PhCCPh on oxidation. 63
Investigation of the effect of PPh_3 addition to the Fe-Fe exchange reaction. 66
Computational Studies 68
References 98

Experimental Details and Characterization Data

General Methods. All reactions involving transition metals were performed under an inert atmosphere of N_2 in a PureLab HE glovebox. Glassware, stir bars, filter aid (Celite) and 4 Å molecular sieves were dried in an oven at 150 °C for at least 12 h prior to use. Solvents were dried by passage through either a column of activated alumina (THF, diethyl ether) or both activated alumina and Q5 (*n*-pentane, *n*-hexane, toluene), then sparged with N_2 for 15 min and storage over 4 Å molecular sieves. Deuterated solvents were purchased from Cambridge Isotope Laboratories, Inc. and either used directly ($CDCl_3$) or dried over Na/benzophenone (C_6D_6, THF-d_8), then vacuum transferred and stored under N_2 over activated 4 Å molecular sieves. ($^3PDI_2$)Fe_2(μ-N_2)(PPh_3)$_2$ (**[Fe_2N_2]**),[1] ($^3PDI_2$)Fe_2(μ-CCPh)Ph(PPh_3) (**$1^{H,H}$**),[2] ($^3PDI_2$)Fe_2(μ-CCAr^{OMe})Ar^{OMe}(PPh_3) (**$1^{OMe,OMe}$**)[2] and ($^3PDI_2$)Fe_2(μ-CCPh)Et(PPh_3) (**2**)[2] were synthesized following previously published procedures. Diphenylacetylene (99%) was purchased from Thermo Scientific, recrystallized from Et_2O, and dried under vacuum for 16 h prior to use. 1,2-*bis*(4-methylphenyl)acetylene, 1,2-*bis*(3-methylphenyl)acetylene, 1,2-*bis*(4-*tert*-butylphenyl)acetylene, 1,2-*bis*(4-phenylphenyl)acetylene , 1,2-*bis*(4-methoxyphenyl)acetylene, 1,2-*bis*(4-ethoxyphenyl)ethyne, 1,2-*bis*(4-chlorophenyl)acetylene , 1,2-*bis*(4-fluorophenyl)acetylene, 1,2-*bis*(4-(trifluoromethyl)phenyl)ethyne, 1-(4-methylphenyl)-2-(4-ethoxyphenyl)acetylene were purchased from Ambeed and dried at 60 °C under vacuum for 16 h prior to use. 1-phenyl-1-propyne and 1-phenyl-1-butyne were purchased from Ambeed, vacuum distilled, and stored over activated 4 Å molecular sieves prior to use. 3-methyl-1-phenyl-but-1-yne, (3,3-dimethyl-but-1-ynyl)benzene, and 1-(phenylethynyl)-4-(trifluoromethyl)benzene were synthesized according to previous literature procedures[3-4] and vacuum distilled prior to use. Zinc chloride (98%), zinc bromide (98%), zinc iodide (98%), zinc acrylate (98%), magnesium chloride (98%), aluminum chloride (99%) were purchased from Millipore Sigma and dried under vacuum at 110 °C for 6 h prior to use. Titanium(IV) chloride tetrahydrofuran complex (97%), diethylzinc (1.0 M in *n*-hexane), diphenylzinc (98%) were purchased from Millipore Sigma and used without further purification. Boron trichloride (1.0 M in hexanes) was purchased from Millipore Sigma and used as received without further purification. 1,3,5-trimethoxybenzene (99%) and 1,2,4,5-tetramethylbenzene (97%) were purchased from Alfa Aesar, recrystallized twice from *n*-hexane, followed by drying at 60 °C under vacuum for 10 h prior to use. Triphenylphosphine (99%) was purchased from Alfa Aesar, recrystallized from Et_2O, followed by drying at 60 °C under vacuum for 10 h prior to use. Ferrocenium hexafluorophosphate (97%) was purchased from Millipore Sigma, recrystallized from DCM/*n*-hexane and dried under full vacuum at 60 °C for 6 h prior to use. Cobaltocenium triflate (98%) was purchased from Sigma-Aldrich and dried under full vacuum at 60 °C for 6 h prior to use. Ultra-high purity O_2 was purchased from Airgas and passed through a drying column equipped with Drierite® prior to use. Sodium tetrakis[3,5-bis(trifluoromethyl)phenyl]borate (NaBAr$^F_4$) was synthesized and purified according to a literature procedure.[5] A platinum wire working electrode (3.0 mm diameter) was purchased from BASi. A platinum wire counter electrode was purchased from Alfa Aesar. Clorox 30966 Concentrated Regular Bleach (7.55% NaClO) was purchased from Amazon. Tetrabutylammonium hexafluorophosphate was purchased from Fisher, recrystallized twice from hot ethanol, then dried at 60 °C under 30 mbar pressure for 10 h before storage in a glovebox under dry nitrogen prior to use. ^{1}H, ^{13}C{^{1}H}, ^{31}P{^{1}H}, ^{19}F{^{1}H}, ^{1}H-^{13}C{^{1}H} HSQC, ^{1}H-^{13}C{^{1}H} HMBC and ^{1}H-^{1}H COSY NMR spectra were recorded on Bruker UNI 400, AV3BIO 500, UNI 500, NEO 600 or CRYO 500 spectrometers. All chemical shifts (δ) are reported in units of ppm and referenced to the residual protio-

solvent resonance for proton and carbon chemical shifts.[6] External H_3PO_4, and $CFCl_3$ were used for referencing ^{31}P and ^{19}F NMR chemical shifts, respectively. Elemental analyses were performed at the CENTC elemental analysis facility at the University of Rochester. Solution phase effective magnetic moment data were determined using Evans' method.[7] Gas chromatography-mass spectrometry (GC-MS) was performed on either an Agilent 5973 GC-MS instrument or an Agilent 7890B GC coupled with a 5977A MS detector. Samples of the sigma-bond metathesis reactions were prepared by diluting the analyte with THF to 1.50 mL and filtering through Celite, then transferring into a Thermo Scientific 2 mL clear target DP vial with a screw septa cap.

X-ray Crystallography. Single-crystal X-ray diffraction data were collected on a Rigaku Synergy-S diffractometer with a HyPix-6000HE HPC area detector using Mo-Kα radiation (**[4][PF$_6$]$_2$**) and Cu-Kα radiation (**[3][BAr$^F_4$]**) or a Rigaku Synergy-S diffractometer with a Dectris Pilatus3 R 200K HPC area detector using Mo-Kα radiation (co-crystallized **1^{Me,OEt}** and **1^{OEt,Me}**, all at a temperature of 100 K. Rotation frames were integrated using CrysAlisPro[8], producing a listing of unaveraged F^2 and $\sigma(F^2)$ values. The intensity data were corrected for Lorentz and polarization effects and for absorption using SCALE3 ABSPACK.[9] The structures were solved by dual space methods using SHELXT[10] and refined by full-matrix least squares, based on F^2 using SHELXL-2018[11]. CCDC entries 2375354 (co-crystallized **1^{Me,OEt}** and **1^{OEt,Me}**), 2375355 (**[3][BAr$^F_4$]**), and 2375356 (**[4][PF$_6$]$_2$**) contain the supplementary crystallographic data for this paper. These data can be obtained free of charge from the Cambridge Crystallographic Data Centre via www.ccdc.cam.ac.uk/data_request/cif. Crystal parameters and refinement results are given in Table S1.

Electrochemistry. Cyclic voltammetry experiments were performed using a CH instruments 600B potentiostat. The data were processed with CHI600B Electrochemical Analyzer software version 15.08. All experiments were performed under an N_2 atmosphere in a Vigor glovebox (Model No. SG1800/750TS-F) using an electrochemical cell that consists of a platinum (3 mm outer diameter) working electrode, a platinum wire counter electrode, and a Ag/AgCl pseudo-reference electrode generated by dipping a silver wire into bleach for at least 30 s to enable sufficient coating of AgCl. All experiments were conducted in THF, with 3 mM analyte and 250 mM [^{n}Bu$_4$N][PF$_6$] as the supporting electrolyte. Potentials were reported versus $Cp_2Fe^{+/0}$, which was added as an internal standard for reference at the end of each experiment.

Synthesis of a 55:45 mixture of (^{3}PDI$_2$)Fe$_2$(*μ*-CCAr^{Me})(Ar^{OEt})(PPh$_3$) (1^{Me,OEt}) and (^{3}PDI$_2$)Fe$_2$(*μ*-CCAr^{OEt})(Ar^{Me})(PPh$_3$) (1^{OEt,Me}).

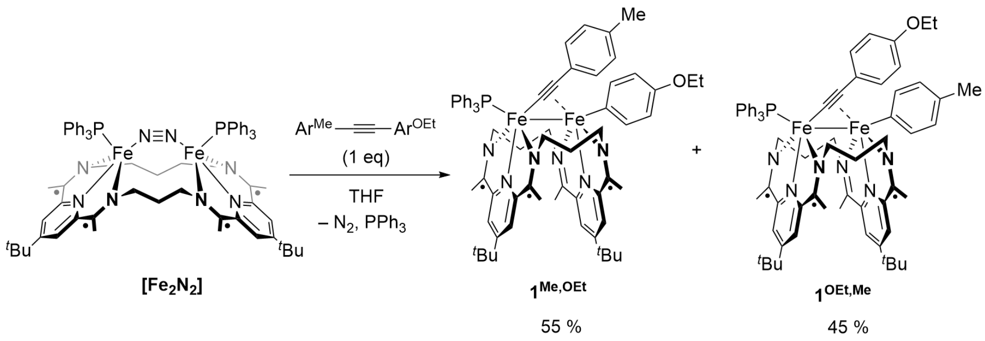

The addition of 1-(4-methylphenyl)-2-(4-ethoxyphenyl)acetylene (20.0 mg, 0.085 mmol) to a solution of **[Fe$_2$N$_2$]** (100.0 mg, 0.085 mmol) in 10 mL of THF at room temperature gradually formed a green solution after stirring for 16 h. All volatile materials were then removed under reduced pressure. The crude mixture was dissolved into 7 mL of toluene, filtered through Celite, layered with 10 mL of *n*-hexane, and stored at

-35 °C for 3 d to afford a mixture of $\mathbf{1}^{\mathbf{Me,OEt}}$ and $\mathbf{1}^{\mathbf{OEt,Me}}$ as green blocks. Yield: 56.0 mg (57 %). Crystals suitable for X-ray diffraction analysis were obtained from storage of a saturated Et_2O solution of the product mixture at -35 °C for 2 d. ^{1}H NMR (500 MHz, THF-d_8, 298 K): δ = 7.34 – 7.32 (m, 4H, CCAr*H*), 7.27 (s, 4H, py *m-H*), 7.24 – 7.20 (m, 6H, PPh_3 *p-H*), 7.18 – 7.16 (m, 4H, CCAr*H*), 7.13 – 7.08 (m, 24H, PPh_3 *o*, *m-H*), 7.02 (s, 2H, py *m-H*), 7.01 (s, 2H, py *m-H*), 6.95 (d, 2H, $^3J_{HH}$ = 8.0 Hz, CCAr*H*), 6.70 (d, 2H, $^3J_{HH}$ = 8.0 Hz, CCAr*H*), 6.03 (d, 2H, $^3J_{HH}$ = 8.0 Hz, CCAr*H*), 5.88 (d, 2H, $^3J_{HH}$ = 8.0 Hz, CCAr*H*), 5.83 (d, 2H, $^3J_{HH}$ = 8.0 Hz, CCAr*H*), 5.78 (d, 2H, $^3J_{HH}$ = 8.0 Hz, CCAr*H*), 4.21 – 4.14 (td, 2H, C*H*$_2$), 4.21 – 4.14 (td, 2H, C*H*$_2$), 4.05 – 3.99 (q, 2H, $^3J_{HH}$ = 6.8 Hz, O*Et*), 3.77 – 3.71 (td, 2H, C*H*$_2$), 3.64 – 3.60 (q, 2H, $^3J_{HH}$ = 6.8 Hz, Ar-O*Et*), 3.18 – 3.15 (m, 2H, C*H*$_2$), 2.33 (s, 3H, Ar-*Me*), 2.30 (s, 6H, Ar-*Me*), 2.20 – 2.18 (dt, 4H, C*H*$_2$), 2.09 (s, 12H, imine C*H*$_3$), 2.08 – 2.03 (dt, 4H, C*H*$_2$), 1.87 (s, 3H, Ar-*Me*), 1.64 (d, 12H, J_{PH} = 2.5 Hz, imine C*H*$_3$), 1.45 (s, 18H, C(C*H*$_3$)$_3$), 1.40 (t, 3H, Ar-O*Et*), 1.39 – 1.37 (dt, 4H, C*H*$_2$), 1.26 (s, 18H, C(C*H*$_3$)$_3$), 1.12 (t, 3H, Ar-O*Et*) ppm; ^{13}C NMR (126 MHz, THF-d_8, 298 K): δ = 192.18 (d, *C*CAr), 160.38 (s, C_{imine}), 156.33 (s, py*C*), 155.05 (s, py*C*), 153.62 (s, C_{imine}), 152.08 (d, C_{imine}), 152.00 (d, C_{imine}), 150.36 (d, py*C*), 150.28 (d, py*C*), 142.63 (s, py*C*), 142.54 (s, py*C*), 142.33 (s, py*C*), 142.28 (s, py*C*), 139.42 (s, py*C*), 139.22 (s, py*C*), 138.61 (s, CCAr*C*), 134.84 (d, PPh_3-Ph*C*), 134.62 (d, PPh_3-Ph*C*), 134.32 (d, PPh_3-Ph*C*), 134.24 (d, PPh_3-Ph*C*), 134.03 (s, CCAr*C*), 133.12 (s, CCAr*C*), 131.84 (s, CCAr*C*), 129.84 (s, PPh_3-Ph*C*), 129.61 (s, CCAr*C*), 129.20 (s, CCAr*C*), 129.08 (s, PPh_3-Ph*C*), 128.85 (s, CCAr*C*), 128.68 (d, PPh_3-Ph*C*), 128.49 (d, PPh_3-Ph*C*), 127.60 (s, Fe-Ar*C*), 126.21 (s, Fe-Ar*C*), 125.76 (s, Fe-Ar*C*), 114.24 (s, py*C*), 112.11 (s, py*C*), 111.39 (s, py*C*), 110.09 (s, py*C*), 109.56 (s, CCAr*C*), 63.92 (s, O*C*H$_2$CH$_3$), 63.10 (s, O*C*H$_2$CH$_3$), 59.30 (s, *C*H$_2$), 59.25 (s, *C*H$_2$), 53.53 (s, *C*H$_2$), 35.89 (s, *C*(CH$_3$)$_3$), 35.47 (s, *C*(CH$_3$)$_3$), 32.72 (s, *C*H$_2$), 32.22 (s, C(*C*H$_3$)$_3$), 32.11 (s, C(*C*H$_3$)$_3$), 21.66 (s, Ar*Me*), 21.08 (s, Ar*Me*), 15.71 (s, OCH$_2$*C*H$_3$), 15.63 (s, OCH$_2$*C*H$_3$), 14.47 (d, imine *C*H$_3$), 13.42 (d, imine *C*H$_3$) ppm. ^{31}P{^{1}H} NMR (162 MHz, THF-d_8, 298 K): δ = 62.37 (s, *P*Ph$_3$-$\mathbf{1}^{\mathbf{OEt,Me}}$), 62.20 (s, *P*Ph$_3$-$\mathbf{1}^{\mathbf{Me,OEt}}$) ppm. Anal. Calcd. for $C_{67}H_{77}Fe_2N_6OP$·toluene (1217.20 g/mol): C, 73.02; H, 7.04; N, 6.90. Found: C, 73.50; H, 7.18; N, 6.68. The material used for EA was crystallized from toluene and had one co-crystallized toluene molecule according to the sample's NMR spectrum.

Synthesis of (^{3}PDI$_2$)Fe$_2$(*μ*-CCAr^{Me})(Ar^{Me})(PPh$_3$) ($\mathbf{1}^{\mathbf{Me,Me}}$). The addition of 1,2-*bis*(4-methylphenyl)acetylene (17.5 mg, 0.085 mmol) to a solution of **[Fe$_2$N$_2$]** (100.0 mg, 0.085 mmol) in 10 mL of THF at room temperature gradually formed a green solution after stirring for 16 h. All volatile materials were then removed under reduced pressure. The crude mixture was dissolved into 7 mL of toluene, filtered through Celite, layered with 10 mL of *n*-hexane, and stored at -35 °C for 3 d to afford $\mathbf{1}^{\mathbf{Me,Me}}$ as green blocks. Yield: 31.8 mg (34 %). ^{1}H NMR (400 MHz, C_6D_6, 298 K): δ = 8.03 – 8.01 (m, 2H, CCAr *m-H*), 7.34 – 7.24 (m, 8H, PPh_3 *m-H* + CCAr *m-H*), 7.21 (s, 2H, py *m-H*), 7.03 – 6.95 (m, 11H, PPh_3 *o-H* + *p-H* + py *m-H*), 6.69 – 6.67 (m, 2H, Fe-Ar *m-H*), 6.44 – 6.42 (m, 2H, Fe-Ar *o-H*), 4.47 – 4.41 (td, 2H, C*H*$_2$), 3.95 – 3.89 (td, 2H, C*H*$_2$), 3.14 – 3.11 (dt, 2H, C*H*$_2$), 2.33 (s, 3H, Ar-*Me*), 2.14 – 2.11 (m, 5H, Ar-*Me* + C*H*$_2$), 1.76 – 1.67 (m, 8H, imine C*H*$_3$ + C*H*$_2$), 1.46 (s, 9H, C(C*H*$_3$)$_3$), 1.44 (d, 6H, $^2J_{PH}$ = 2.5 Hz, imine C*H*$_3$), 1.33 (s, 9H, C(C*H*$_3$)$_3$), 1.06 – 1.02 (dt, 2H, C*H*$_2$) ppm. ^{13}C{^{1}H} NMR (126 MHz, THF-d_8, 298 K): δ = 195.32 (d, $^2J_{PC}$ = 11.6 Hz, *C*CAr), 165.78 (d, $^4J_{PC}$ = 2.3 Hz, Fe-Ar*C*), 160.34 (s, C_{imine}), 153.61 (s, py*C*), 152.03 (d, $^4J_{PC}$ = 2.5 Hz, C_{imine}), 150.35 (d, $^3J_{PC}$ = 3.8 Hz, py*C*), 142.64 (s, py*C*), 142.41 (s, py*C*), 139.38 (s, Fe-Ar*C*), 138.60 (s, Fe-Ar*C*), 134.75 (d, $^1J_{PC}$ = 28.2 Hz, PPh_3-Ph*C*), 134.31 (d, $^2J_{PC}$ = 10.1 Hz, PPh_3-Ph*C*), 133.13 (s, CCAr*C*), 131.82 (s, CCAr*C*), 129.84 (s, PPh_3-Ph*C*), 128.63 (s, CCAr*C*), 128.49 (d, $^2J_{PC}$ = 8.3 Hz, PPh_3-Ph*C*), 127.61 (s, Fe-Ar*C*), 126.21 (s, CCAr*C*), 111.40 (s, py*C*), 110.28 (s, C*C*Ar), 110.13 (s, py*C*), 59.28 (s, *C*H$_2$), 53.53 (s, *C*H$_2$), 35.89 (s, *C*(CH$_3$)$_3$), 35.46 (*C*(CH$_3$)$_3$), 32.27 (s, *C*H$_2$), 32.22 (s, C(*C*H$_3$)$_3$), 32.10 (s, C(*C*H$_3$)$_3$), 21.66 (s, CCAr-*C*H$_3$), 21.08 (Fe-Ar-*C*H$_3$), 14.48 (s, imine *C*H$_3$), 13.43 (d, $^5J_{PC}$ = 1.6 Hz, imine *C*H$_3$) ppm. ^{31}P{^{1}H}

NMR (162 MHz, THF-d_8, 298 K): δ = 62.56 (s, *P*Ph$_3$) ppm. Anal. Calcd. for $C_{66}H_{75}Fe_2N_6P$ (1095.03 g/mol): C, 72.39; H, 6.90; N, 7.67. Found: C, 73.17; H, 7.11; N, 7.27.

Synthesis of (^{3}PDI$_2$)Fe$_2$(μ-CCAr^{OEt})(Ar^{OEt})(PPh$_3$) (1^{OEt,OEt}). The addition of 1,2-*bis*(4-ethoxyphenyl)ethyne (22.6 mg, 0.085 mmol) to a solution of **[Fe$_2$N$_2$]** (100.0 mg, 0.085 mmol) in 10 mL of THF at room temperature gradually formed a green solution after stirring for 16 h. All volatile materials were then removed under reduced pressure. The crude mixture was dissolved into 7 mL of THF, filtered through Celite, layered with 10 mL of *n*-hexane, and stored at -35 °C for 3 d to afford **1^{OEt,OEt}** as green blocks. Yield: 41.0 mg (41 %). ^{1}H NMR (400 MHz, C_6D_6, 298 K): δ = 7.33 (d, 2H, $^3J_{HH}$ = 8.5 Hz, CCAr-*H*), 7.27 (s, 2H, pyr-*H*), 7.23 – 7.20 (m, 3H, PPh$_3$), 7.11 – 7.08 (m, 12H, PPh$_3$), 7.01 (s, 2H, pyr-*H*), 6.72 (d, 2H, $^3J_{HH}$ = 8.5 Hz, CCAr-*H*), 5.88 (d, 2H, $^3J_{HH}$ = 8.5 Hz, FeAr-*H*), 5.79 (d, 2H, $^3J_{HH}$ = 8.5 Hz, FeAr-*H*), 4.20 – 4.15 (m, 2H, C*H*$_2$), 4.04 – 4.00 (q, 2H, $^3J_{HH}$ = 7.0 Hz, OC*H*$_2$CH$_3$), 3.76 – 3.71 (m, 2H, C*H*$_2$), 3.63 – 3.60 (q, 2H, $^3J_{HH}$ = 7.0 Hz, OC*H*$_2$CH$_3$), 3.18 – 3.16 (m, 2H, C*H*$_2$), 2.19 – 2.16 (m, 2H, C*H*$_2$), 2.09 (s, 6H, imine C*H*$_3$), 2.05 – 2.02 (m, 2H, C*H*$_2$), 1.63 (d, 6H, $^2J_{PH}$ = 2.5 Hz, imine C*H*$_3$), 1.45 (s, 9H, C(C*H*$_3$)$_3$), 1.41 – 1.38 (t, 3H, $^3J_{HH}$ = 6.5 Hz, OCH$_2$C*H*$_3$), 1.37 – 1.36 (m, 2H, C*H*$_2$), 1.26 (s, 9H, C(C*H*$_3$)$_3$), 1.13 (t, 3H, , $^3J_{HH}$ = 6.5 Hz, OCH$_2$C*H*$_3$) ppm. ^{13}C{^{1}H} NMR (126 MHz, THF-d_8, 298 K): δ = 192.05 (d, $^2J_{PC}$ = 11.6 Hz, *C*CAr), 160.34 (s, C_{imine}), 156.34 (s, CCAr*C*), 155.04 (s, FeAr-*C*), 153.55 (s, py*C*), 152.06 (d, $^4J_{PC}$ = 2.5 Hz, C_{imine}), 150.29 (d, $^3J_{PC}$ = 3.8 Hz, py*C*), 142.53 (s, py*C*), 142.20 (s, py*C*), 139.25 (s, FeAr-*C*), 138.61 (s, FeAr-*C*), 134.84 (d, $^1J_{PC}$ = 28.2 Hz, PPh$_3$-Ph*C*), 134.26 (d, $^2J_{PC}$ = 10.1 Hz, PPh$_3$-Ph*C*), 134.02 (s, CCAr*C*), 131.82 (s, CCAr*C*), 129.84 (s, PPh$_3$-Ph*C*), 128.84 (s, CCAr*C*), 128.47 (d, $^2J_{PC}$ = 8.3 Hz, PPh$_3$-Ph*C*), 126.21 (s, CCAr*C*), 114.29 (s, CCAr*C*), 112.11 (s, FeAr-*C*), 111.32 (s, py*C*), 110.03 (s, py*C*), 109.56 (s, C*C*Ar), 63.93 (CCAr-O*Et*), 63.13 (FeAr-O*Et*), 59.26 (s, *C*H$_2$), 53.54 (s, *C*H$_2$), 35.89 (s, *C*(CH$_3$)$_3$), 35.47 (*C*(CH$_3$)$_3$), 32.28 (s, *C*H$_2$), 32.22 (s, C(*C*H$_3$)$_3$), 32.11 (s, C(*C*H$_3$)$_3$), 15.72 (s, CCAr-O*Et*), 15.64 (FeAr-O*Et*), 14.48 (s, imine *C*H$_3$), 13.42 (d, $^5J_{PC}$ = 1.6 Hz, imine *C*H$_3$) ppm. ^{31}P{^{1}H} NMR (162 MHz, THF-d_8, 298 K): δ = 61.80 (s, *P*Ph$_3$) ppm. Anal. Calcd. for $C_{68}H_{79}Fe_2N_6O_2P$ (1155.08 g/mol): C, 70.71; H, 6.89; N, 7.28. Found: C, 69.31; H, 6.64; N, 7.04.

Synthesis of [(^{3}PDI$_2$)Fe$_2$(μ-CCPh)(PPh$_3$)$_2$][BAr^F$_4$] ([3][BAr^F$_4$]). To a stirred solution of **1^{H,H}** (50.0 mg, 0.047 mmol) in 10 mL of THF was added PPh$_3$ (12.3 mg, 0.047 mmol) and ZnCl$_2$ (6.3 mg, 0.047 mmol) at room temperature, resulting in a rapid color change from green to reddish brown. After being stirred for 30 min, NaBAr^F$_4$ was added to the solution and the mixture was stirred for another 15 min. All volatile materials were then removed under vacuum. The mixture was then extracted into 5 mL of Et$_2$O, filtered through Celite, and slowly evaporated into *n*-pentane at -35 ºC to afford **[3][BAr^F$_4$]** as dark brown blocks. Yield: 75 mg (72 %). ^{1}H NMR (500 MHz, THF-d_8, 298 K): δ = 8.45 (s, 8H, BAr^F$_4$ *o*-CH$_3$), 7.72 (s, 4H, BAr^F$_4$ *p*-CH$_3$), 6.96 (br, 18H, PPh$_3$ Ph-*H*), 6.83 (br, 12H, PPh$_3$ Ph-*H*), 1.34 (br, 18H, C(C*H*$_3$)$_3$) ppm. ^{11}B {^{1}H} NMR (128 MHz, THF-d_8, 298 K): δ = -6.37 (s, *B*Ar^F$_4$) ppm. ^{19}F{^{1}H} NMR (376 MHz, THF-d_8, 298 K): δ = -61.84 (s, BAr^F$_4$) ppm. Anal. Calcd. for $C_{114}H_{108}BF_{24}Fe_2N_6O_{1.5}P_2$ (2226.57 g/mol): C, 61.50; H, 4.89; N, 3.77. Found: C, 60.51; H, 4.58; N, 3.74. Evans' method: μ_{eff} = 2.6(2) μ_B.

Synthesis of [(^{3}PDI$_2$)Fe$_2$(μ-C=CPh$_2$)(PPh$_3$)][PF$_6$]$_2$ ([4][PF$_6$]$_2$). The addition of ferrocenium hexafluorophosphate (18.6 mg, 0.056 mmol) to a stirred solution of **1^{H,H}** (30.0 mg, 0.028 mmol) in 7 mL of THF at room temperature formed a reddish-brown solution over 2 h. All volatile materials were then removed under reduced pressure. The crude mixture was dissolved into 4 mL of THF, filtered through Celite, layered with 10 mL of *n*-hexane, and stored at room temperature for 3 d to afford **[4][PF$_6$]$_2$** as dark brown blocks. Crystals suitable for X-ray diffraction analysis were obtained from vapor diffusion of *n*-pentane into a saturated PhF solution of **[4][PF$_6$]$_2$**. Yield: 27.2 mg (71 %). ^{1}H NMR (500 MHz, THF-d_8,

298 K): δ = 67.28, 53.00, 20.08, 15.35, 13.29, 6.25, 5.01, 1.29, 0.26, -21.04 ppm. Anal. Calcd. for $C_{64}H_{71}F_{12}P_3Fe_2N_6Fe_2$ (1356.91 g/mol): C, 56.65; H, 5.27; N, 6.19. Found: C, 55.91; H, 5.86; N, 6.32. Evans' method: μ_{eff} = 2.8(2) μ_B.

Chemical oxidation of $1^{OMe,OMe}$ to afford $Ar^{OMe}CCAr^{OMe}$. Method A: To a stirred solution of **$1^{OMe,OMe}$** (31.4 mg, 0.028 mmol) in 7 mL of THF was added silver triflate (21.5 mg, 0.084 mmol) in the dark at room temperature, resulting in a rapid color change from green to dark brown. After stirring for 30 min, all volatile materials were removed under reduced pressure. The mixture was then extracted with Et_2O (5 mL x 2) and filtered through Celite. The filtrate was evaporated under reduced pressure to afford a white solid. NMR spectroscopic analysis revealed this solid to be $Ar^{OMe}CCAr^{OMe}$ and the yield was determined by using a known amount of 1,3,5-trimethoxybenzene (TMB) as an internal standard. Yield: 28%. **Method B:** **$1^{OMe,OMe}$** (10.1 mg, 0.009 mmol) was loaded into a 20 mL vial equipped with a stir bar. The material was dissolved with 7 mL of THF, then the vial was sealed with a septa-seal screw cap. The seal between the vial and the screw cap was reinforced by tightly taping around the joint with electrical tape. The vial was then removed from the glovebox, and the headspace within the vial was purged via a needle with O_2 for 5 min. The O_2 purge resulted in a rapid color change to brown and a precipitate formed. After stirring for 30 min, the vial was opened to air, and the mixture was filtered through Celite. All volatile materials were removed from the filtrate under reduced pressure to afford a pale-yellow solid. NMR spectroscopic data collected on the solid were consistent with the formation of $Ar^{OMe}CCAr^{OMe}$ and the yield was determined by use of a known amount of 1,3,5-trimethoxybenzene (TMB) as an internal standard. Yield: 60%.

Chemical oxidation of $1^{H,H}$ to afford PhCCPh. Method A: A 20 mL scintillation vial was charged with **$1^{H,H}$** (8.0 mg, 0.0075 mmol), 5 mL of THF, and a stir bar. To the stirring solution was added silver triflate (5.8 mg, 0.0225 mmol) as a solution in 1 mL of THF in the dark at room temperature, affording a color change from dark green to dark brown. The vial was capped, and the solution was allowed to stir for 30 min before being removed from the glovebox. The vial was then opened to air, and the mixture was filtered through Celite before the volatile materials were evaporated with a steady stream of air. To the remaining gray-brown residue was added 10 mL of hexane, and the mixture was sonicated for 10 min. The mixture was filtered through Celite and evaporated with a flow of air, affording a sticky, pale-yellow oil. The analyte was dissolved in 1.2 mL of *n*-hexane, transferred to a Thermo Scientific 2 mL clear target DP vial with a septum-sealed screw cap, and subjected to GC-MS analysis. Yield of PhCCPh: 38%. **Method B:** A 20 mL vial compatible with a septum-sealed screw cap was charged with **$1^{H,H}$** (8.0 mg, 0.0075 mmol), 6 mL of THF, and a stir bar. The vial was capped, and the seal was reinforced by tightly taping around the joint with electrical tape. The solution was allowed to stir for 5 min before being removed from the glovebox. The headspace within the vial was purged via a needle with O_2 for 5 min, and the resulting brown mixture was left to stir for 30 min. The vial was opened to air and, the mixture was filtered through Celite, then dried with a steady stream of air. To the remaining brown residue was added 10 mL of *n*-hexane, and the mixture was sonicated for 10 min. The mixture was filtered through Celite and evaporated with a flow of air, affording a yellow oil. The analyte was dissolved in 1.2 mL of *n*-hexane, transferred to a Thermo Scientific 2 mL clear target DP vial with a septum-sealed screw cap, and subjected to GC-MS analysis. Yield of PhCCPh: 50%.

NMR Spectroscopic Data

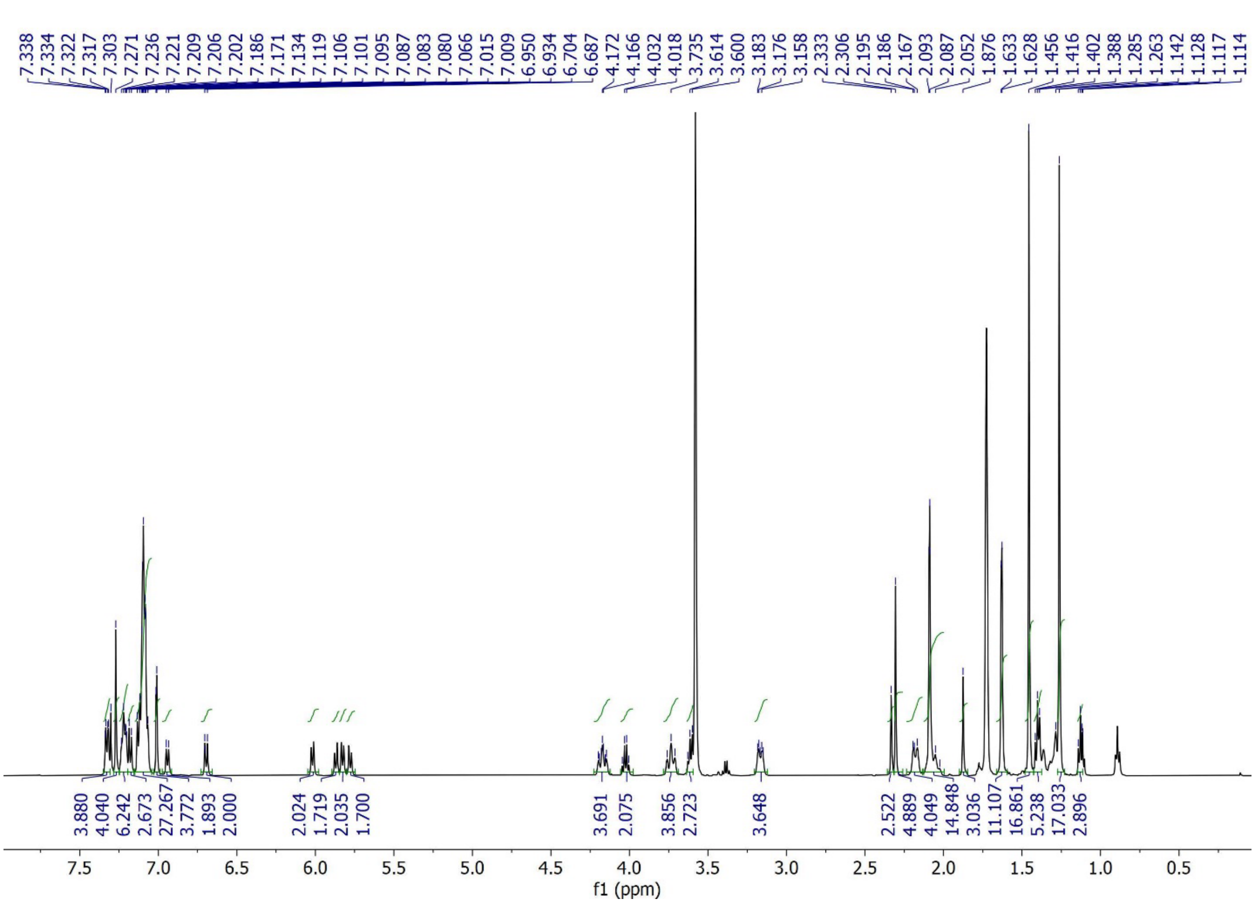

Figure S1. ^{1}H NMR spectrum of a 55:45 mixture of $\mathbf{1}^{\mathbf{Me,OEt}}$ and $\mathbf{1}^{\mathbf{OEt,Me}}$ in THF-d_8 (298 K).

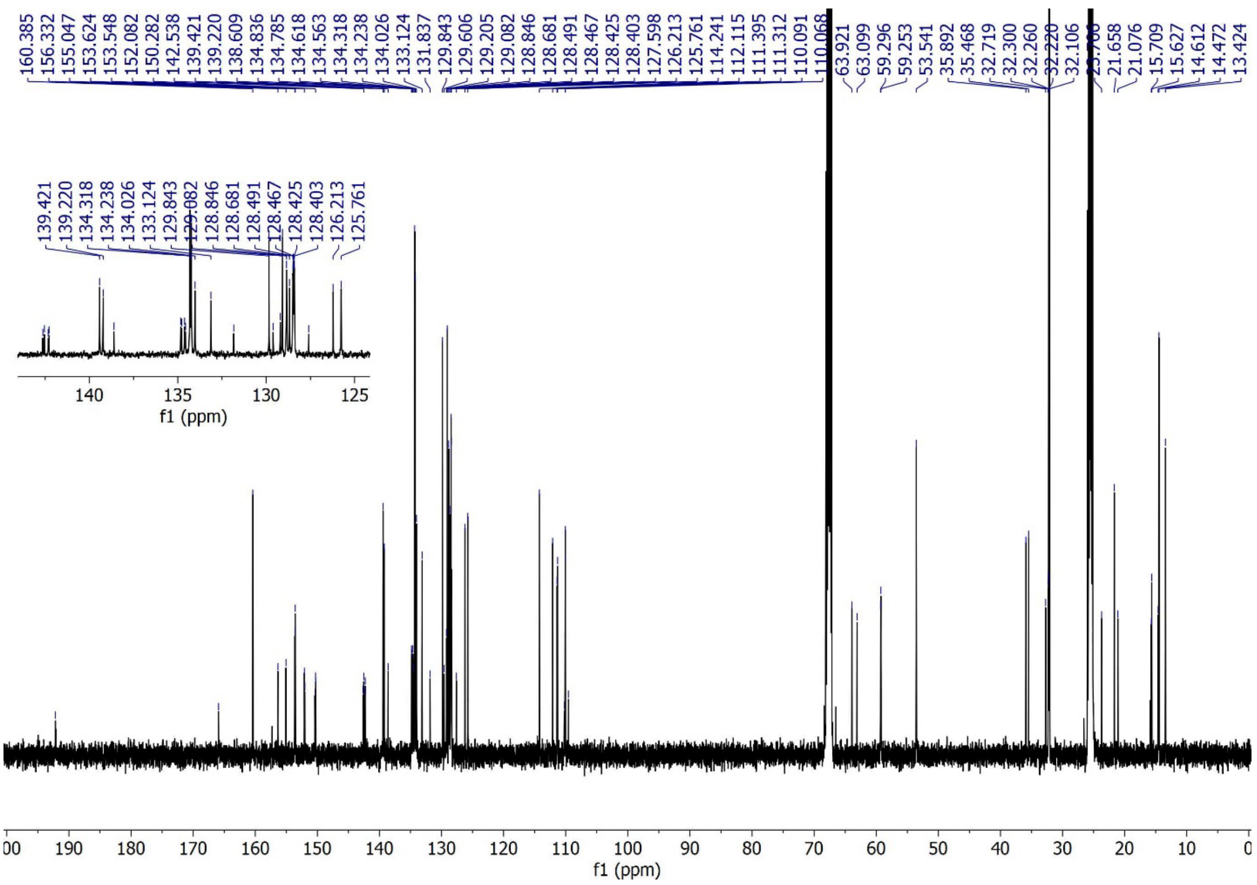

Figure S2. $^{13}C\{^{1}H\}$ NMR spectrum of a 55:45 mixture of $\mathbf{1}^{\mathbf{Me,OEt}}$ and $\mathbf{1}^{\mathbf{OEt,Me}}$ in THF-d_8 (298 K).

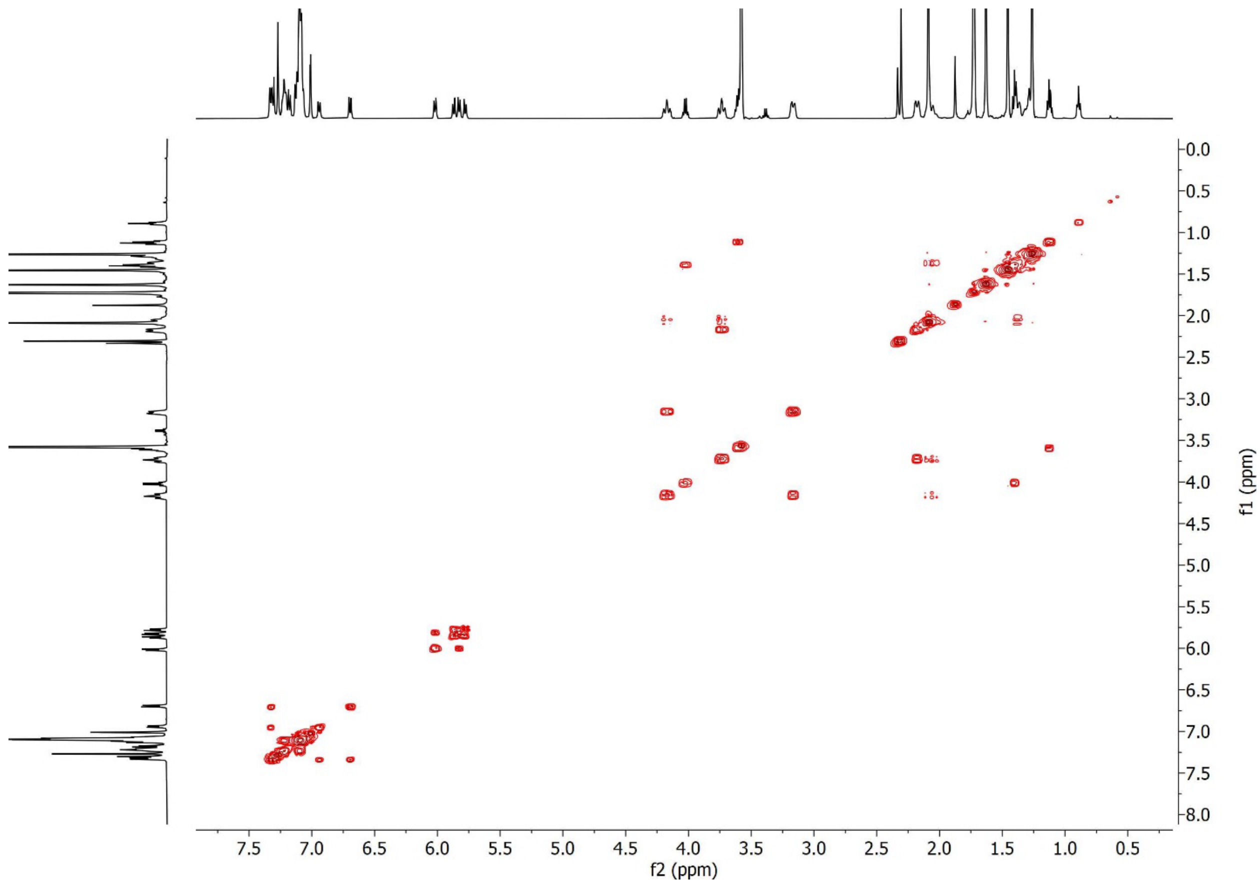

Figure S3. ^{1}H-^{1}H COSY NMR spectrum of a 55:45 mixture of $\mathbf{1}^{\mathbf{Me,OEt}}$ and $\mathbf{1}^{\mathbf{OEt,Me}}$ in THF-d_8 (298 K).

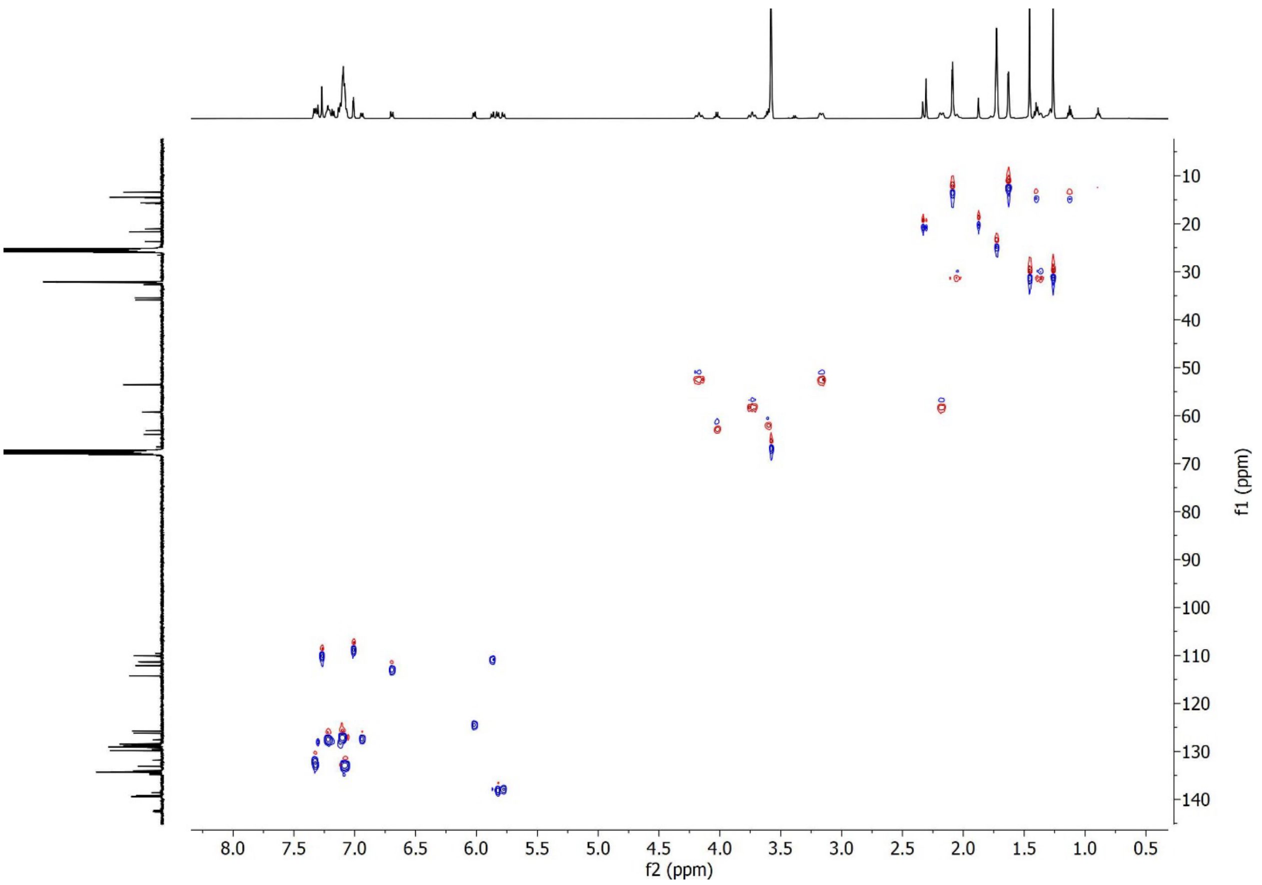

Figure S4. ^{1}H-$^{13}C\{^{1}H\}$ HSQC NMR spectrum of a 55:45 mixture of $\mathbf{1}^{\mathbf{Me,OEt}}$ and $\mathbf{1}^{\mathbf{OEt,Me}}$ in THF-d_8 (298 K).

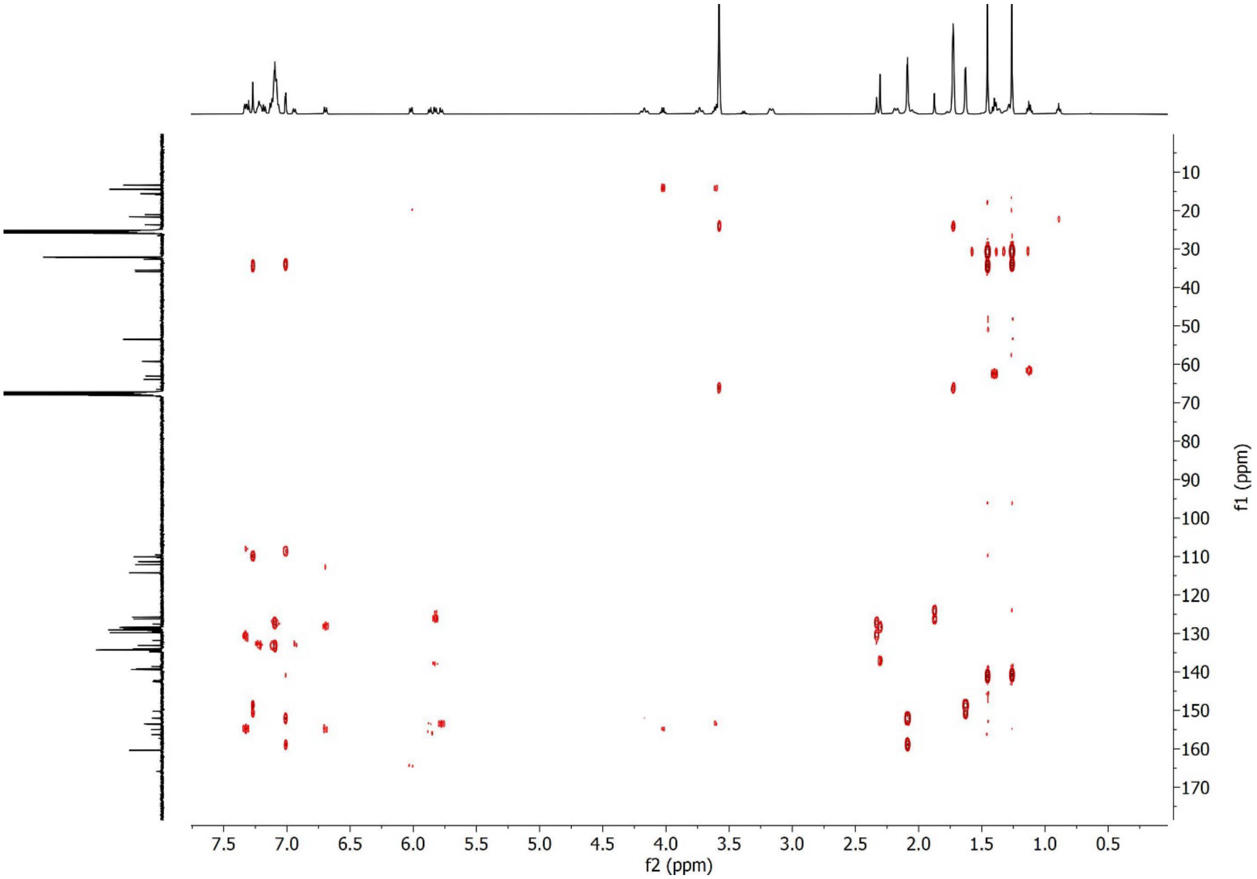

Figure S5. ^{1}H-$^{13}C\{^{1}H\}$ HMBC NMR spectrum of a 55:45 mixture of $\mathbf{1}^{\mathbf{Me,OEt}}$ and $\mathbf{1}^{\mathbf{OEt,Me}}$ in THF-d_8 (298 K).

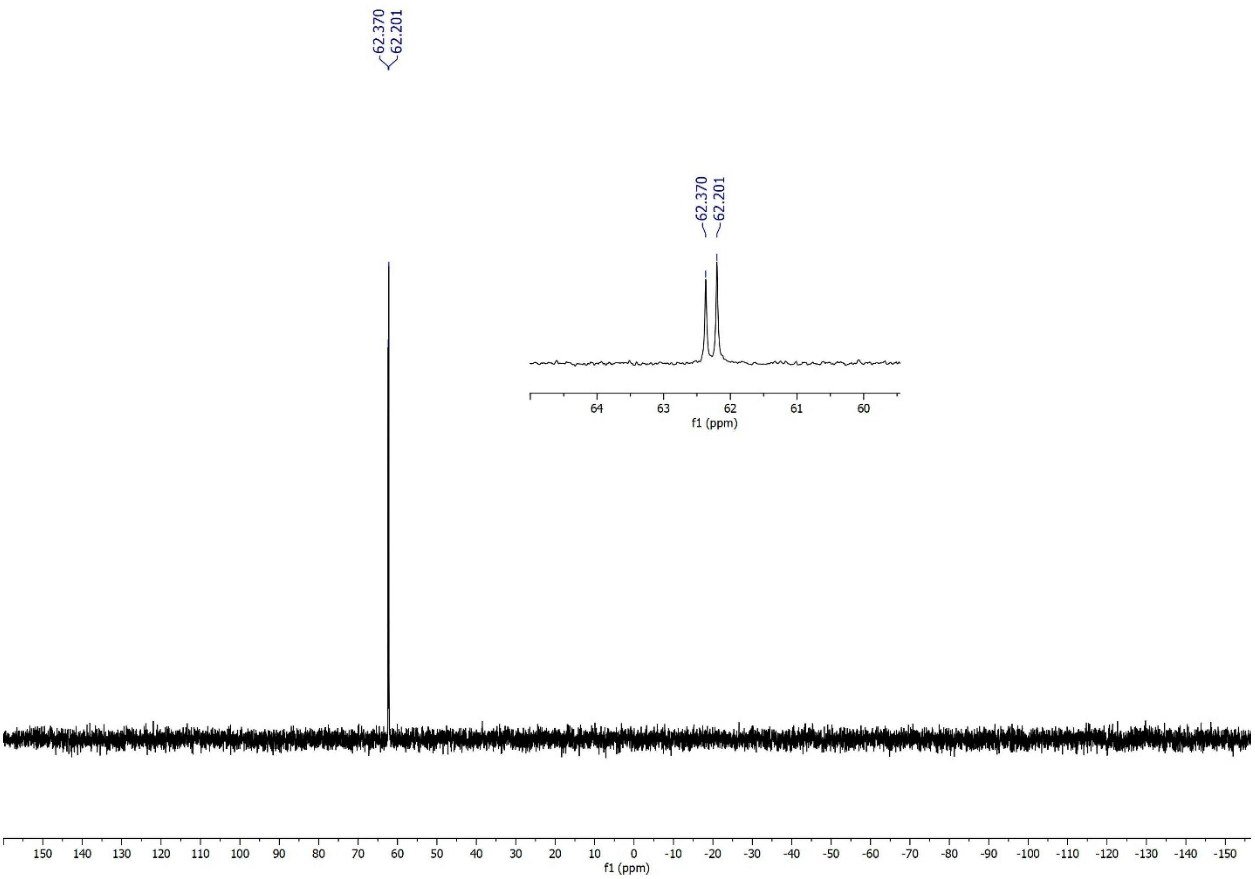

Figure S6. $^{31}P\{^{1}H\}$ NMR spectrum of a 55:45 mixture of $\mathbf{1}^{\mathbf{Me,OEt}}$ and $\mathbf{1}^{\mathbf{OEt,Me}}$ in THF-d_8 (298 K).

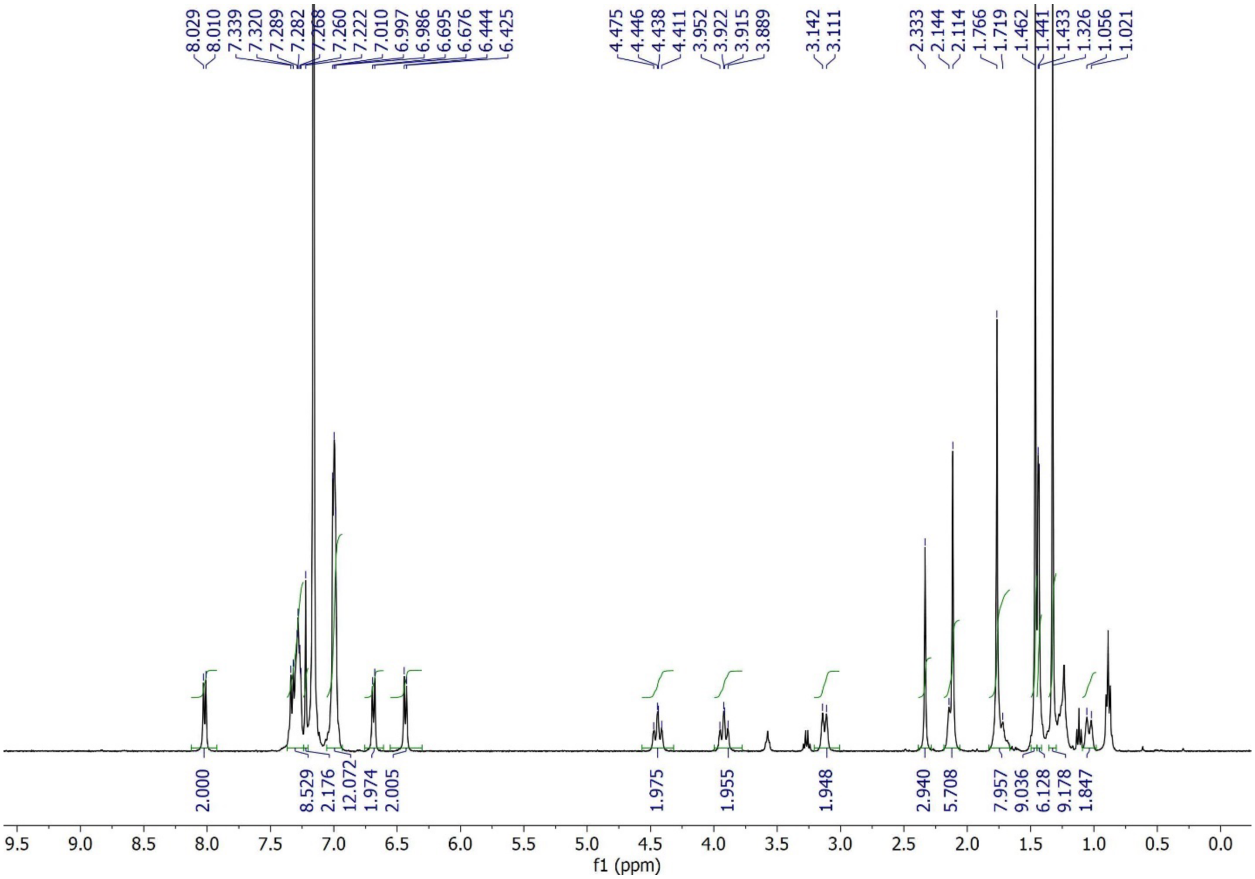

Figure S7. ^{1}H NMR spectrum of $\mathbf{1}^{\mathbf{Me,Me}}$ in C_6D_6 (298 K).

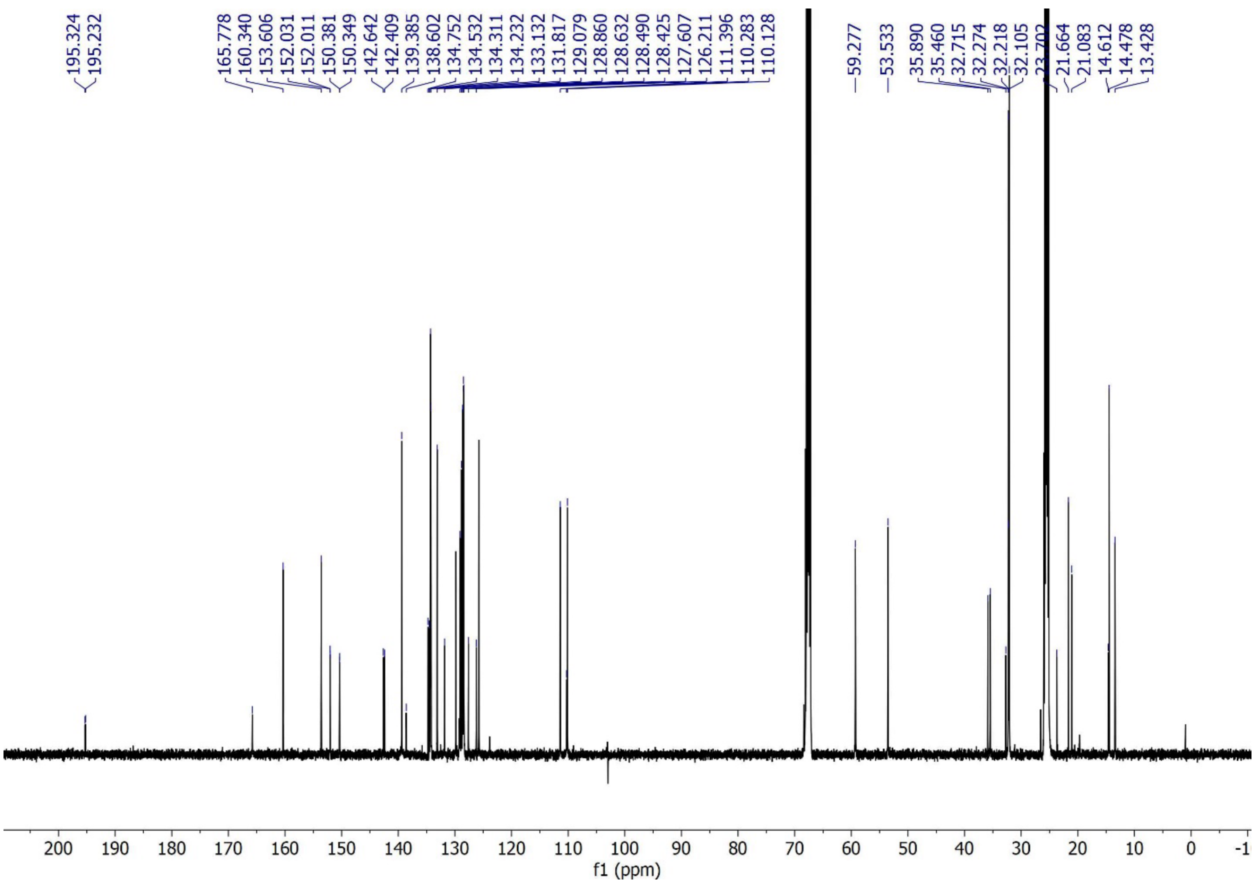

Figure S8. $^{13}C\{^{1}H\}$ NMR spectrum of $\mathbf{1}^{\mathbf{Me,Me}}$ in THF-d_8 (298 K).

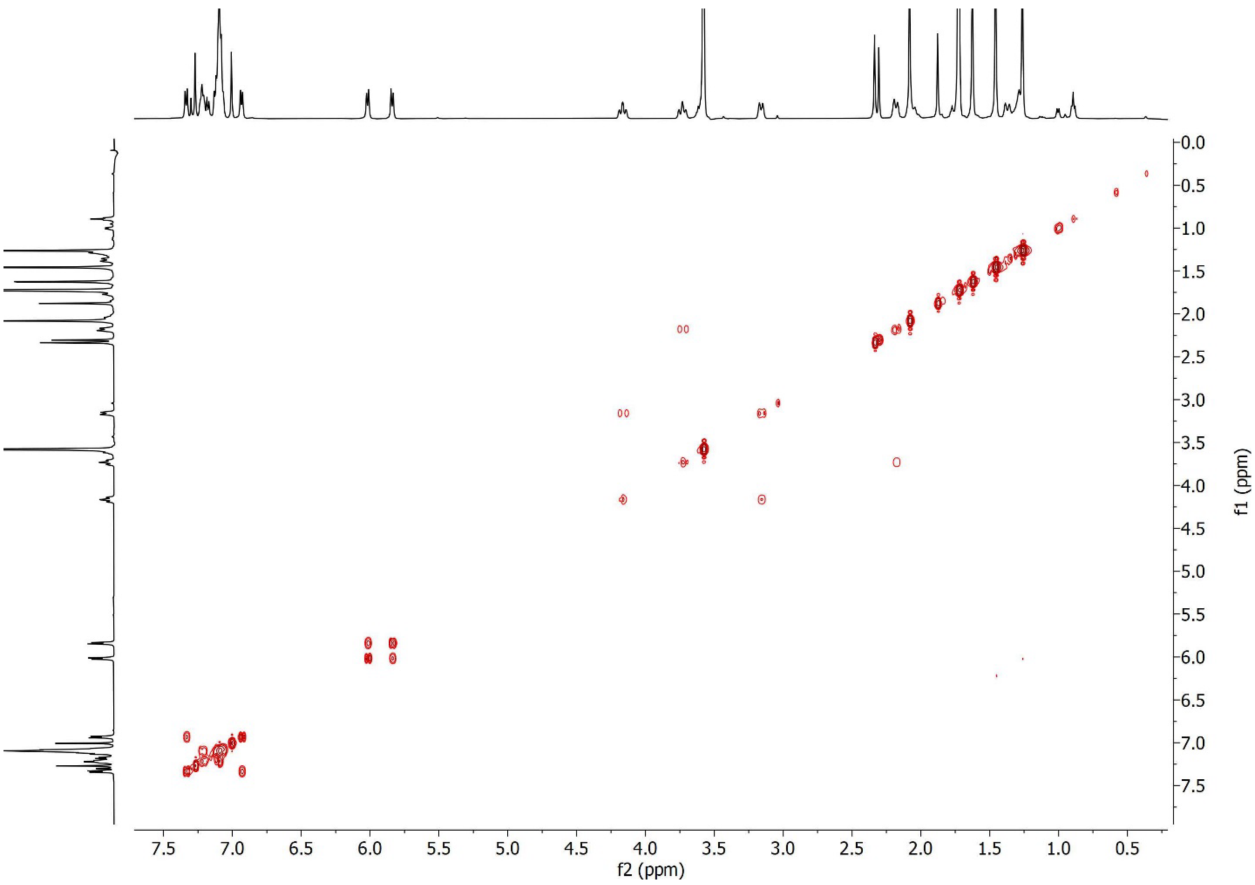

Figure S9. ^{1}H-^{1}H COSY NMR spectrum of **1**$^{\mathbf{Me,Me}}$ in THF-d_8 (298 K).

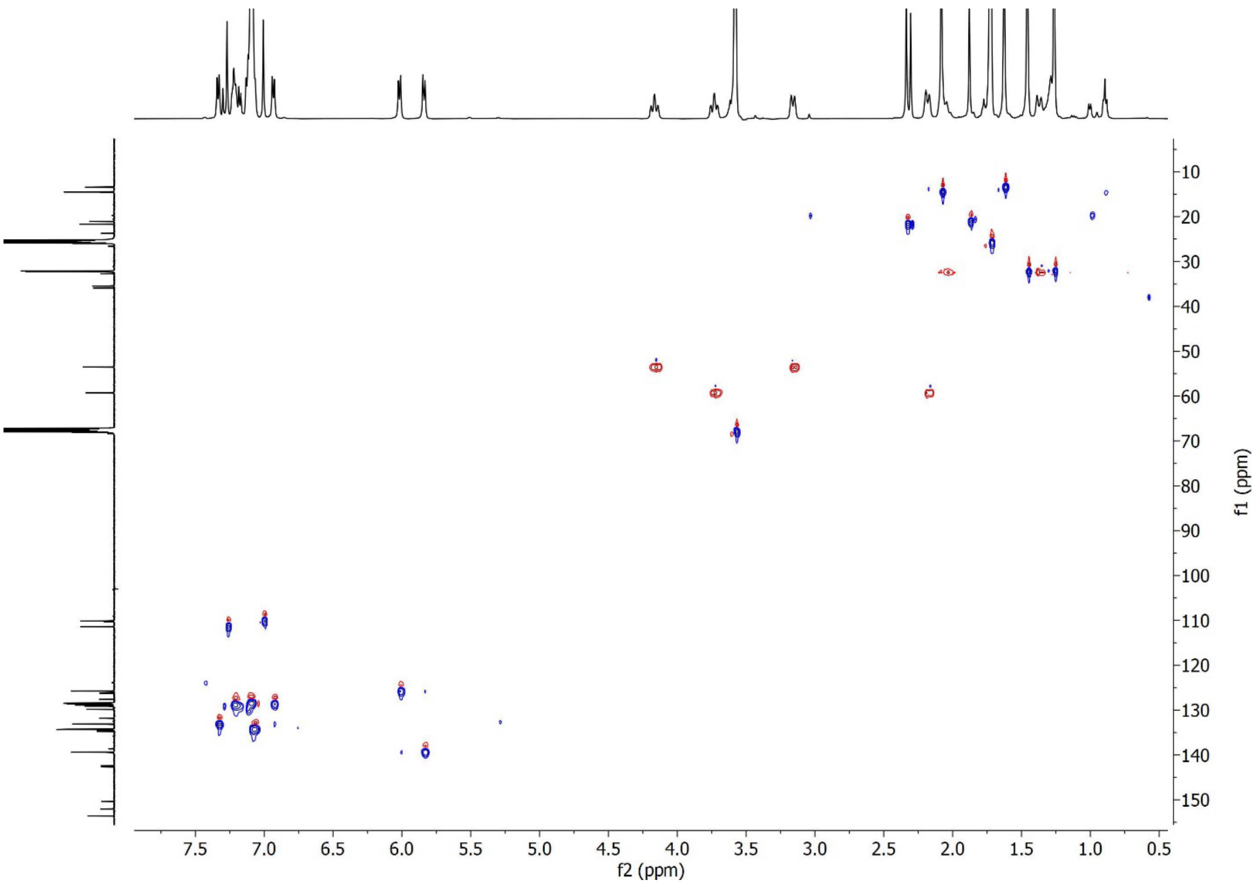

Figure S10. ^{1}H-$^{13}C\{^{1}H\}$ HSQC spectrum of $\mathbf{1^{Me,Me}}$ in THF-d_8 (298 K).

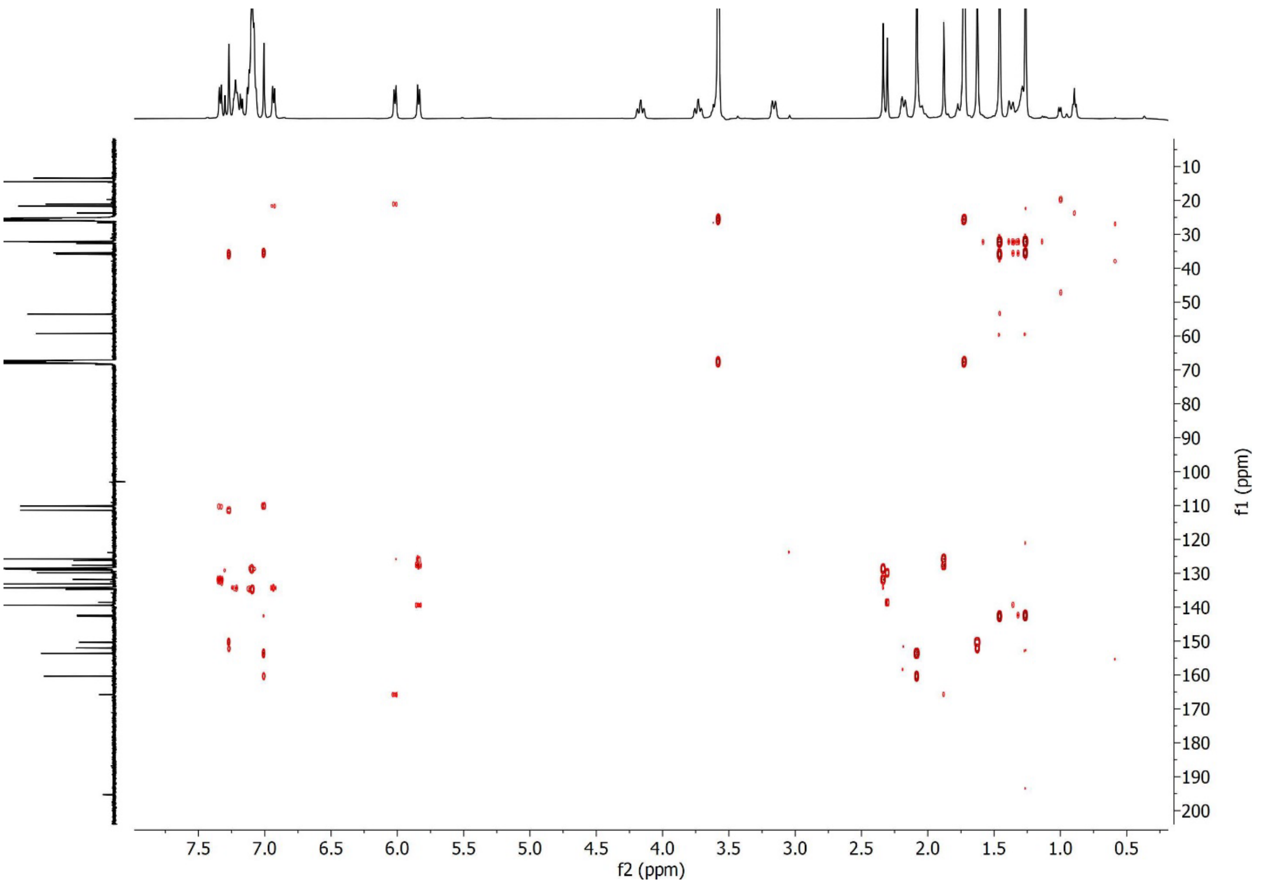

Figure S11. ^{1}H-$^{13}C\{^{1}H\}$ HMBC NMR spectrum of $\mathbf{1}^{\mathbf{Me,Me}}$ in THF-d_8 (298 K).

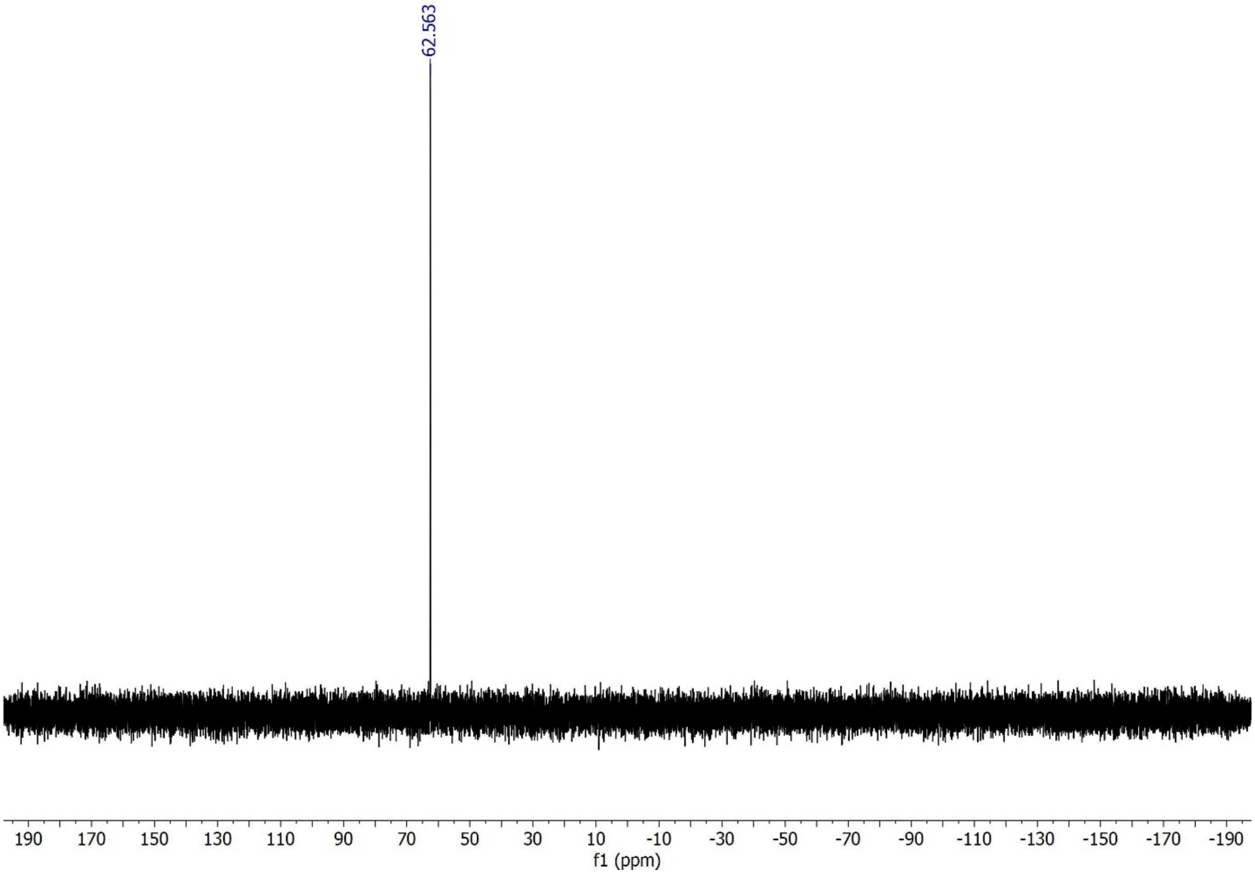

Figure S12. $^{31}P\{^{1}H\}$ NMR spectrum of $\mathbf{1}^{\mathbf{Me,Me}}$ in THF-d_8 (298 K).

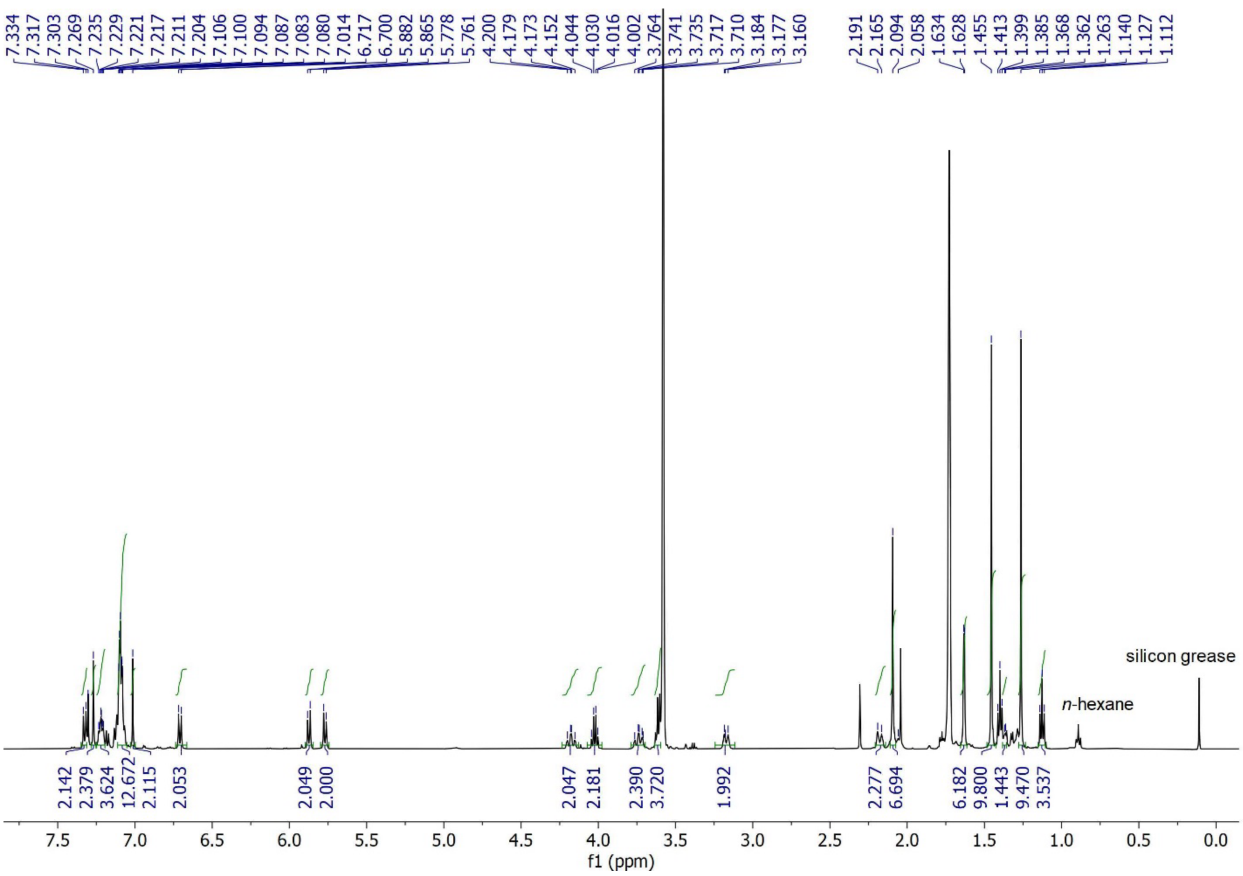

Figure S13. ^{1}H NMR spectrum of $\mathbf{1}^{\mathbf{OEt,OEt}}$ in THF-d_8 (298 K).

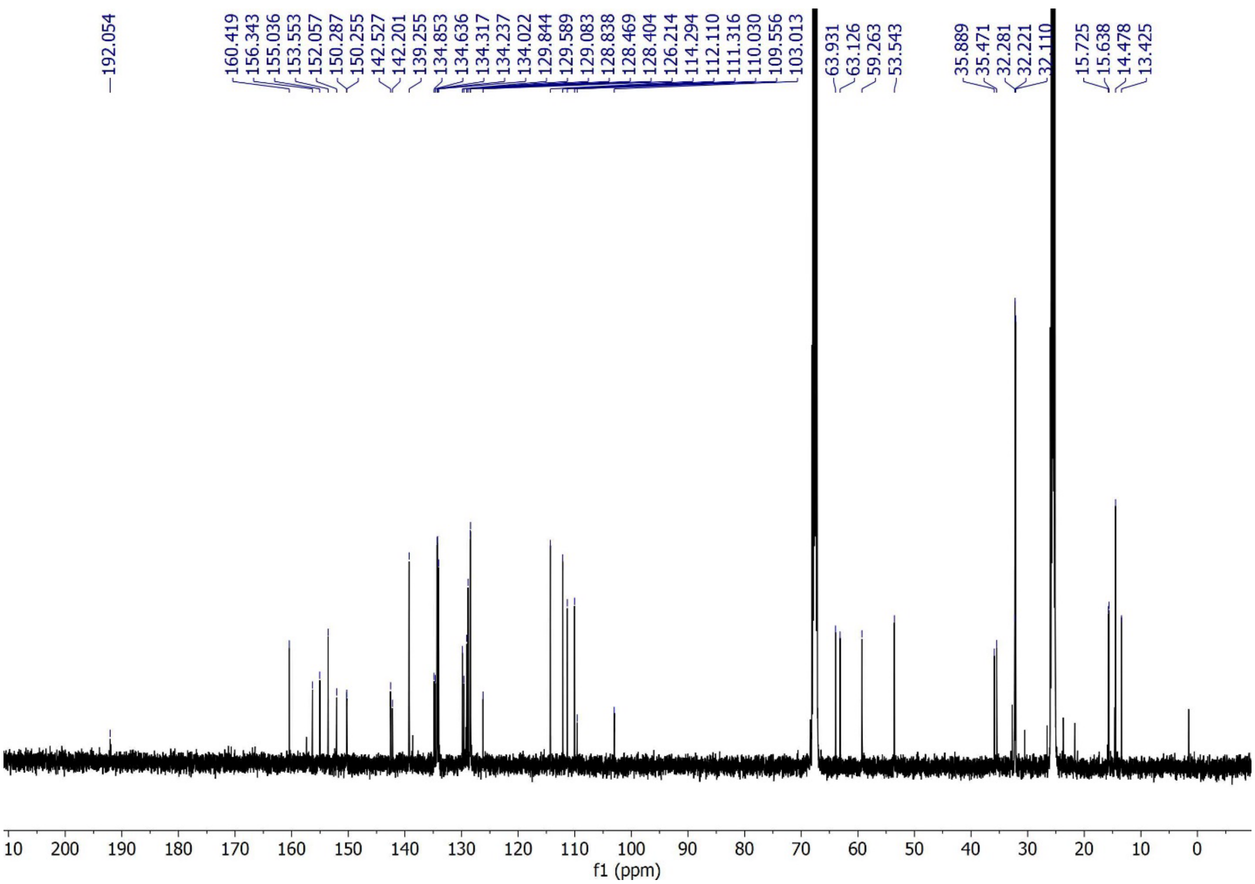

Figure S14. $^{13}C\{^{1}H\}$ NMR spectrum of $\mathbf{1}^{\mathbf{OEt,OEt}}$ in THF-d_8 (298 K).

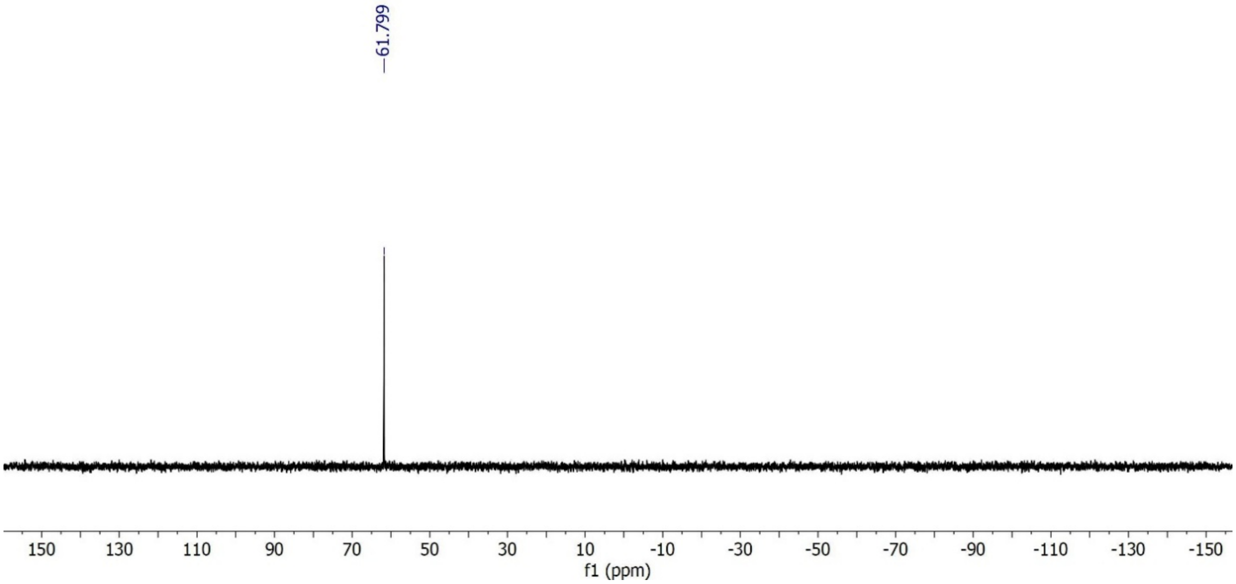

Figure S15. $^{31}P\{^{1}H\}$ NMR spectrum of $\mathbf{1}^{\mathbf{OEt,OEt}}$ in THF-d_8 (298 K).

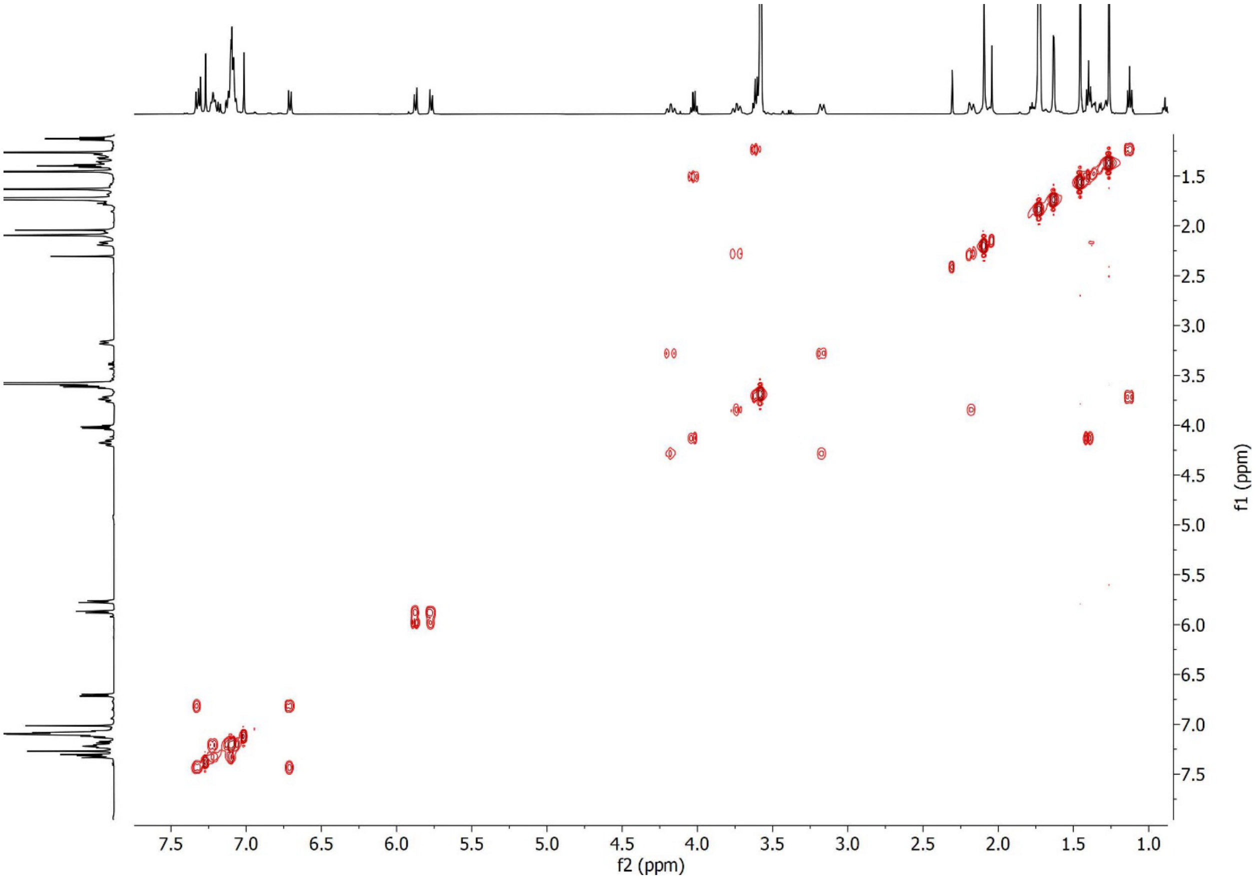

Figure S16. ^{1}H-^{1}H COSY NMR spectrum of $\mathbf{1^{OEt,OEt}}$ in THF-d_8 (298 K).

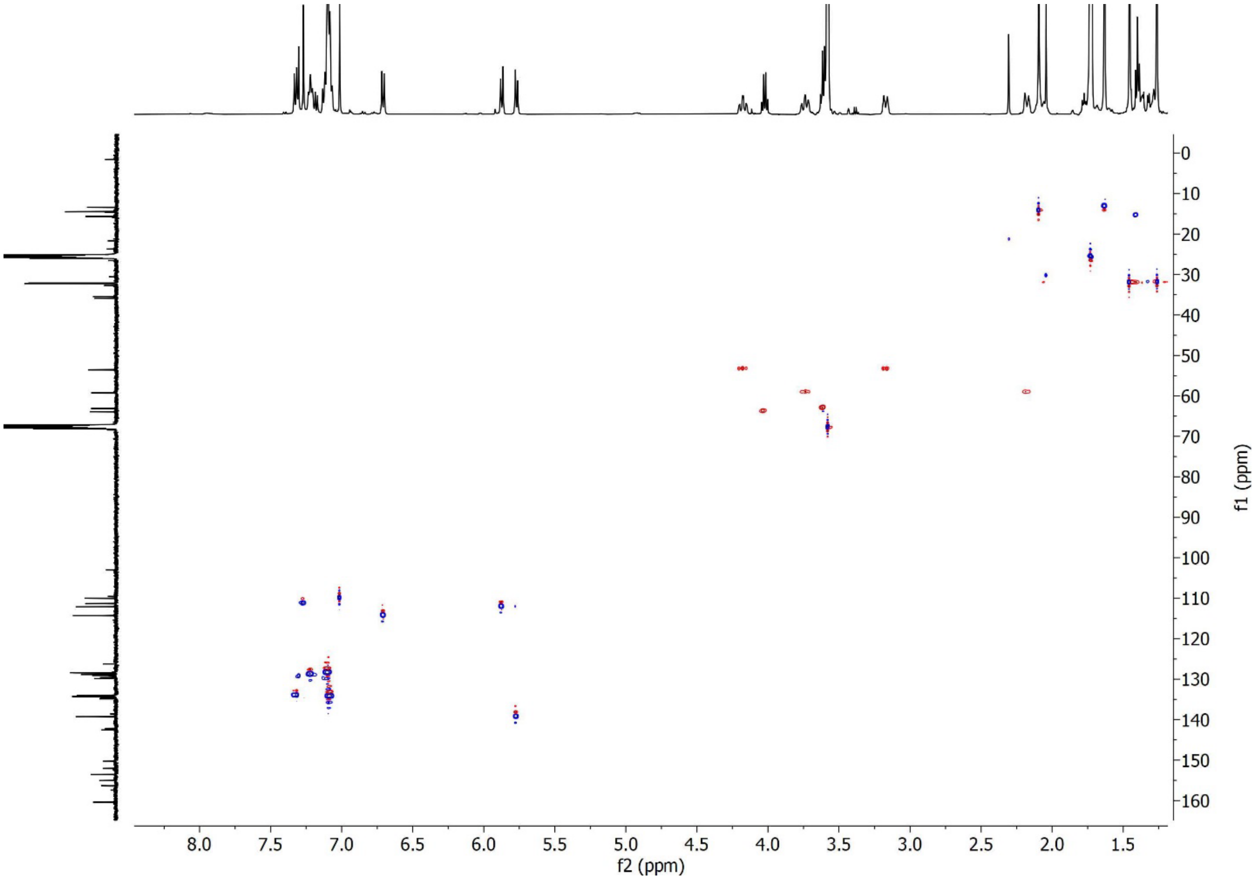

Figure S17. ^{1}H-^{13}C{^{1}H} HSQC NMR spectrum of **1^{OEt,OEt}** in THF-d_8 (298 K).

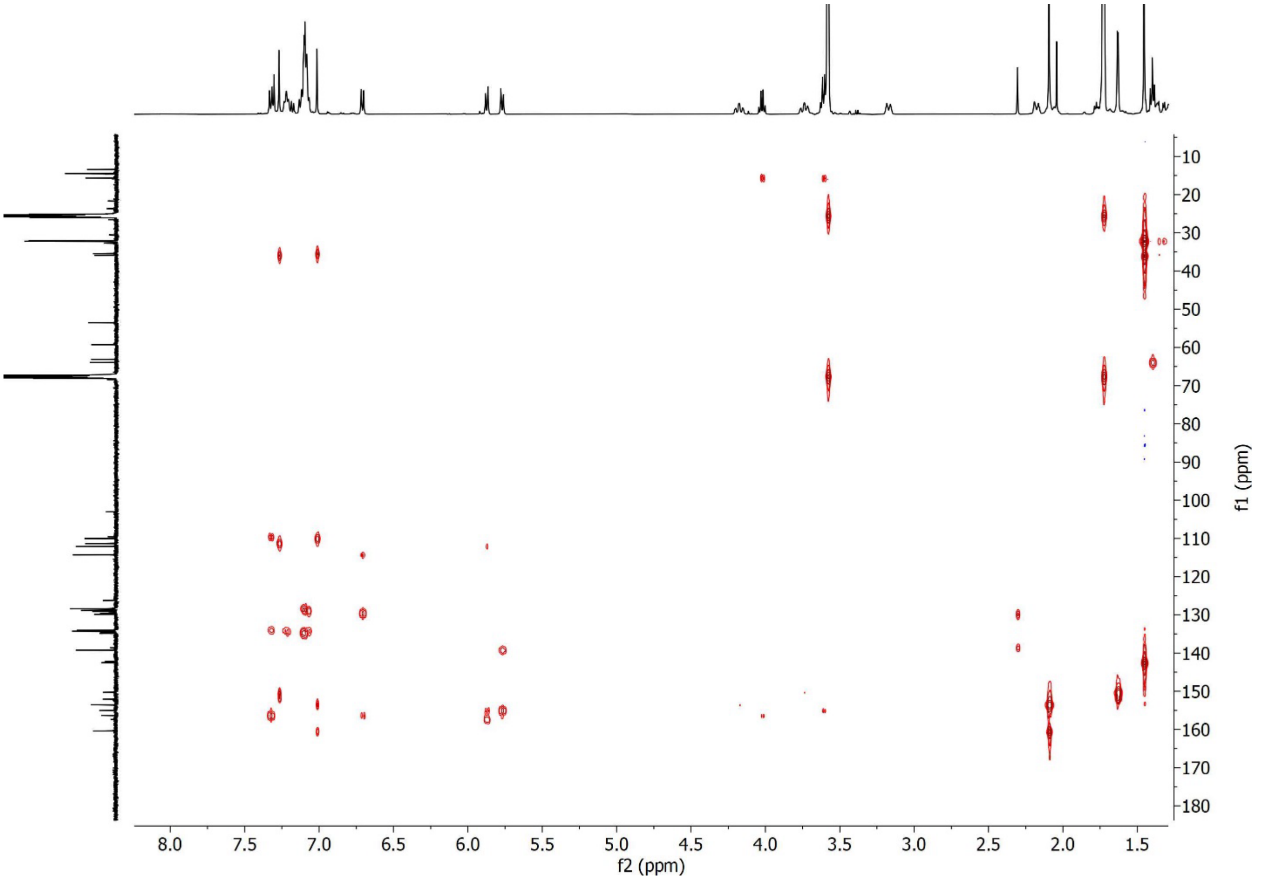

Figure S18. ^{1}H-$^{13}C\{^{1}H\}$ HMBC NMR spectrum of $\mathbf{1}^{\mathbf{OEt,OEt}}$ in THF-d_8 (298 K).

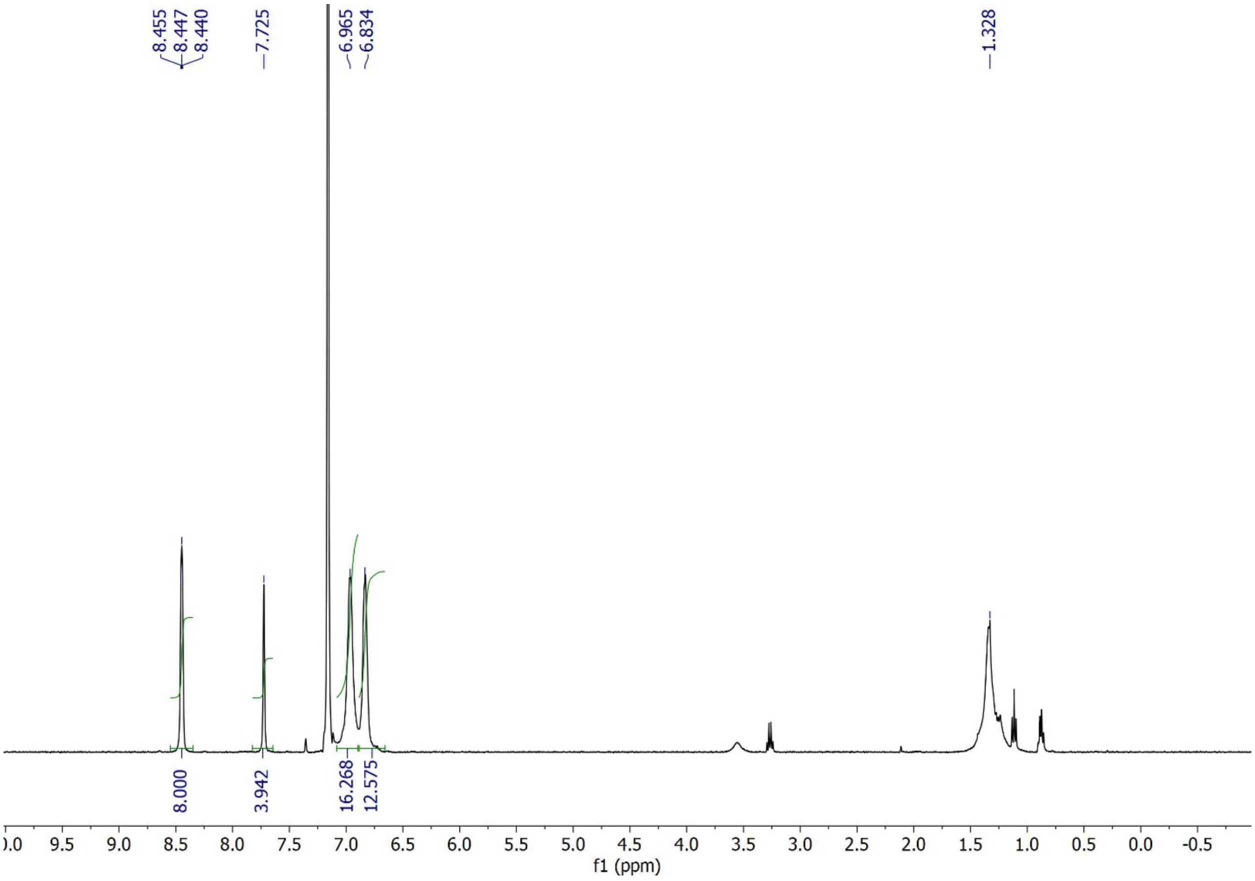

Figure S19. ^{1}H NMR spectrum of **[3][BAr^{F}$_4$]** in C_6D_6 (298 K).

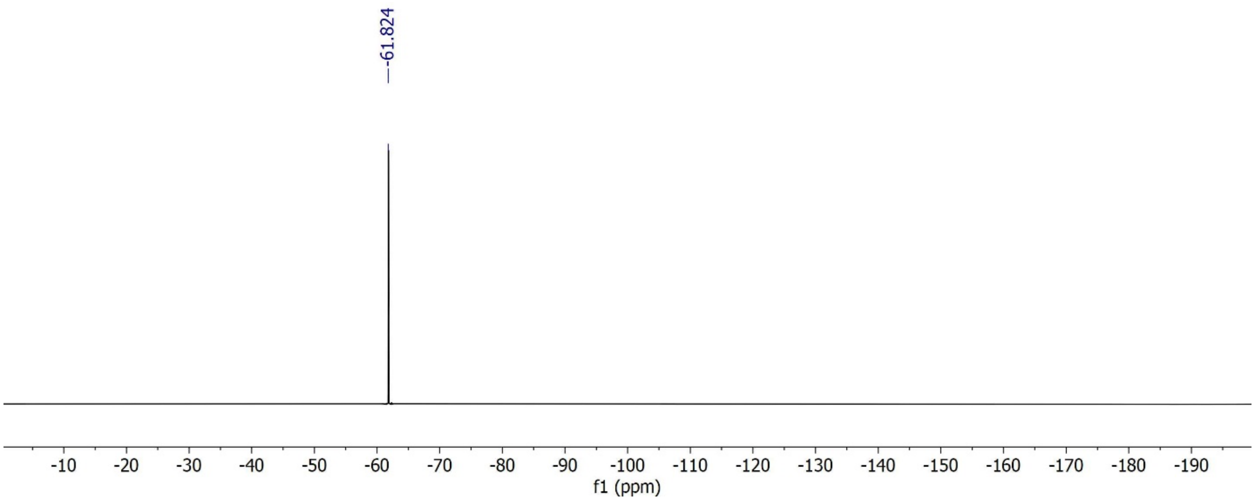

Figure S20. ^{19}F{^{1}H} NMR spectrum of **[3][BAr^{F}$_4$]** in C_6D_6 (298 K).

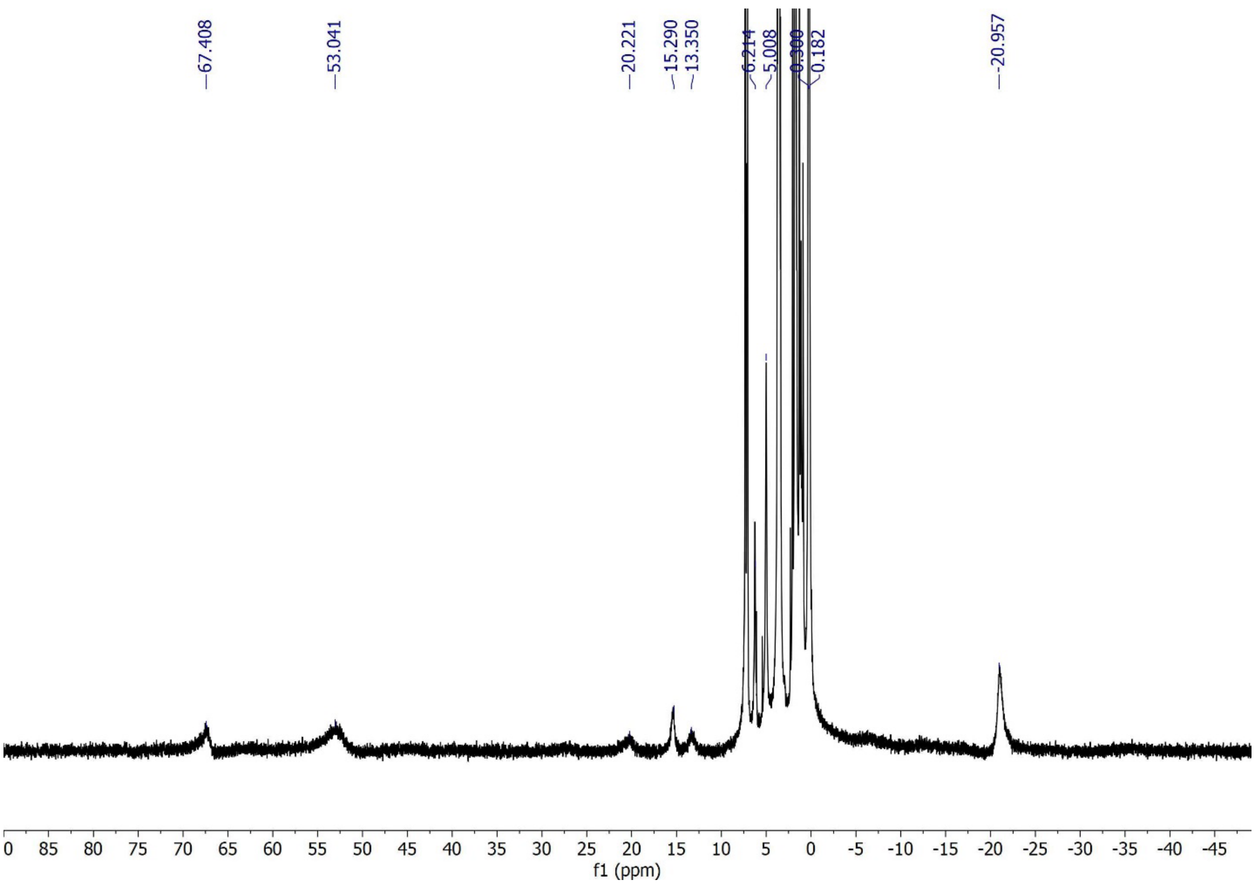

Figure S21. ^{1}H NMR spectrum of **[4][PF$_6$]$_2$** in THF-d_8 (298 K).

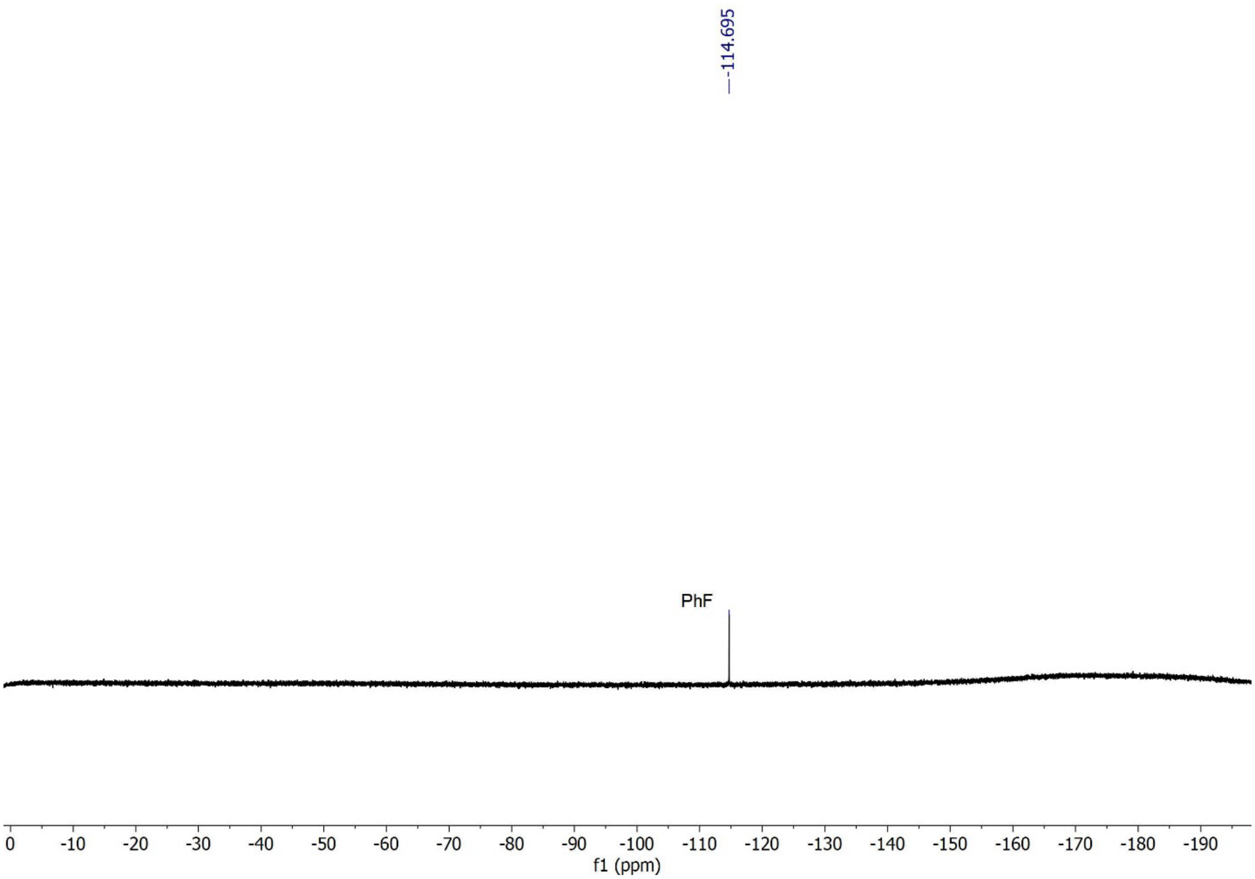

Figure S22. $^{19}F\{^{1}H\}$ NMR spectrum of **[4][PF$_6$]$_2$** in THF-d_8 (298 K).

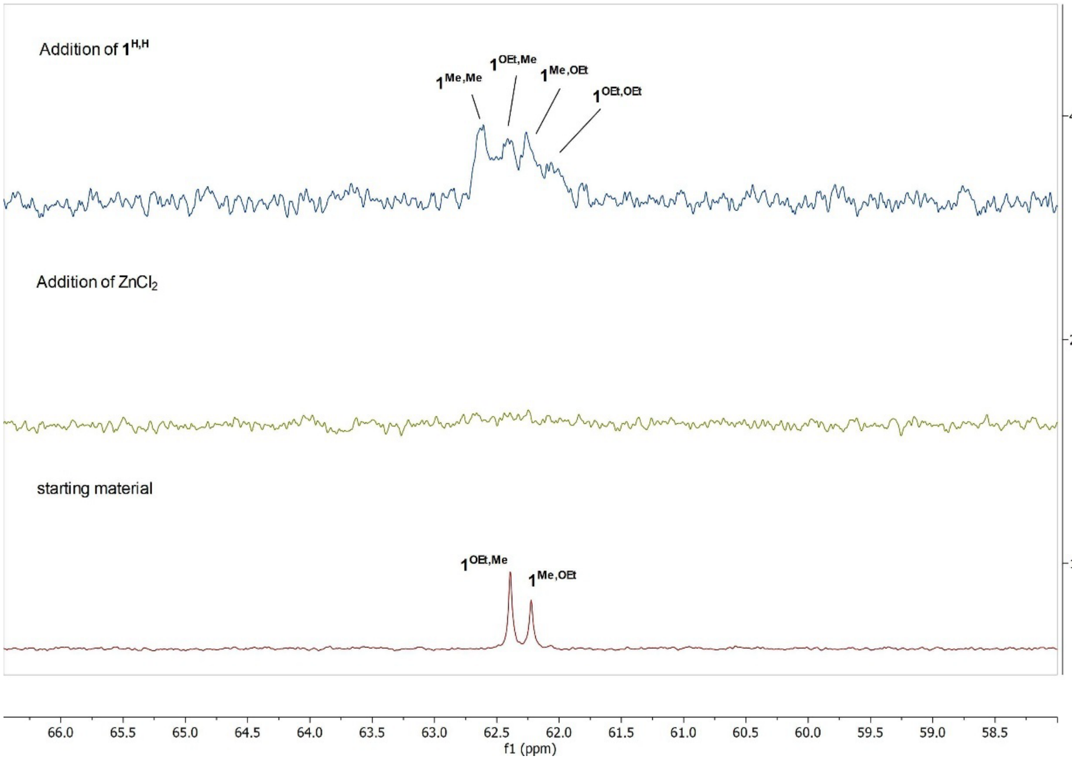

Figure S23. $^{31}P\{^1H\}$ NMR spectrum of the reaction of mixture of $\mathbf{1}^{\mathbf{OEt,Me}}$ and $\mathbf{1}^{\mathbf{Me,OEt}}$ with $ZnCl_2$, followed by addition of $\mathbf{1}^{\mathbf{H,H}}$ in THF-d_8 (298 K), showing the formation of an equilibrium mixture.

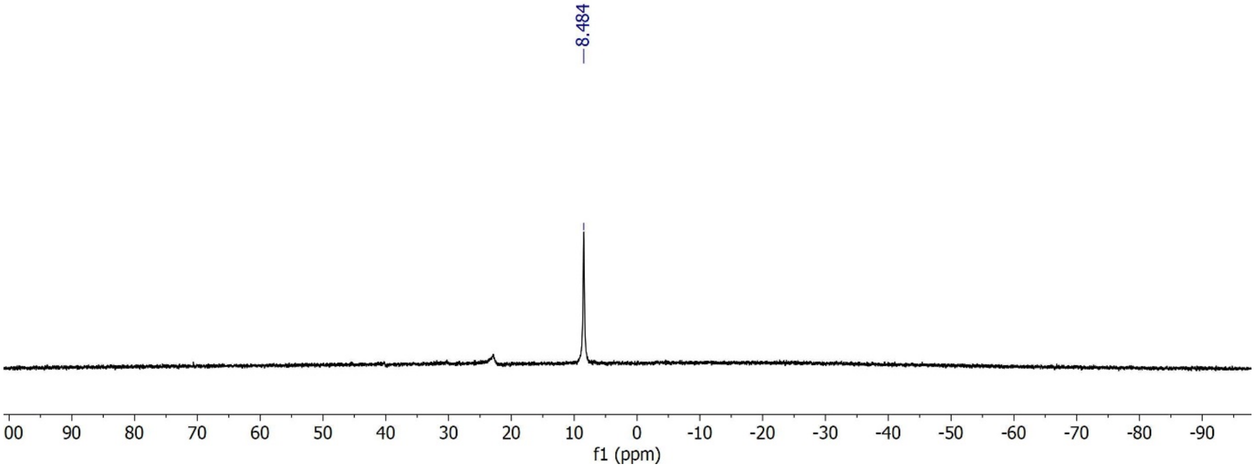

Figure S24. $^{11}B\{^1H\}$ NMR spectrum of reaction mixture of $\mathbf{1}^{\mathbf{H,H}}$ and BCl_3 in THF-d_8 (128 MHz, 298 K).

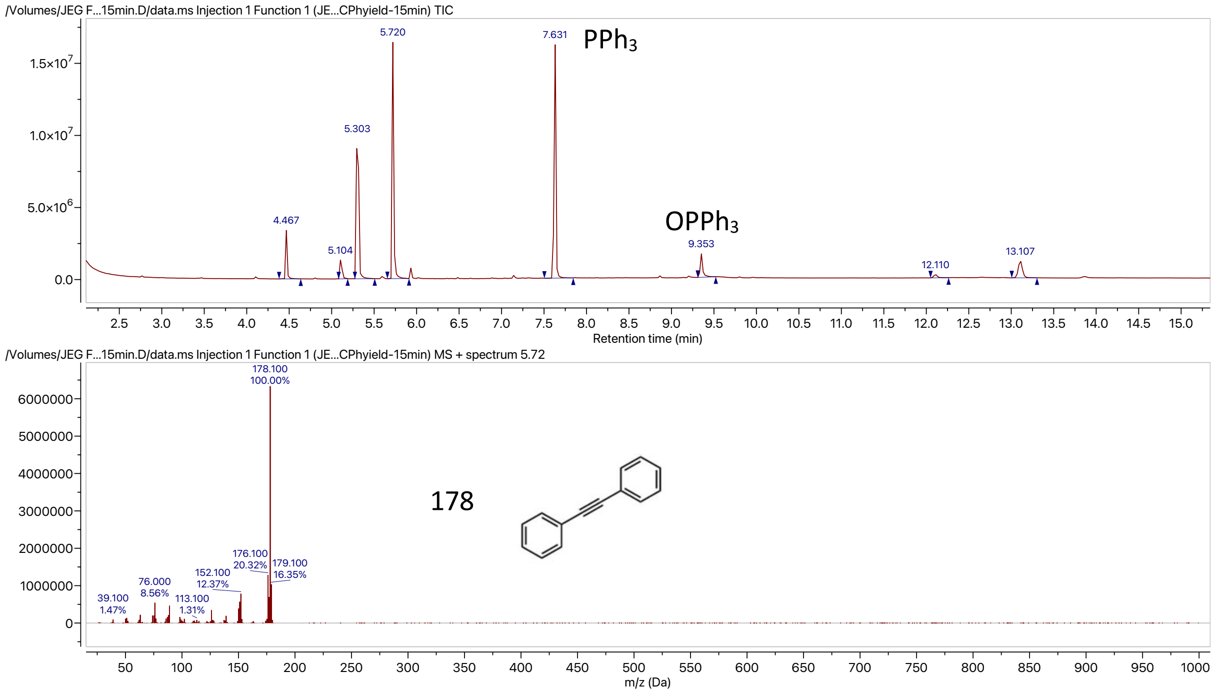

Figure S25. GC-MS data for the chemical oxidation of $\mathbf{1^{H,H}}$ with 3 equiv of AgOTf.

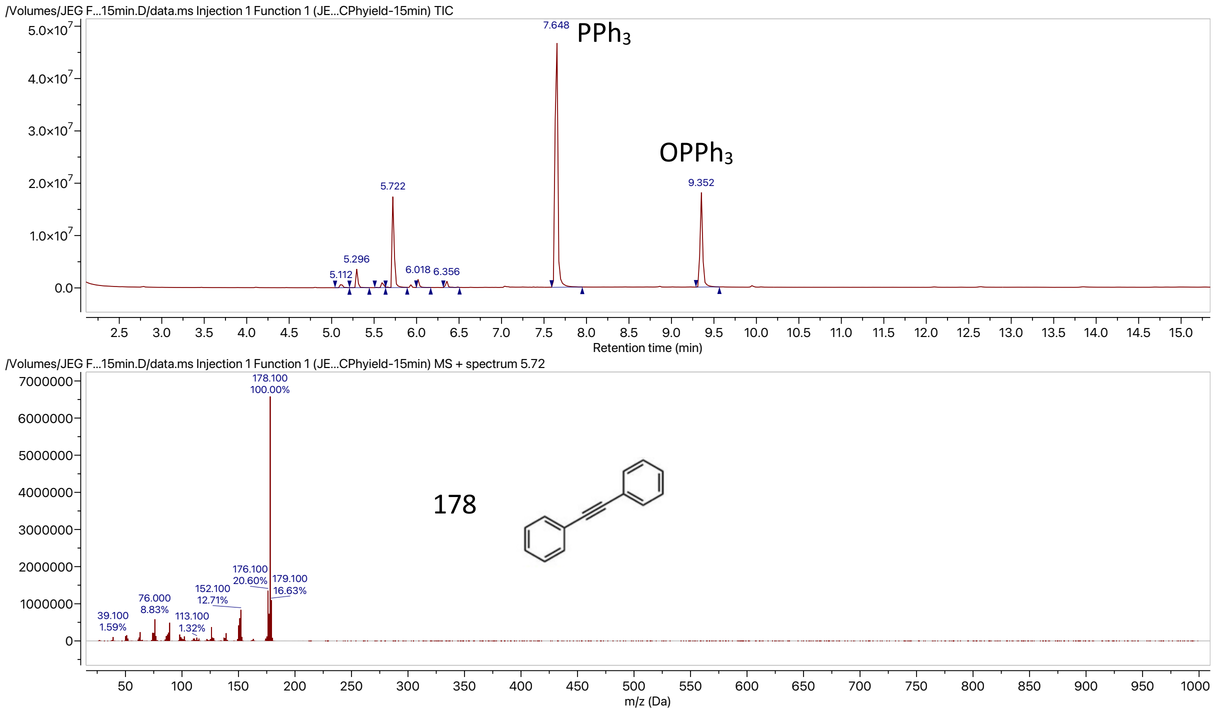

Figure S26. GC-MS data for the chemical oxidation of $\mathbf{1^{H,H}}$ with 1 atm of O_2.

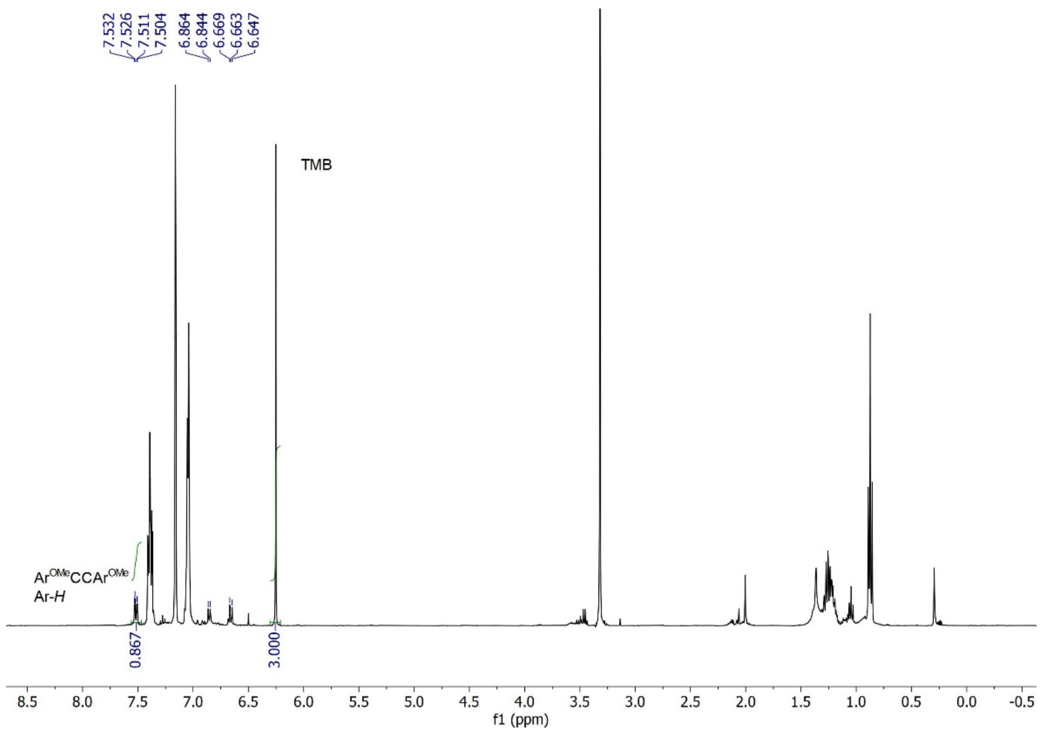

Figure S27. ^{1}H NMR spectrum of the Et_2O extraction of reaction of $\mathbf{1}^{\mathbf{OMe,OMe}}$ with 3 equiv of AgOTf (C_6D_6, 400 MHz, 298 K). 1,3,5-trimethoxybenzene (TMB) was used as an internal standard.

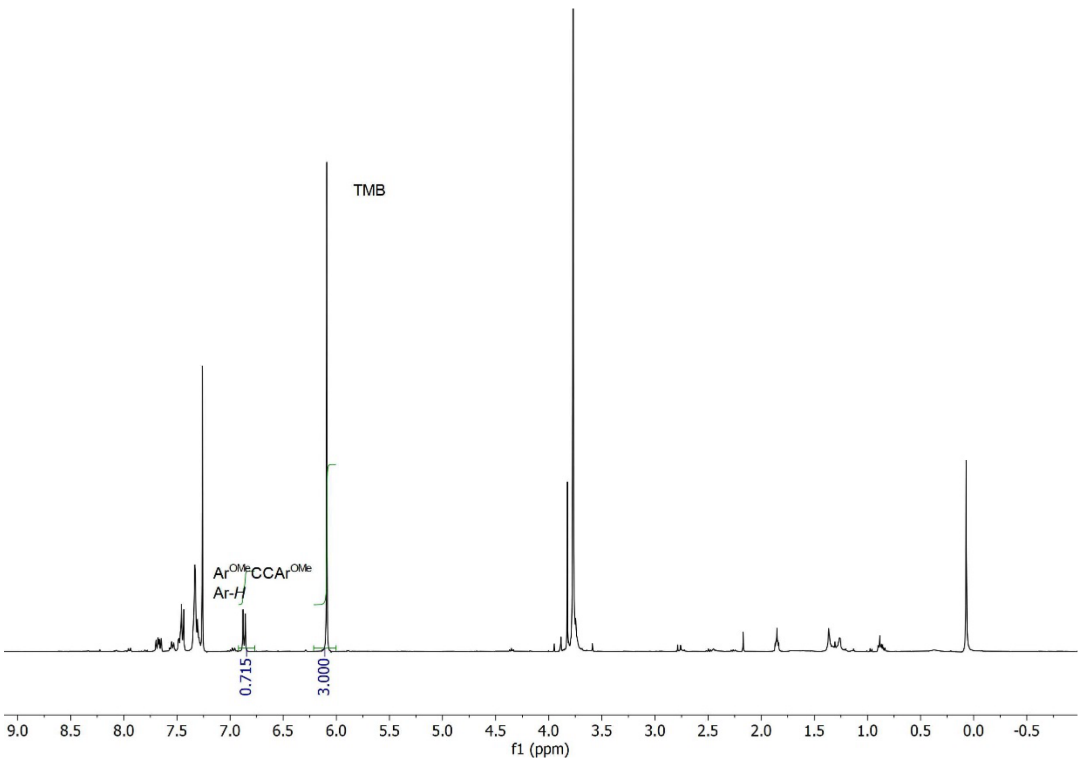

Figure S28. ^{1}H NMR spectrum of the reaction of $\mathbf{1}^{\mathbf{OMe,OMe}}$ with 1 atm of O_2 after workup ($CDCl_3$, 400 MHz, 298 K). 1,3,5-trimethoxybenzene (TMB) was used as internal standard.

Stepwise C–C σ-bond Metathesis and GC-MS Spectroscopy Data

General Procedure: To a stirred solution of **[Fe_2N_2]** (23.5 mg, 0.020 mmol) in a 20 mL vial with a septum-sealed screw cap was added PhCCPh (1.8 mg, 0.010 mmol) and Ar^XCCAr^X (0.010 mmol) at the same time. The solution was then allowed to stir for 4 h, resulting in gradual color change from red to green. A THF solution of $ZnCl_2$ was then added with a microsyringe, and the resulting mixture was stirred for 1 h. The seal between the vial and the screw cap was reinforced by tightly taping around the joint with electrical tape. The vial was then removed from the glovebox, and the headspace within the vial was purged via a needle with O_2 for 5 min. The O_2 purge resulted in a rapid color change to brown and a precipitate formed. After stirring for 30 min, the vial was opened to air, and the mixture was filtered through Celite, concentrated with a flow of air to *ca.* 1.5 mL, filtered through Celite again, and subjected to GC-MS analysis. The GC-MS sample was then transferred into a 20 mL scintillation vial and evaporated with a flow of air to afford a pale-yellow oil. A known amount of either ferrocene or 1,3,5-trimethoxybenzene was then added as an internal standard. The mixture was then dissolved in 0.8 mL of $CDCl_3$, transferred into a NMR tube, and subjected to NMR spectroscopic analysis. Product yields were reported from either ^1H NMR spectroscopic analysis (X = 4-Me, 3-Me, 4-^{t}Bu, 4-F, 4-OMe, 4-OEt) or GC-MS measurement (X = 4-Ph, 4-Cl, 4-CF_3).

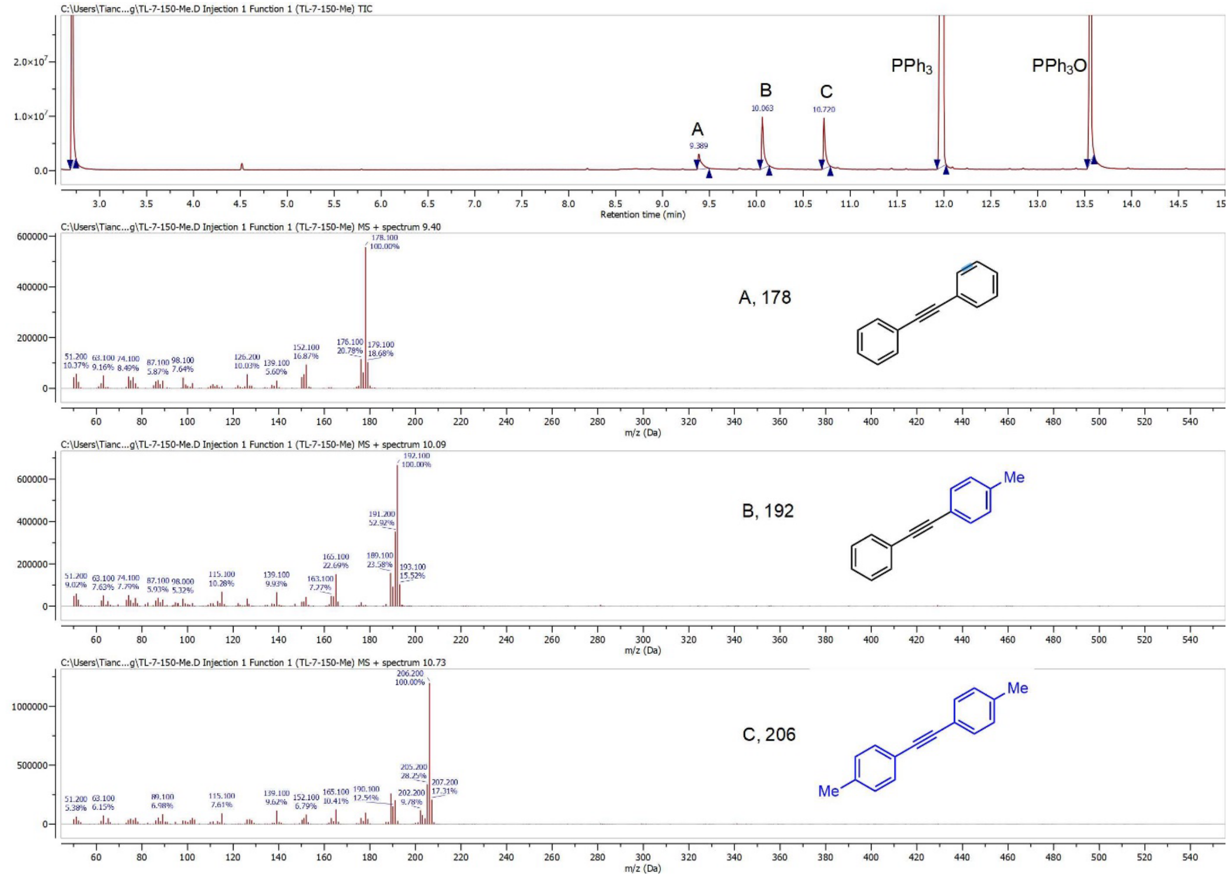

Figure S29. GC-MS data for the σ-bond metathesis of PhCCPh and $Ar^{Me}CCAr^{Me}$ (Ar^{Me} = 4-Me-Ph).

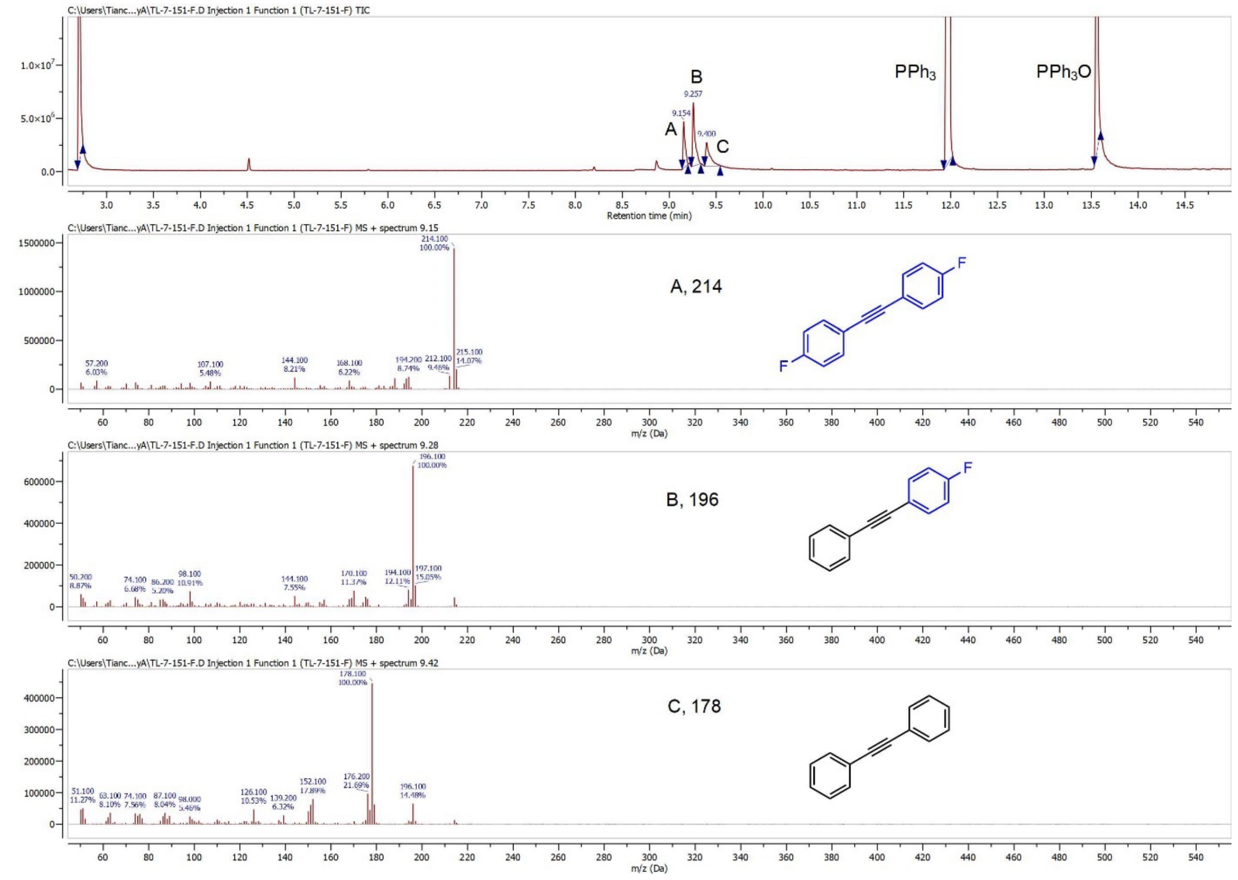

Figure S30. GC-MS data for the σ-bond metathesis of PhCCPh and $Ar^{F}CCAr^{F}$ (Ar^{F} = 4-F-Ph).

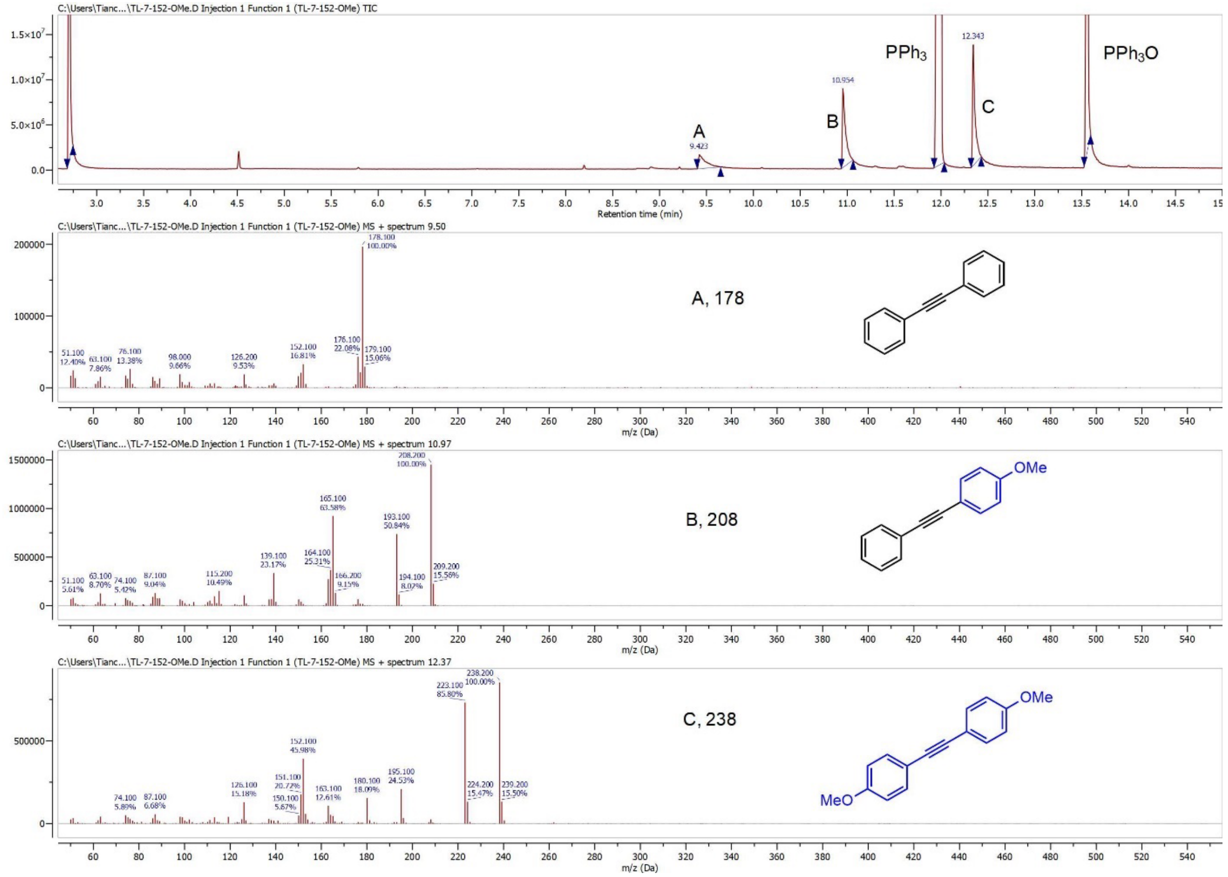

Figure S31. GC-MS data for the σ-bond metathesis of PhCCPh and $Ar^{OMe}CCAr^{OMe}$ (Ar^{OMe} = 4-OMe-Ph).

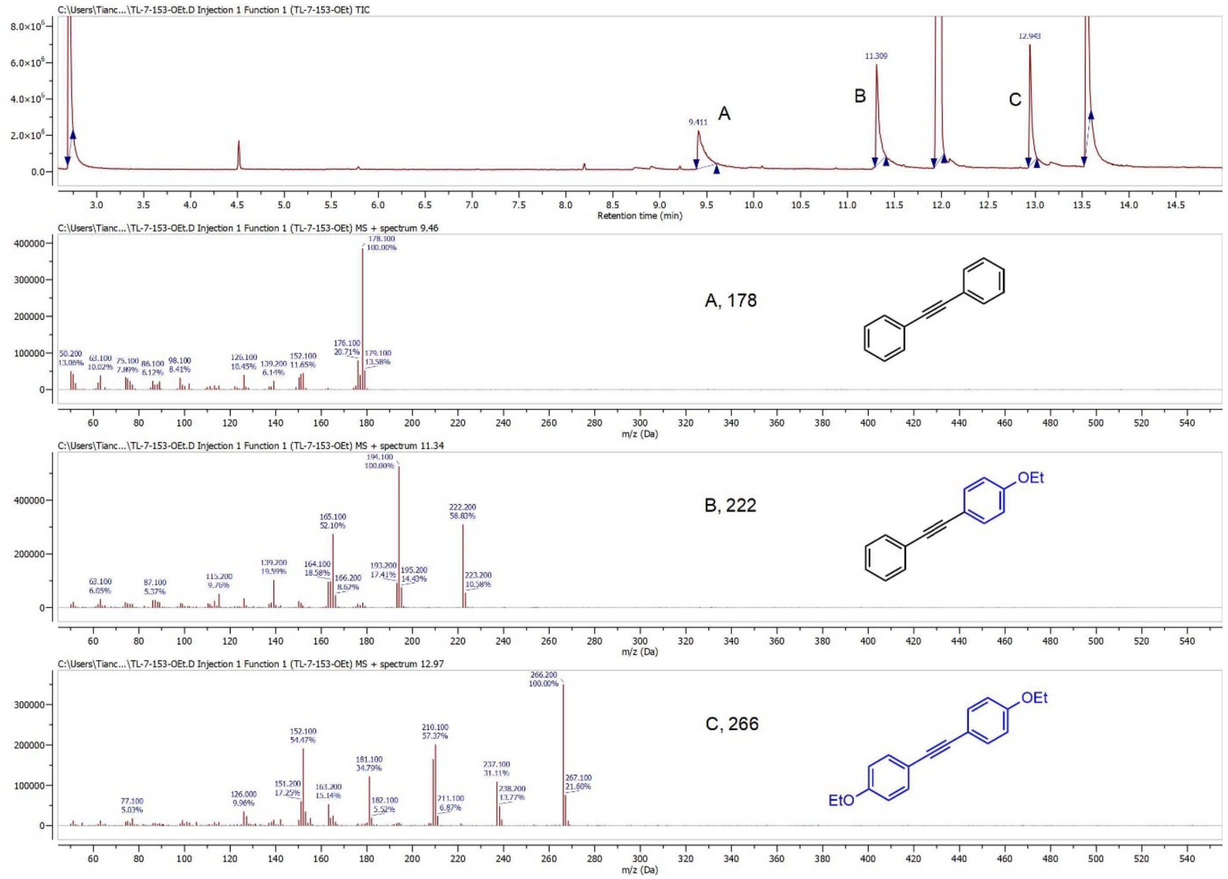

Figure S32. GC-MS data for the σ-bond metathesis of PhCCPh and $Ar^{OEt}CCAr^{OEt}$ (Ar^{OEt} = 4-OEt-Ph).

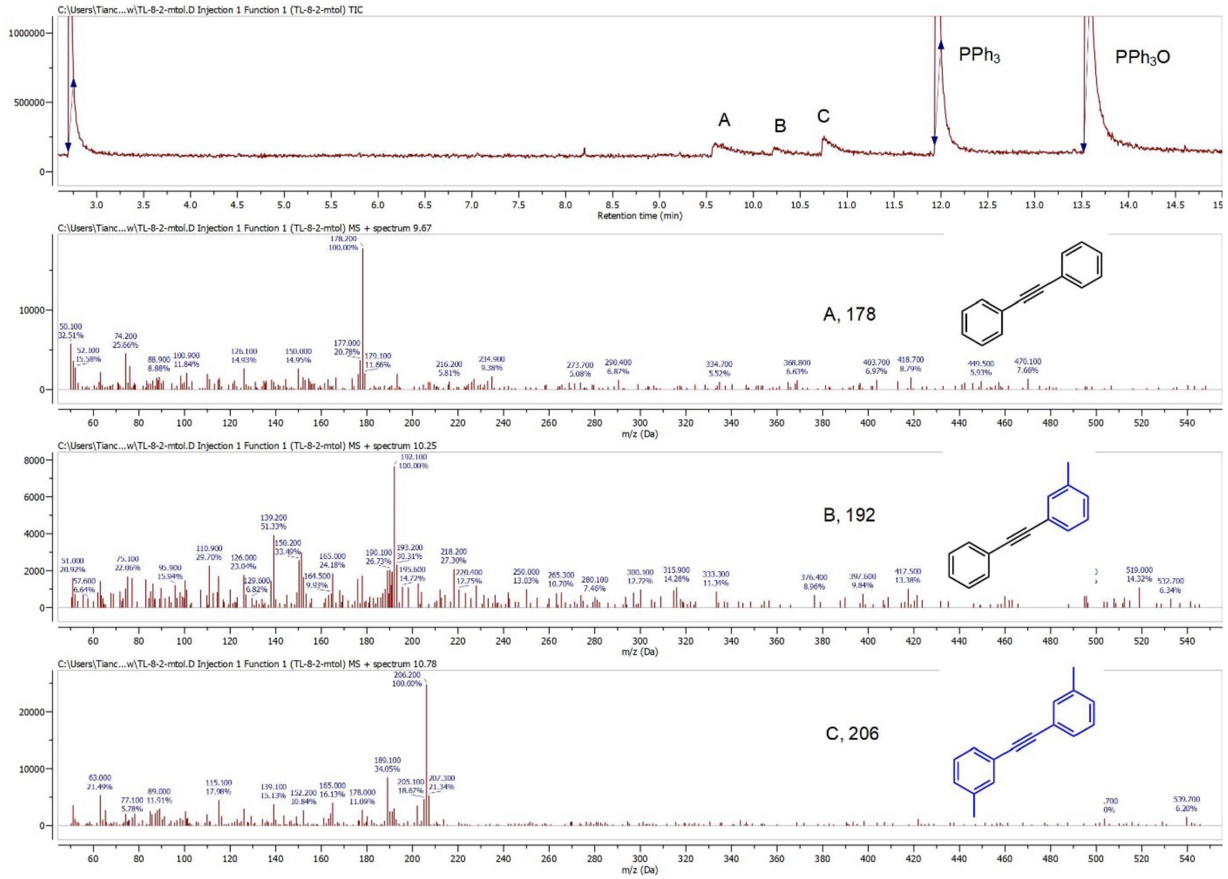

Figure S33. GC-MS data for the σ-bond metathesis of PhCCPh and $Ar^{Me}CCAr^{Me}$ (Ar^{Me} = 3-Me-Ph).

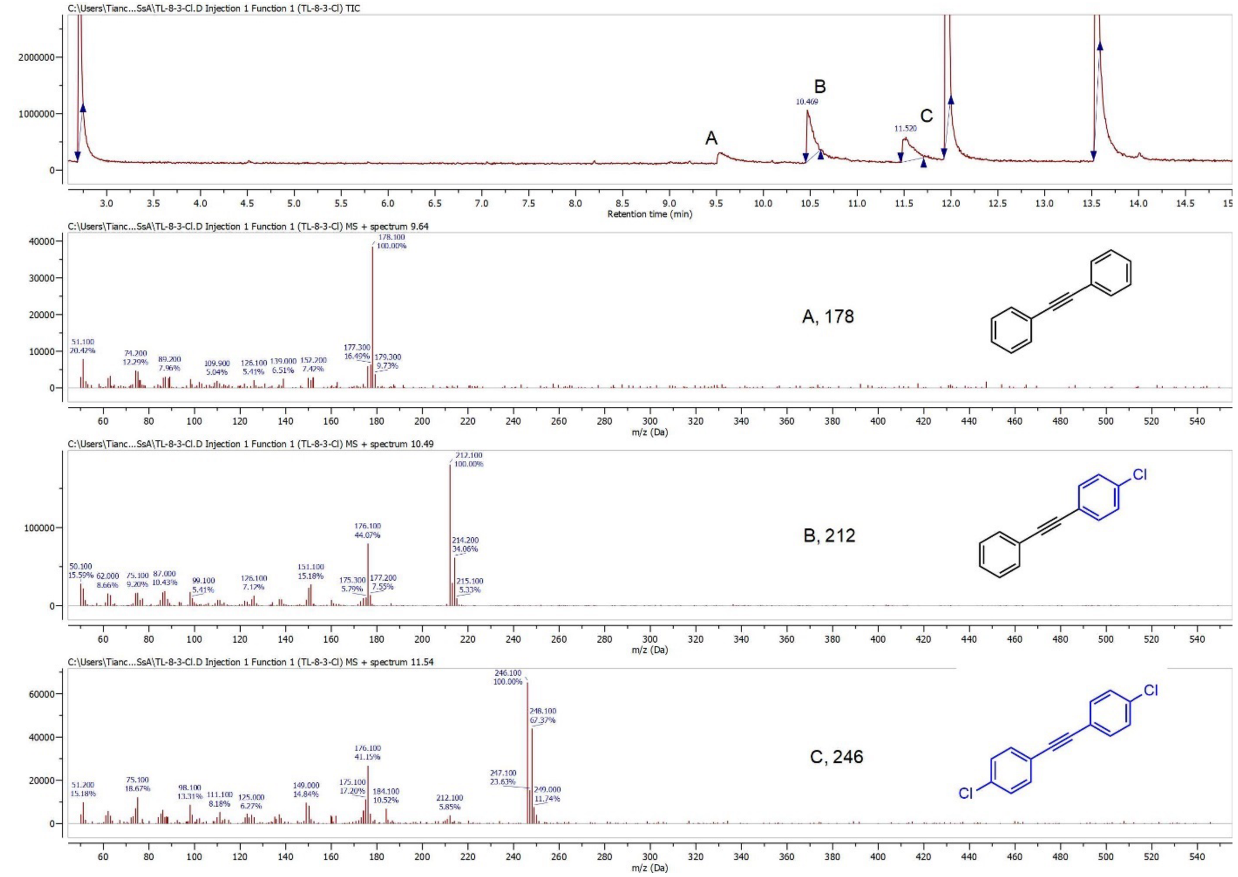

Figure S34. GC-MS data for the σ-bond metathesis of PhCCPh and $Ar^{Cl}CCAr^{Cl}$ (Ar^{Cl} = 4-Cl-Ph).

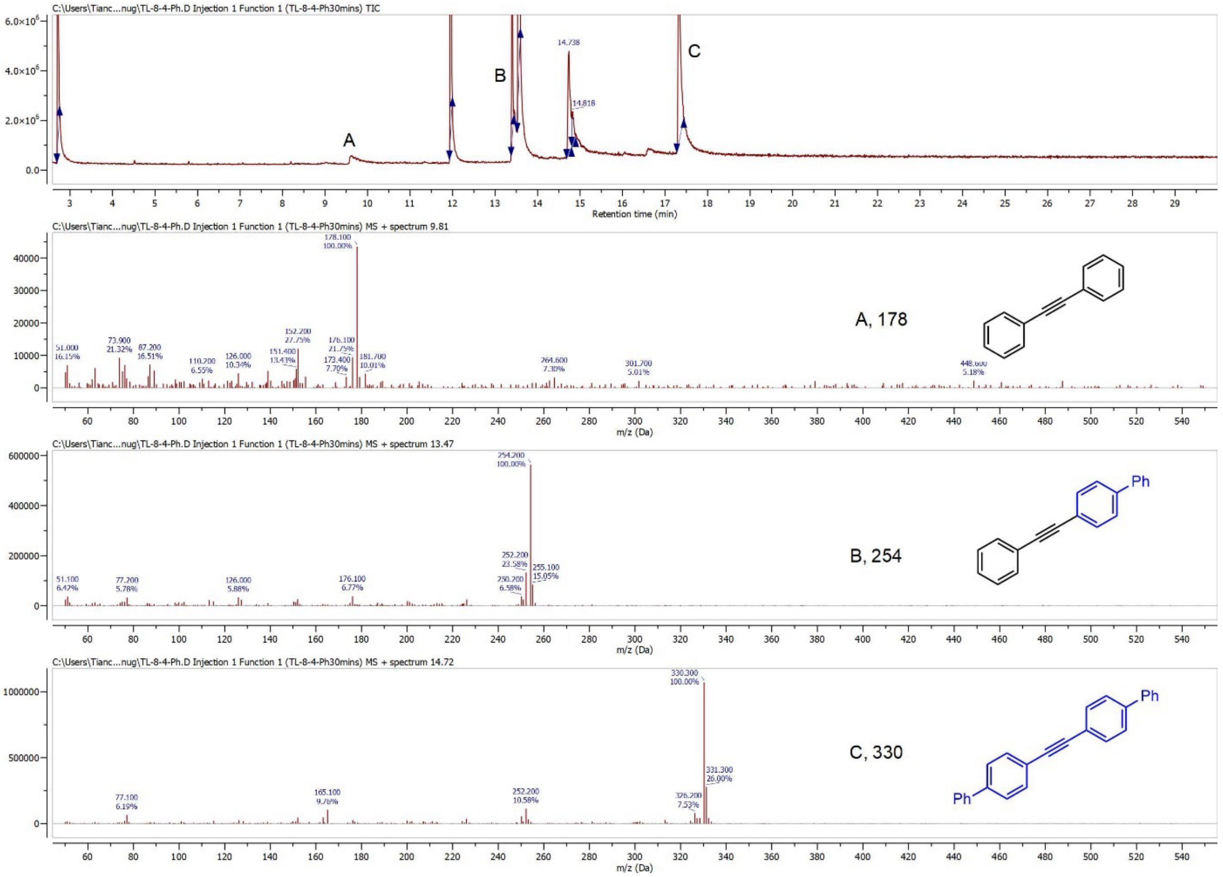

Figure S35. GC-MS data for the σ-bond metathesis of PhCCPh and $Ar^{Ph}CCAr^{Ph}$ (Ar^{Ph} = 4-Ph-Ph).

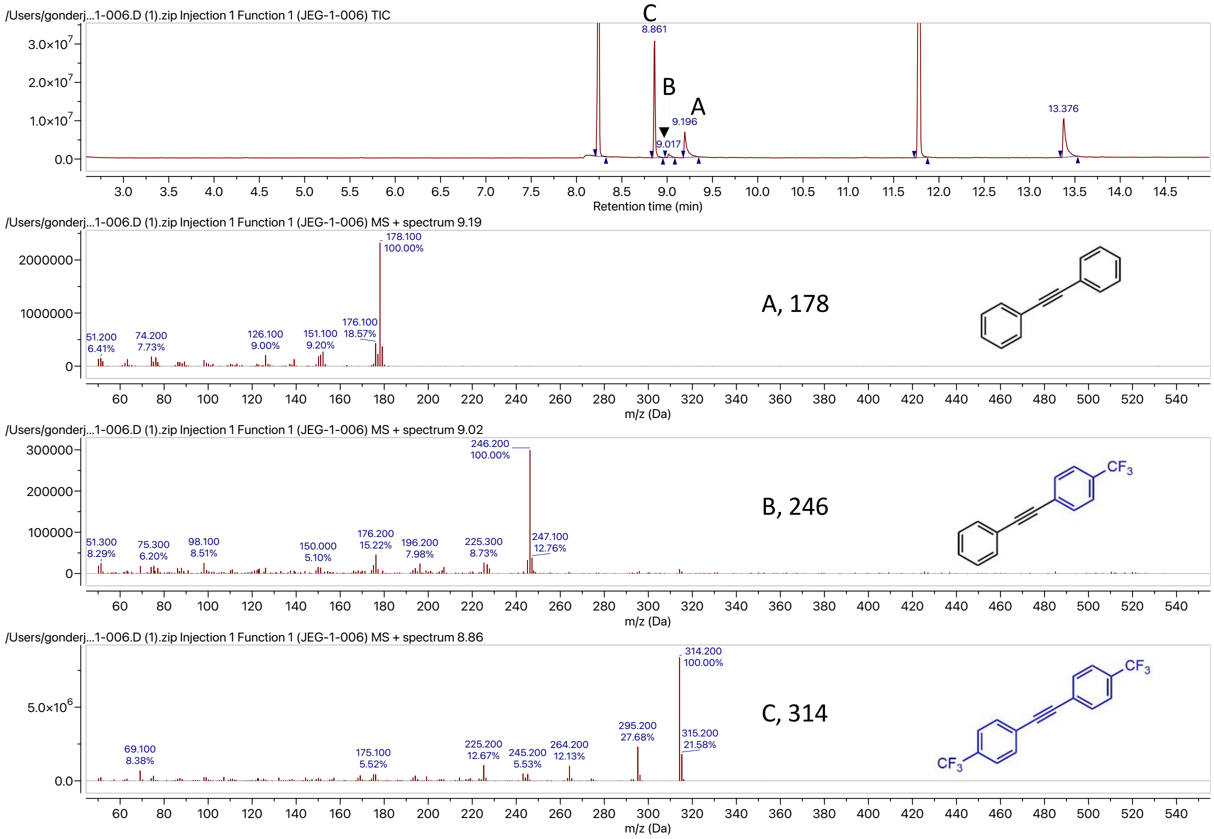

Figure S36. GC-MS data for the σ-bond metathesis of PhCCPh and $Ar^{CF3}CCAr^{CF3}$ (Ar^{CF3} = 4-CF_3-Ph).

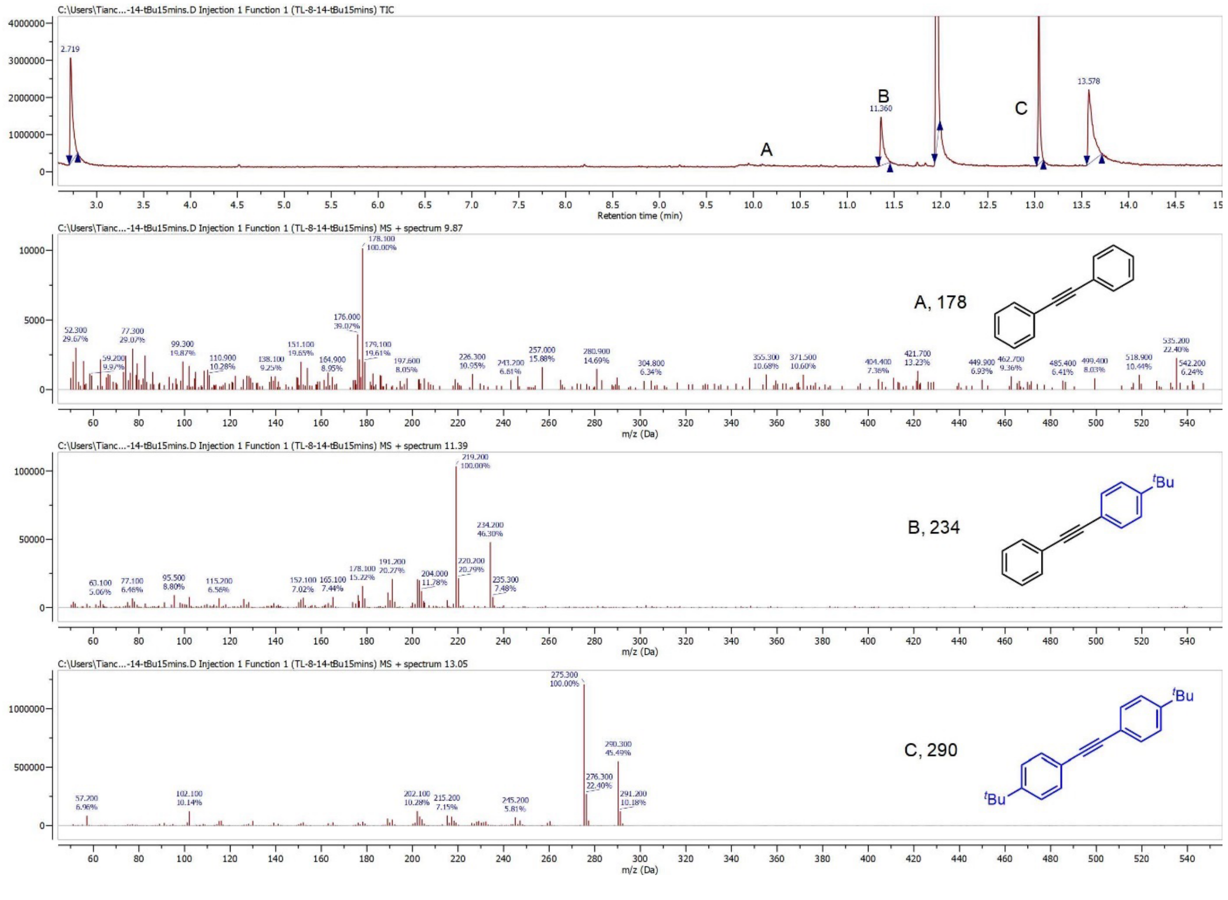

Figure S37. GC-MS data for the σ-bond metathesis of PhCCPh and $Ar^{tBu}CCAr^{tBu}$ (Ar^{tBu} = 4-^{t}Bu-Ph).

Summary of Crystal Data

Table S1. Summary of crystallographic data for compounds reported in this work.

Complexes	$\mathbf{1^{Me,OEt}}$ / $\mathbf{1^{OEt,Me}}$	**[3][BAr$^F{}_4$] •1.5Et$_2$O**	**[4][PF$_6$]$_2$•1.5PhF**
CCDC entry number	2375354	2375355	2375356
Empirical formula	$C_{73}H_{92}Fe_2N_6O_{2.5}P$	$C_{114}H_{108}BF_{24}Fe_2N_6O_{1.5}P_2$	$C_{73}H_{78.5}F_{13.5}Fe_2N_6P_3$
Formula weight	1236.19	2226.51	1501.02
Temperature/K	100	100	100
Crystal system	monoclinic	triclinic	monoclinic
Space group	P2$_1$/n	P$\bar{1}$	P2$_1$/n
a/Å	10.3808(4)	14.09570(10)	24.4482(3)
b/Å	21.0758(7)	20.1889(2)	11.9450(2)
c/Å	30.3435(10)	21.1235(2)	26.3761(4)
α/°	90	66.9760(10)	90
β/°	97.414(3)	76.1840(10)	109.540(2)
γ/°	90	76.6860(10)	90
Volume/Å^3	6583.2(4)	5308.95(9)	7259.1(2)
Z	4	2	4
ρ_{calc}/g•cm^{-3}	1.247	1.393	1.373
μ/mm^{-1}	0.516	3.299	0.546
F(000)	2636.0	2298.0	3108.0
Crystal size/mm^3	0.3 × 0.09 × 0.02	0.27 × 0.15 × 0.06	0.62 × 0.28 × 0.07
Radiation	Mo K_α (λ = 0.71073)	Cu K_α (λ = 1.54184)	Mo K_α (λ = 0.71073)
2*θ* range for data collection/°	4.72 - 56.564	5.35 - 149.002	3.782 - 56.564
Index ranges	-13 ≤ h ≤ 13<br>-28 ≤ k ≤ 26<br>-40 ≤ l ≤ 40	-17 ≤ h ≤ 17<br>-25 ≤ k ≤ 24<br>-26 ≤ l ≤ 21	-32 ≤ h ≤ 32<br>-15 ≤ k ≤ 15<br>-35 ≤ l ≤ 35
Reflections collected	102608	81595	164937
Independent reflections	16303 [R_{int} = 0.0564]	21228 [R_{int} = 0.0337]	17997 [R_{int} = 0.0402]
Data/restraints/parameters	16303/532/980	21228/357/1562	17997/330/1010
Goodness-of-fit on F^2	1.051	1.056	1.061
Final R indexes [I>=2σ (I)]	R_1 = 0.0554,<br>wR_2 = 0.1279	R_1 = 0.0426,<br>wR_2 = 0.1111	R_1 = 0.0445,<br>wR_2 = 0.1324
Final R indexes [all data]	R_1 = 0.0753,<br>wR_2 = 0.1361	R_1 = 0.0508,<br>wR_2 = 0.1189	R_1 = 0.0518,<br>wR_2 = 0.1373
Largest diff. peak/hole / e•Å^{-3}	1.01/-0.69	0.53/-0.61	1.10/-0.58

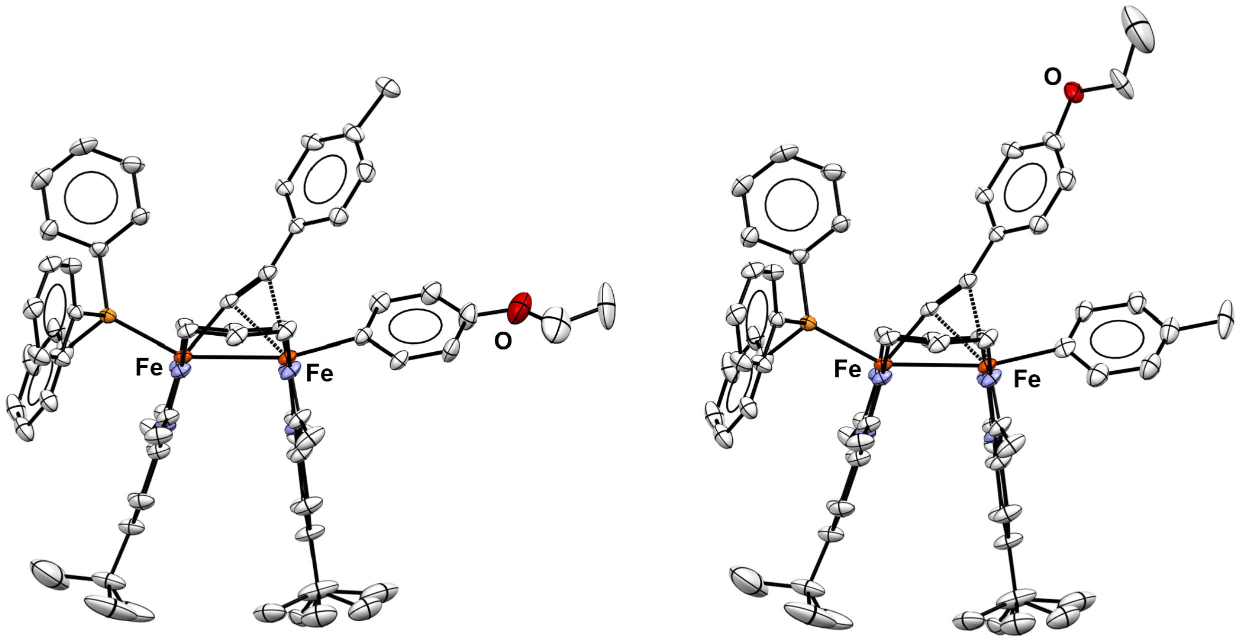

Figure S38. Crystal structure of co-crystallized **1**$^{\mathbf{Me,OEt}}$ (left) and **1**$^{\mathbf{OEt,Me}}$ (right), with thermal ellipsoids at the 50% probability level. The hydrogen atoms were omitted for clarity.

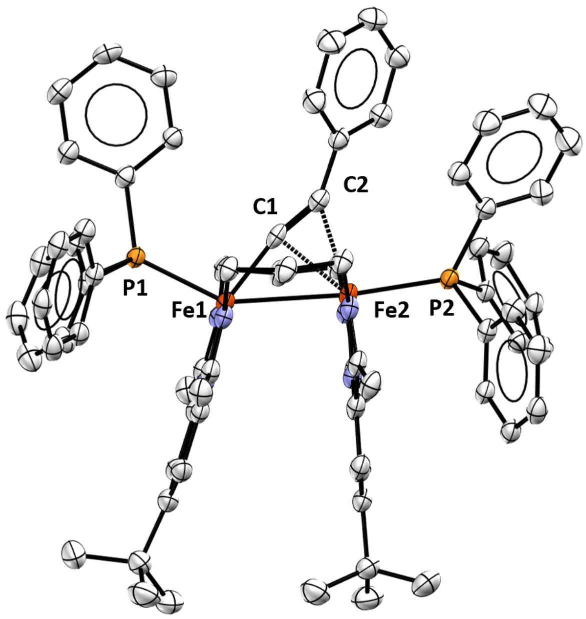

Figure S39. Crystal structure of **[3][BAr$^{\mathbf{F}}_{\mathbf{4}}$]** with thermal ellipsoids at the 50% probability level. The counteranions, solvent molecules and hydrogen atoms were omitted for clarity.

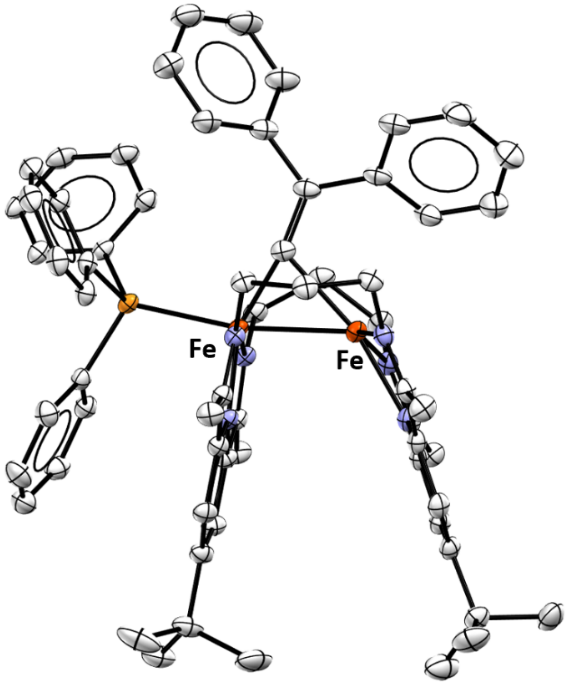

Figure S40. Crystal structure of **[4][PF$_6$]$_2$**, with thermal ellipsoids at the 50% probability level. The counteranions, solvent molecules and hydrogen atoms were omitted for clarity.

Determination of the acceptor number of Lewis acids via NMR experiments.

General Procedure: A THF solution of triethylphosphine oxide (0.083 M, 0.6 mL, 0.049 mmol) was treated with a Lewis acid (0.050 mmol, 1.02 equiv). After vigorous stirring, the mixture was transferred into an NMR tube and the $^{31}P\{^1H\}$ NMR chemical shift of the product ($\delta(Et_3P{=}O)$) was measured. The acceptor number (AN) was then calculated using the Gutmann-Beckett method[12]:

$$AN = \Delta\delta * [100/(86.14-41)]$$

The results are summarized below:

**LA**	**$\delta(Et_3P{=}O)$**	**$\Delta\delta$**	**AN**
None	44.83	0	0
$ZnEt_2$	50.98	6.15	14
LiCl	55.85	11.02	24
$ZnPh_2$	58.61	13.78	31
$MgCl_2$	63.61	18.78	42
ZnI_2	66.50	21.67	48
$ZnBr_2$	67.00	22.17	49
$ZnCl_2$	67.64	22.81	51
$AlCl_3$	79.62	34.79	77
BCl_3	81.44	36.61	81
$TiCl_4(thf)_2$	81.99	37.16	82

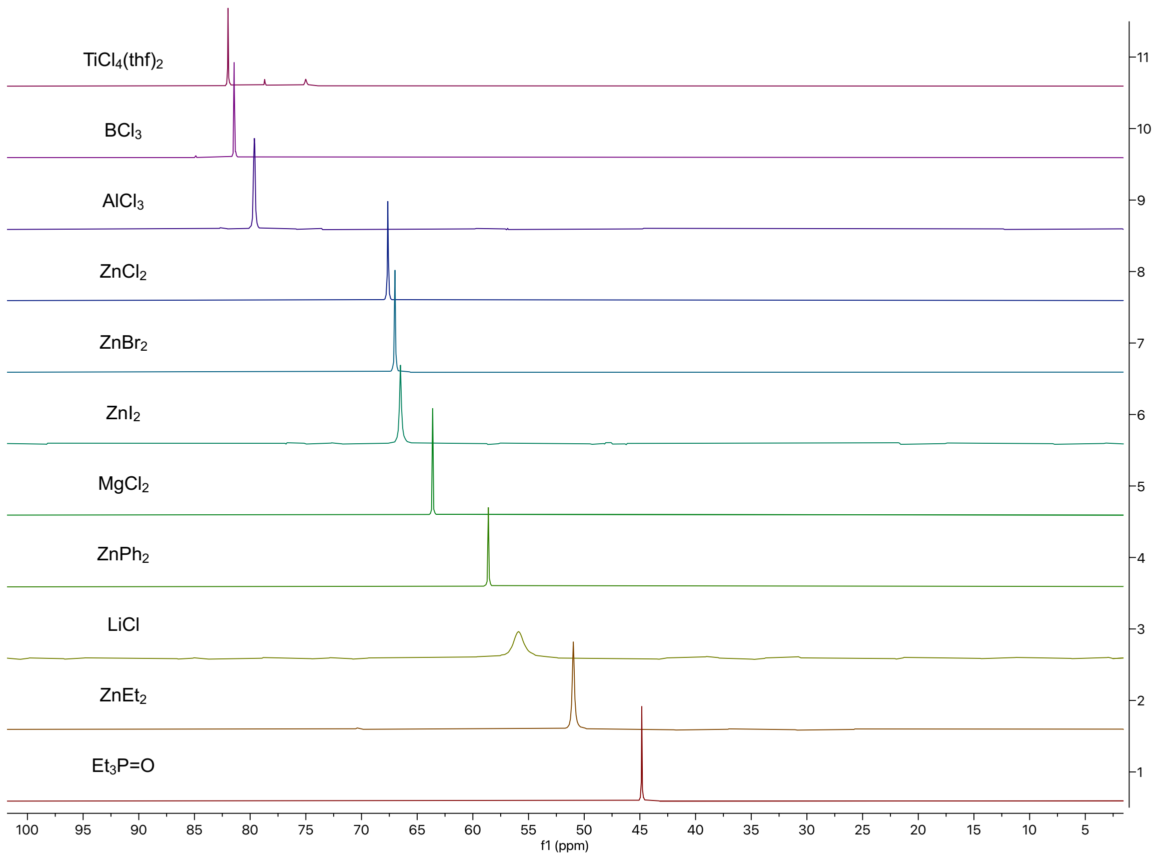

Figure S41. Overlay of $^{31}P\{^{1}H\}$ NMR spectra in THF (162 MHz, 298 K) for the reactions of $Et_3P{=}O$ with various Lewis acids.

NMR Spectroscopic Investigation of Fe-Fe Exchange Reactions.

General Procedure: In a typical experiment, co-crystallized $\mathbf{1}^{\mathbf{Me,OEt}}$ and $\mathbf{1}^{\mathbf{OEt,Me}}$ and a known amount of 1,2,4,5-tetramethylbenzene, which was used as internal standard, were loaded into a J. Young NMR tube and dissolved with 0.8 mL of THF-d_8. ^{1}H and ^{31}P{^{1}H} NMR spectra were collected, then a THF solution of the Lewis acid was added by microsyringe. The reaction mixture was shaken briefly to allow for thorough mixing of reactants, and reaction progress was monitored via ^{1}H and ^{31}P{^{1}H} NMR spectroscopic measurements.

Generalized Scheme:

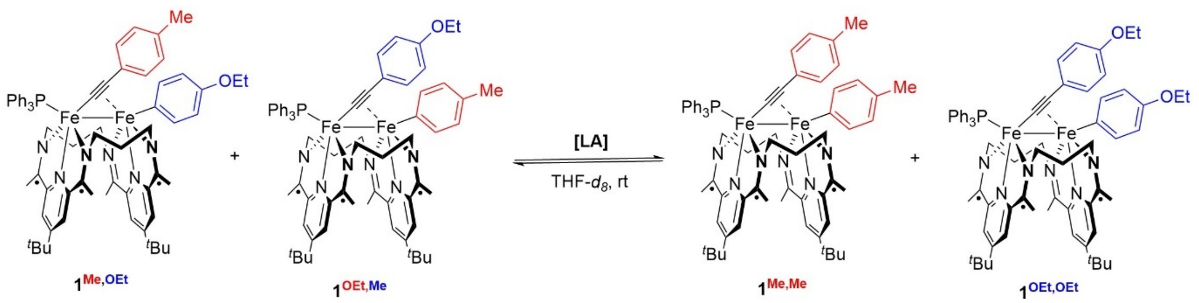

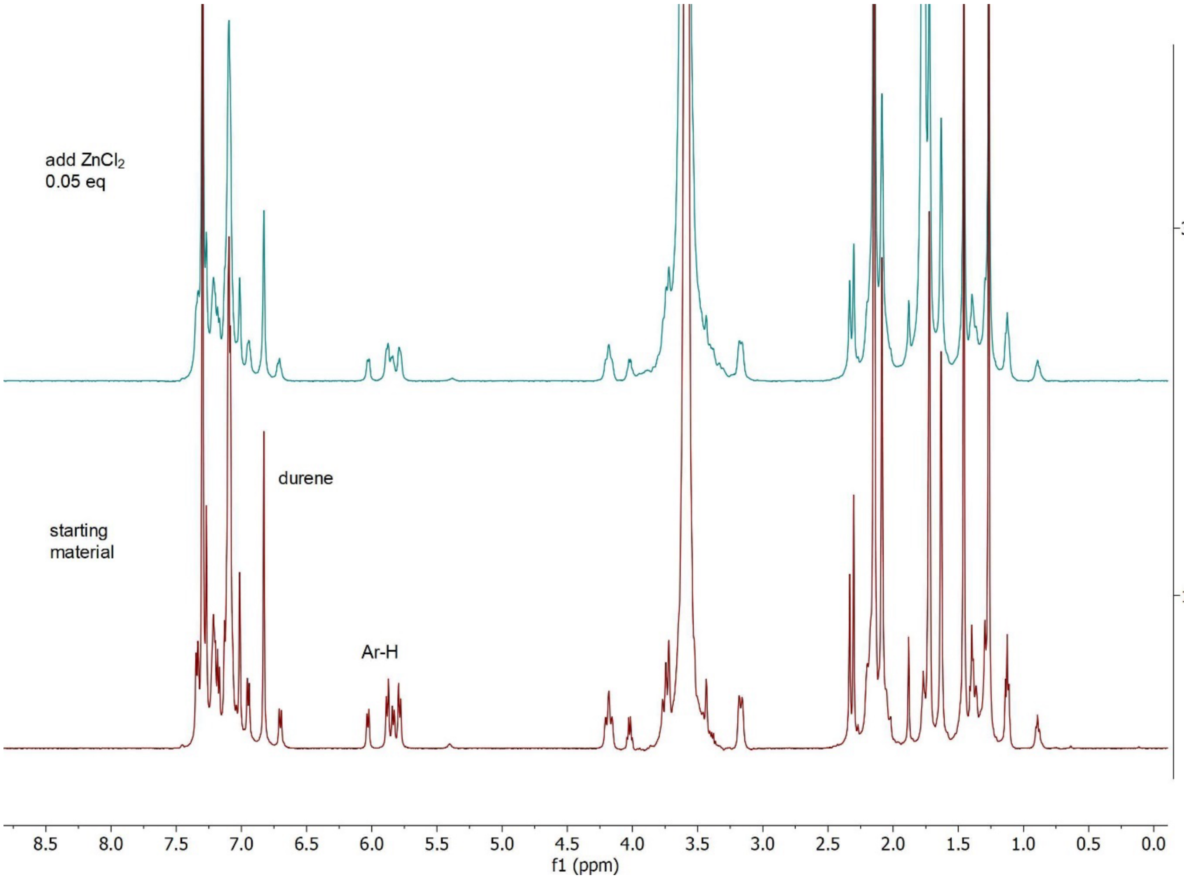

Figure S42. ^{1}H NMR spectra for the Fe-Fe exchange reaction mediated by $ZnCl_2$ between $\mathbf{1}^{\mathbf{Me,OEt}}$ and $\mathbf{1}^{\mathbf{OEt,Me}}$, showing before (bottom) and after (top) the addition of the Lewis acid in THF-d_8 (500 MHz, 298 K).

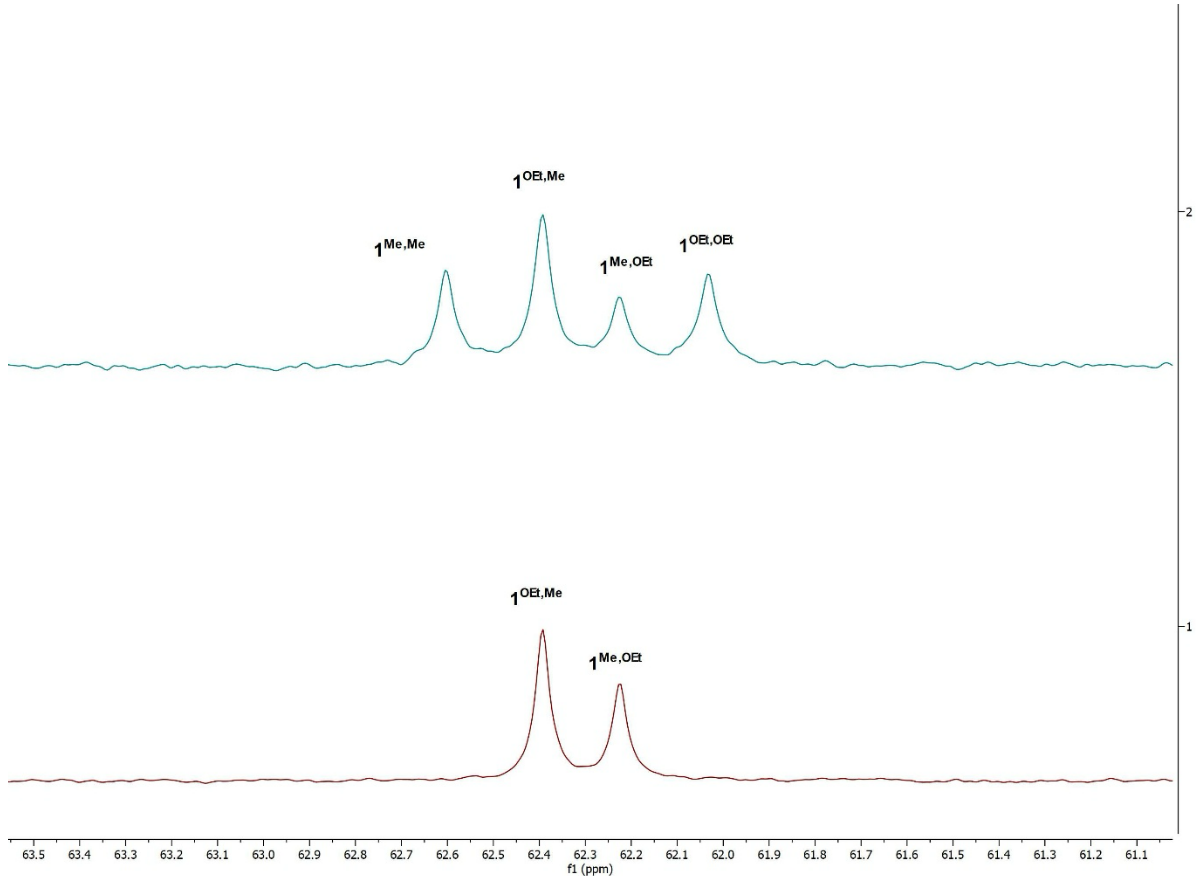

Figure S43. $^{31}P\{^{1}H\}$ NMR spectra for the Fe-Fe exchange reaction mediated by $ZnCl_2$ between $\mathbf{1}^{\mathbf{Me,OEt}}$ and $\mathbf{1}^{\mathbf{OEt,Me}}$, showing before (bottom) and after (top) the addition of the Lewis acid in THF-d_8 (162 MHz, 298 K).

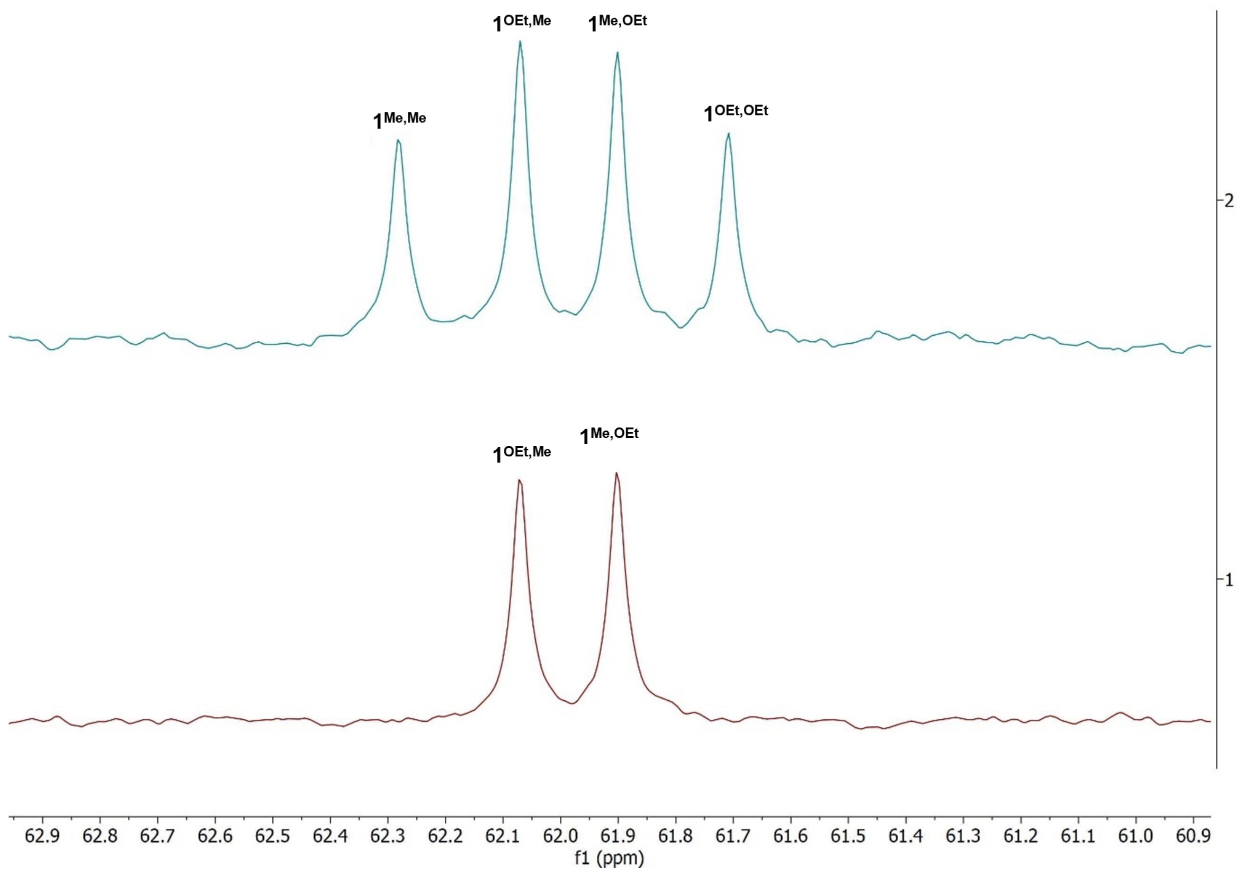

Figure S44. $^{31}P\{^{1}H\}$ NMR spectra for the Fe-Fe exchange reaction mediated by $MgCl_2$ between $\mathbf{1}^{\mathbf{Me,OEt}}$ and $\mathbf{1}^{\mathbf{OEt,Me}}$ in THF-d_8 (162 MHz, 298 K), showing before (bottom) and after (top) the addition of the Lewis acid.

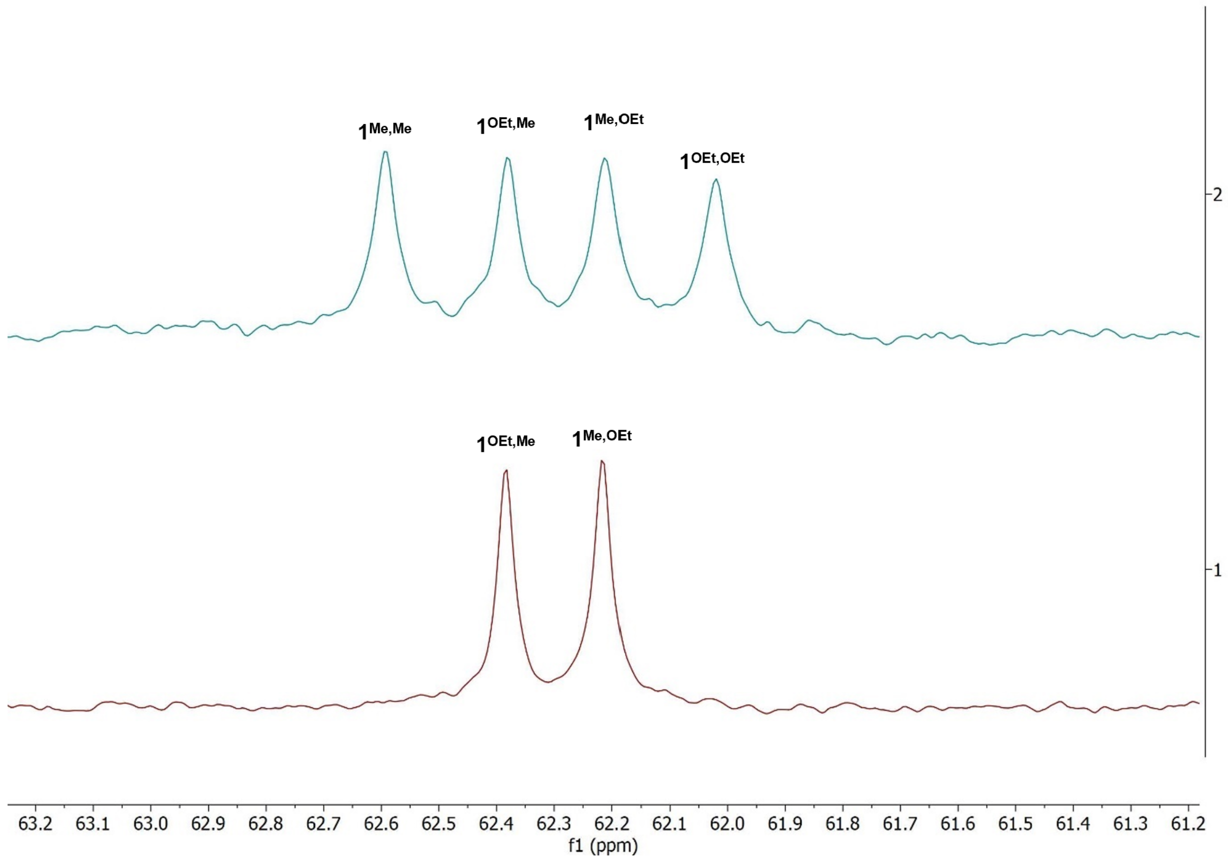

Figure S45. $^{31}P\{^{1}H\}$ NMR spectra for the Fe-Fe exchange reaction mediated by $AlCl_3$ between $\mathbf{1}^{\mathbf{Me,OEt}}$ and $\mathbf{1}^{\mathbf{OEt,Me}}$ in THF-d_8 (298 K), showing before (bottom) and after (top) the addition of the Lewis acid.

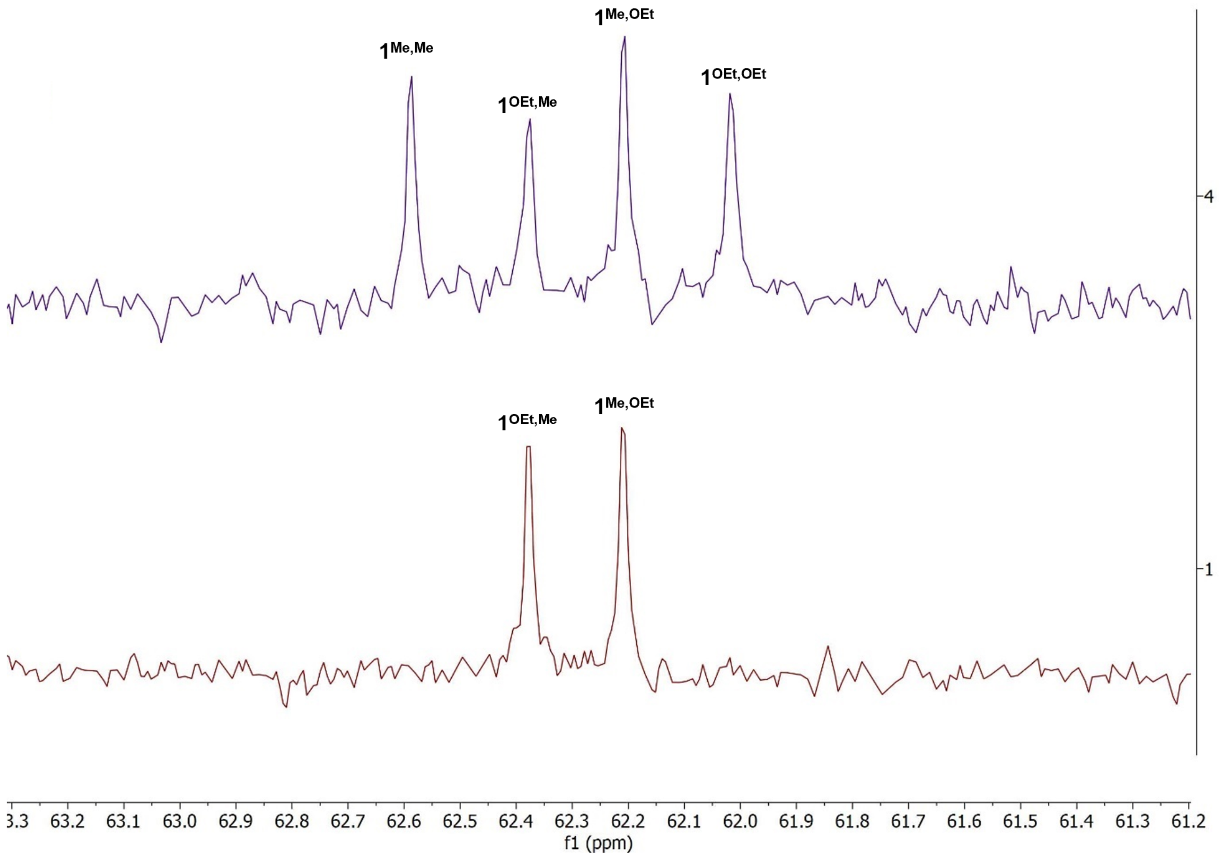

Figure S46. $^{31}P\{^{1}H\}$ NMR spectra for the Fe-Fe exchange reaction mediated by $TiCl_4(thf)_2$ between $\mathbf{1}^{\mathbf{Me,OEt}}$ and $\mathbf{1}^{\mathbf{OEt,Me}}$ in THF-d_8 (298 K), showing before (bottom) and after (top) the addition of the Lewis acid.

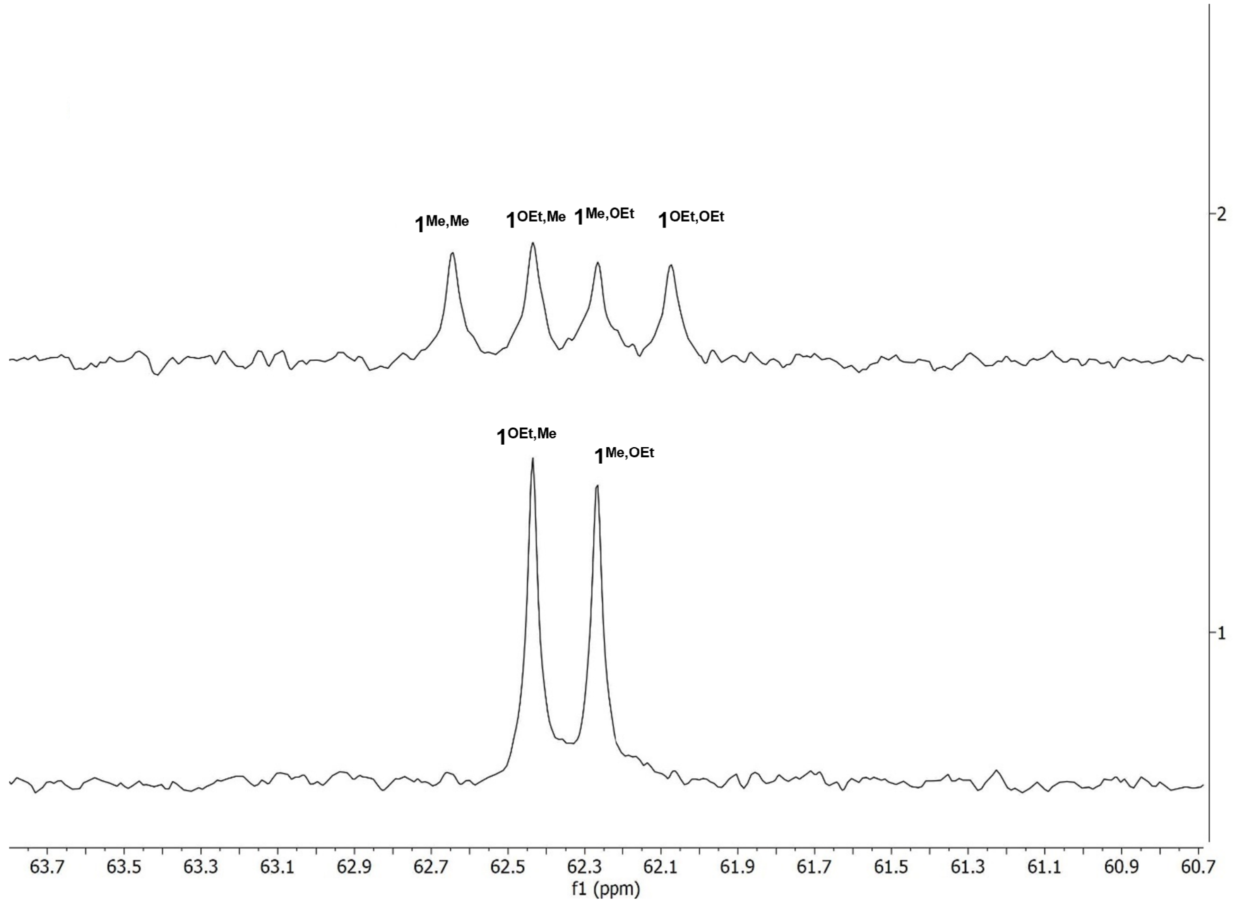

Figure S47. $^{31}P\{^{1}H\}$ NMR spectra for the Fe-Fe exchange reaction mediated by $ZnBr_2$ between $\mathbf{1}^{\mathbf{Me,OEt}}$ and $\mathbf{1}^{\mathbf{OEt,Me}}$ in THF-d_8 (298 K), showing before (bottom) and after (top) the addition of the Lewis acid.

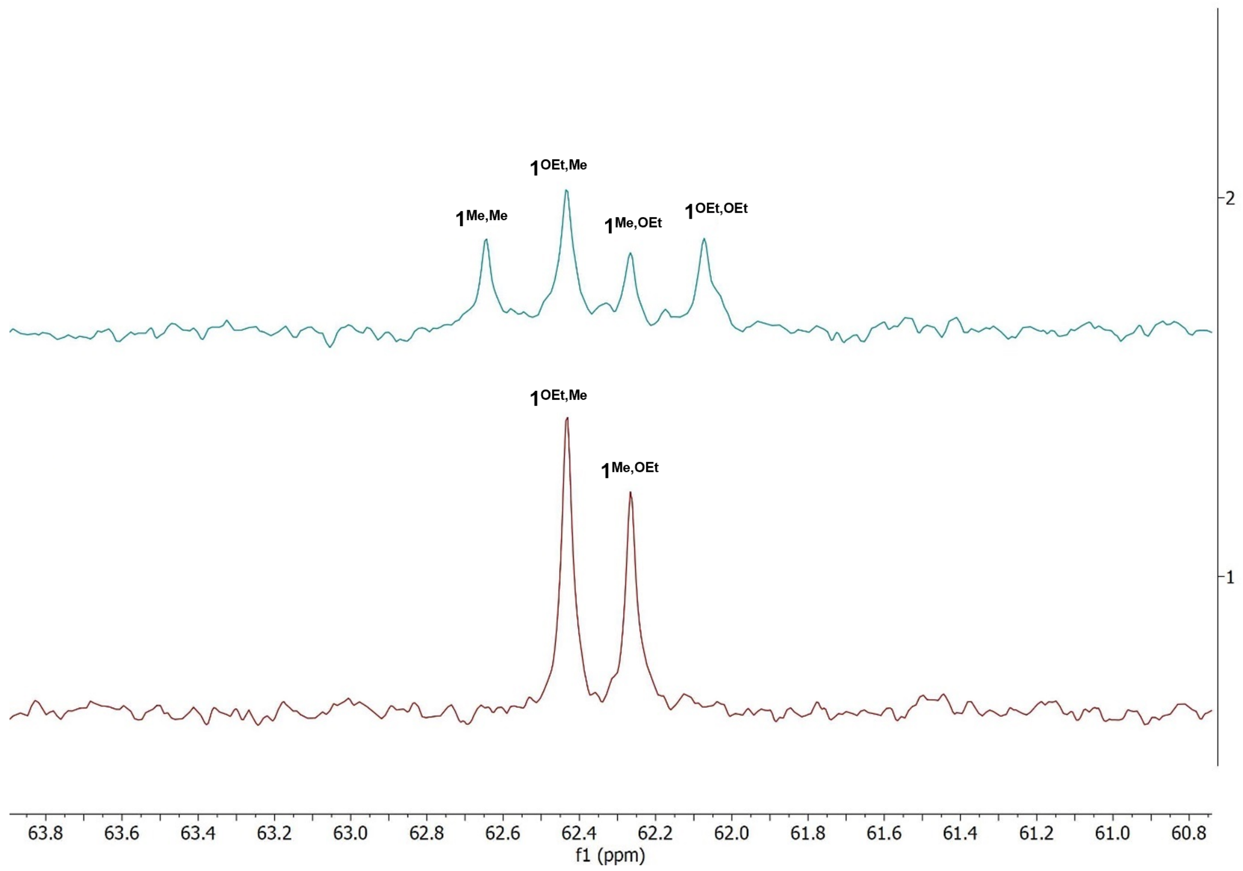

Figure S48. $^{31}P\{^{1}H\}$ NMR spectra for the Fe-Fe exchange reaction mediated by ZnI_2 between $\mathbf{1}^{\mathbf{Me,OEt}}$ and $\mathbf{1}^{\mathbf{OEt,Me}}$ in THF-d_8 (298 K), showing before (bottom) and after (top) the addition of the Lewis acid.

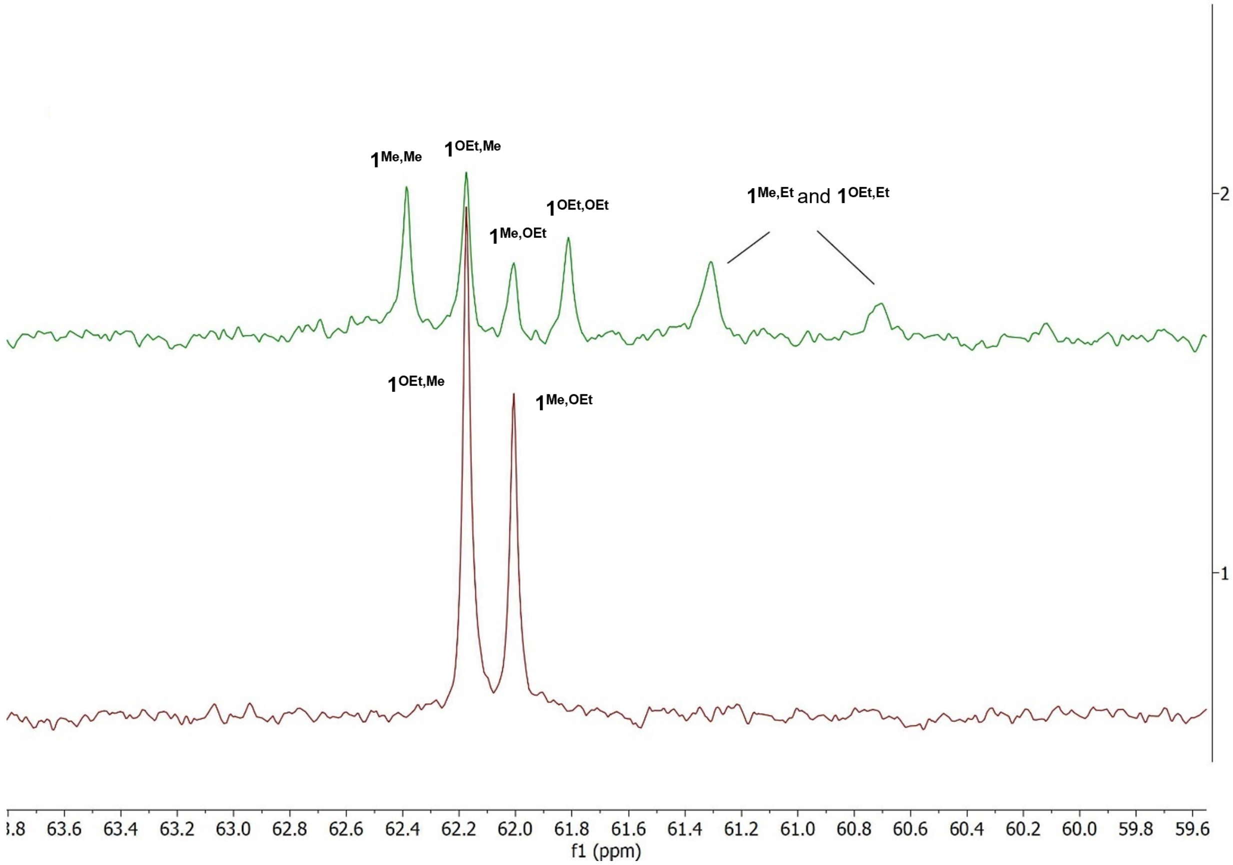

Figure S49. $^{31}P\{^{1}H\}$ NMR spectra for the Fe-Fe exchange reaction mediated by $ZnEt_2$ between $\mathbf{1}^{\mathbf{Me,OEt}}$ and $\mathbf{1}^{\mathbf{OEt,Me}}$ in THF-d_8 (298 K), showing before (bottom) and after (top) the addition of the Lewis acid. The top spectrum revealed the formation of both the desired exchange products and $\mathbf{1}^{\mathbf{Me,Et}}$/$\mathbf{1}^{\mathbf{OEt,Et}}$ (proposed) as side products via Fe-Zn exchange.

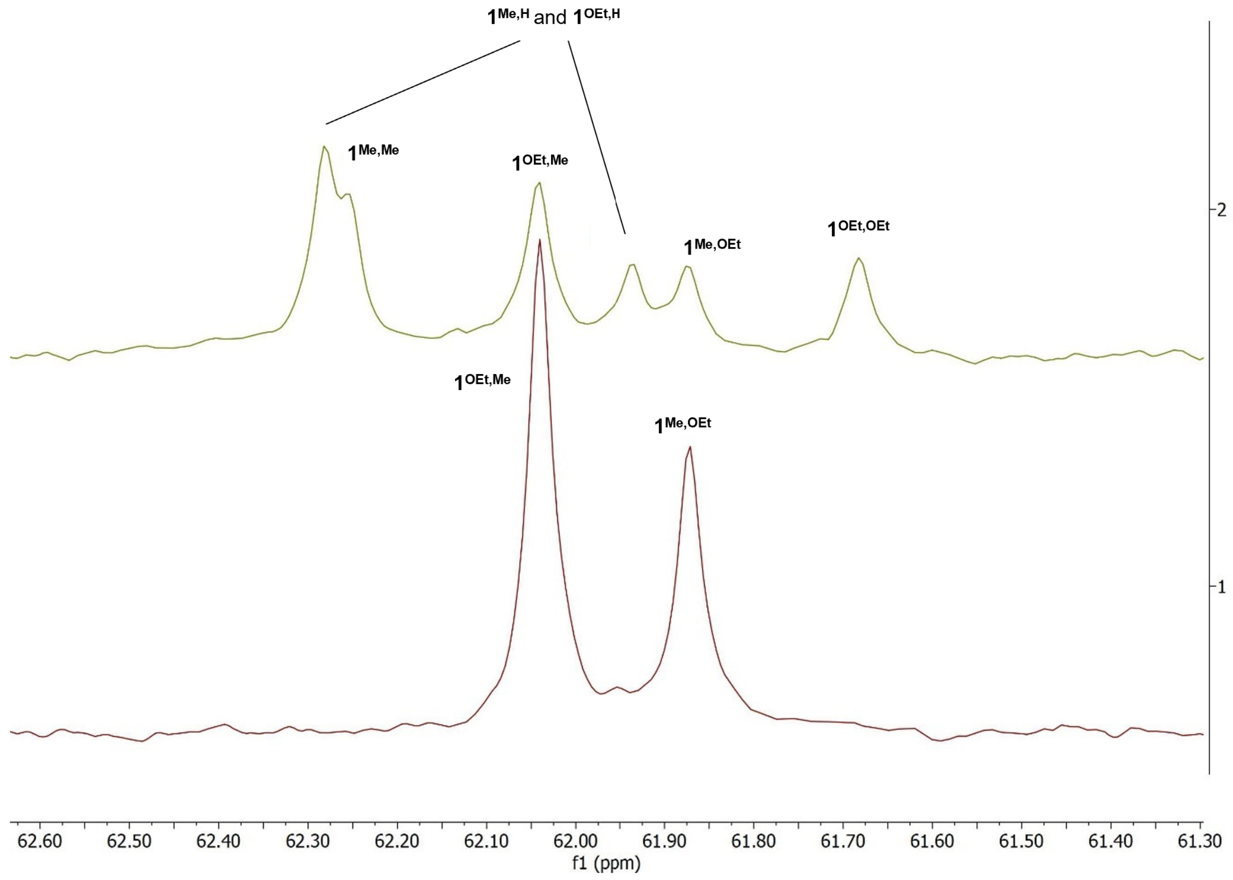

Figure S50. $^{31}P\{^{1}H\}$ NMR spectra for the Fe-Fe exchange reaction mediated by $ZnPh_2$ between $\mathbf{1}^{\mathbf{Me,OEt}}$ and $\mathbf{1}^{\mathbf{OEt,Me}}$ in THF-d_8 (298 K), showing before (bottom) and after (top) the addition of the Lewis acid. The top spectrum revealed the formation of both the desired exchange products and $\mathbf{1}^{\mathbf{Me,H}}$/$\mathbf{1}^{\mathbf{OEt,H}}$ (proposed) as side products via Fe-Zn exchange.

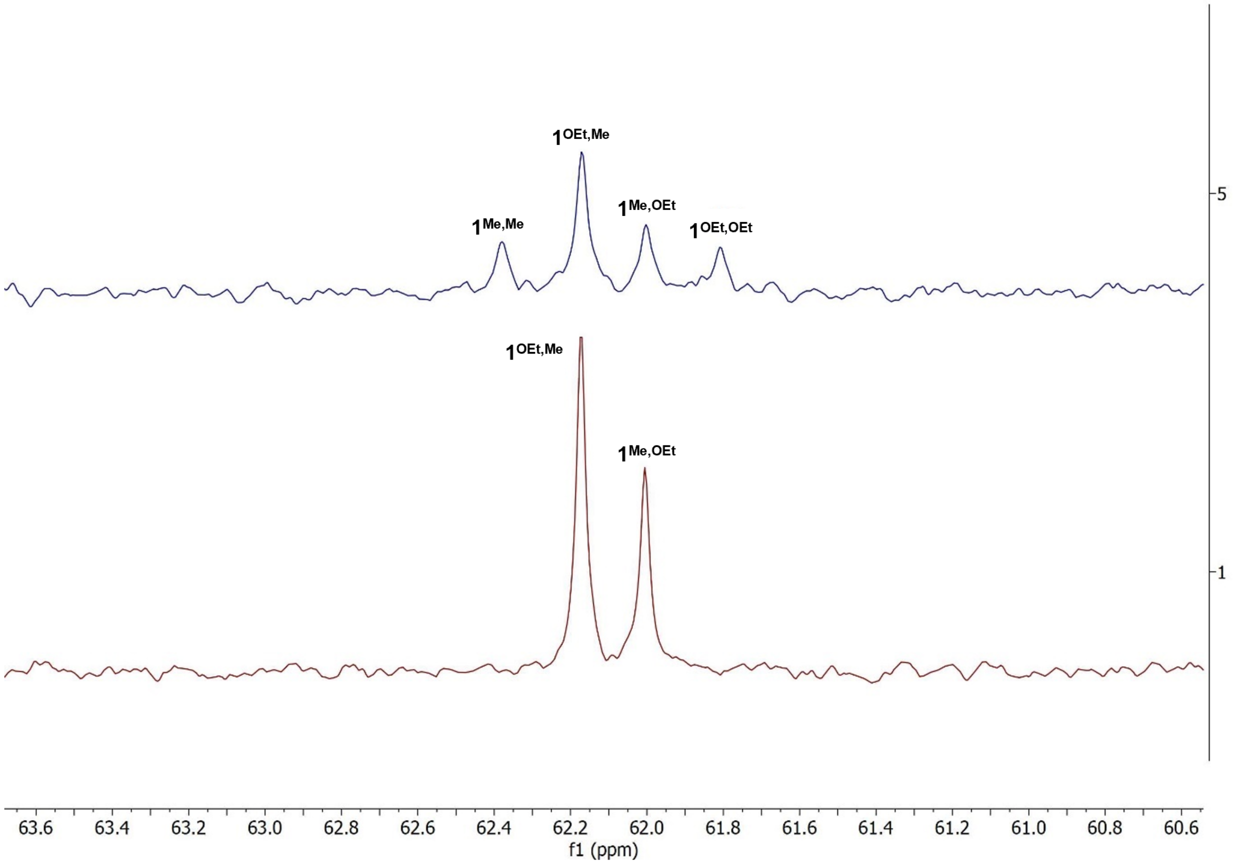

Figure S51. $^{31}P\{^{1}H\}$ NMR spectra for the Fe-Fe exchange reaction mediated by $Zn(OAc)_2$ between $\mathbf{1}^{Me,OEt}$ and $\mathbf{1}^{OEt,Me}$ in THF-d_8 (298 K), showing before (bottom) and after (top) the addition of the Lewis acid.

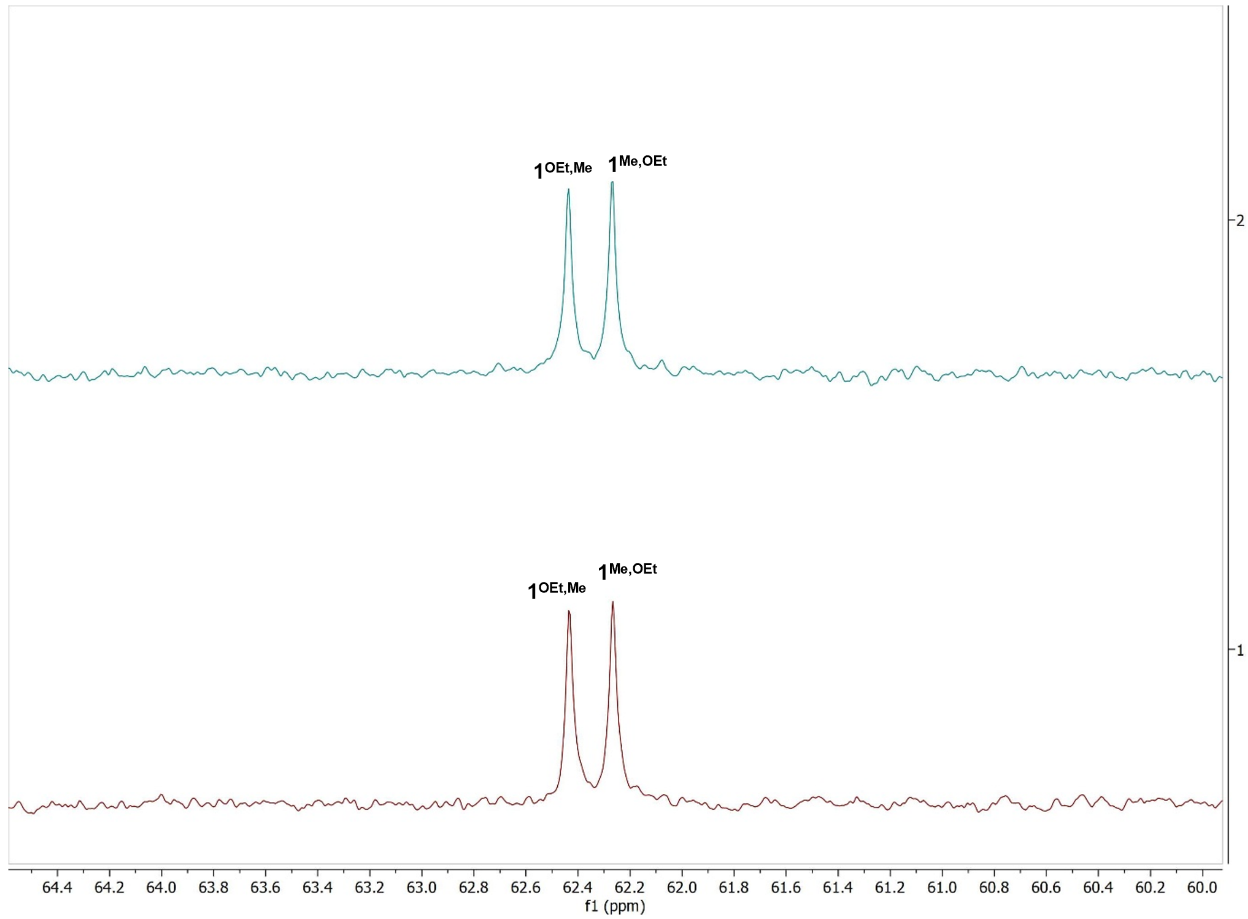

Figure S52. $^{31}P\{^{1}H\}$ NMR spectra of the attempted Fe-Fe exchange reaction of $\mathbf{1}^{\mathbf{Me,OEt}}$ and $\mathbf{1}^{\mathbf{OEt,Me}}$ with LiCl in THF-d_8 (298 K), showing before (bottom) and after (top) the addition of the Lewis acid.

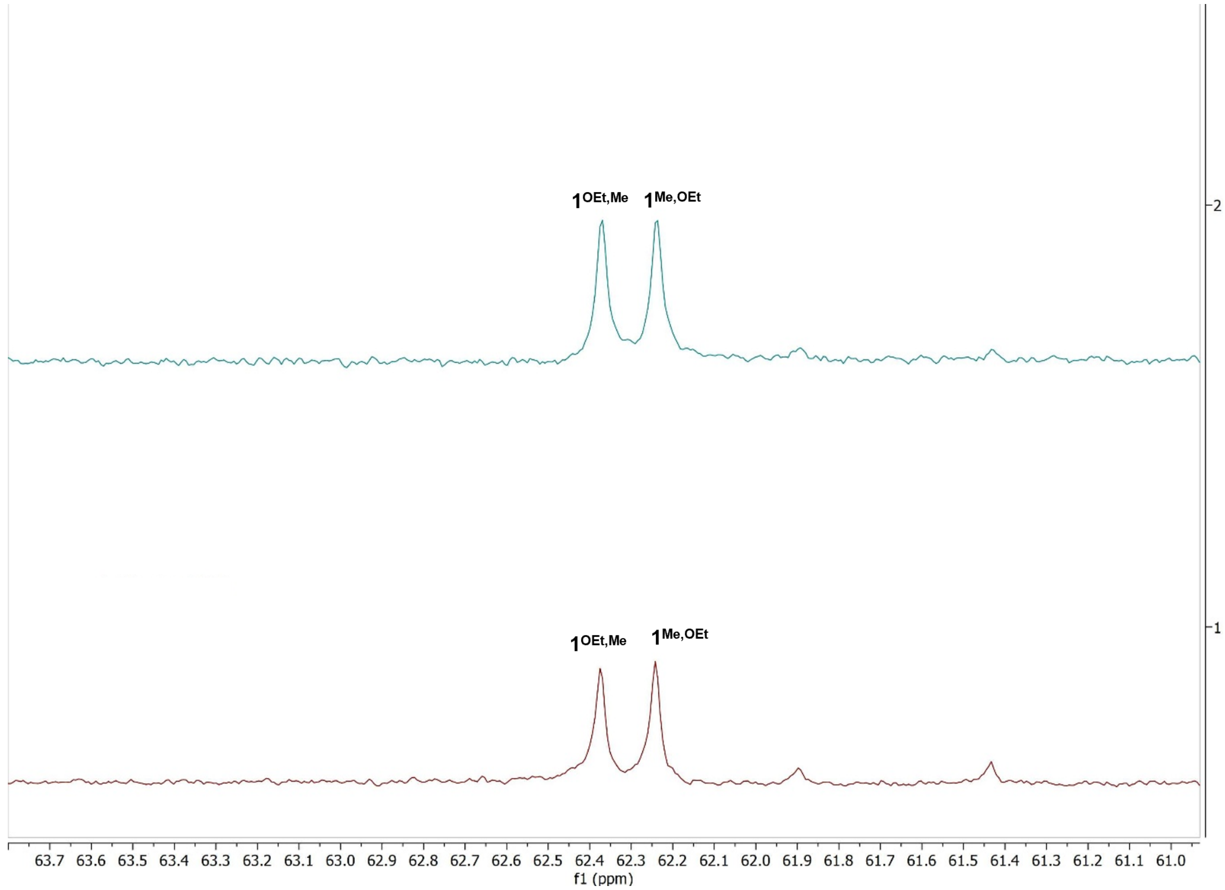

Figure S53. $^{31}P\{^{1}H\}$ NMR spectra of the attempted Fe-Fe exchange reaction of $\mathbf{1}^{\mathbf{Me,OEt}}$ and $\mathbf{1}^{\mathbf{OEt,Me}}$ with MeLi in C_6D_6 (298 K), showing before (bottom) and after (top) the addition of the Lewis acid.

NMR Spectroscopic Investigation of Fe-Zn Exchange Reactions.

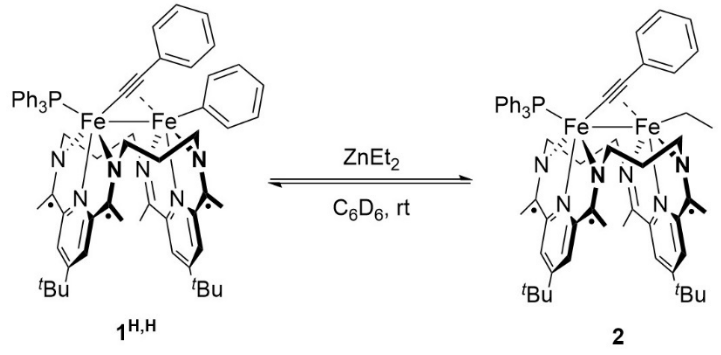

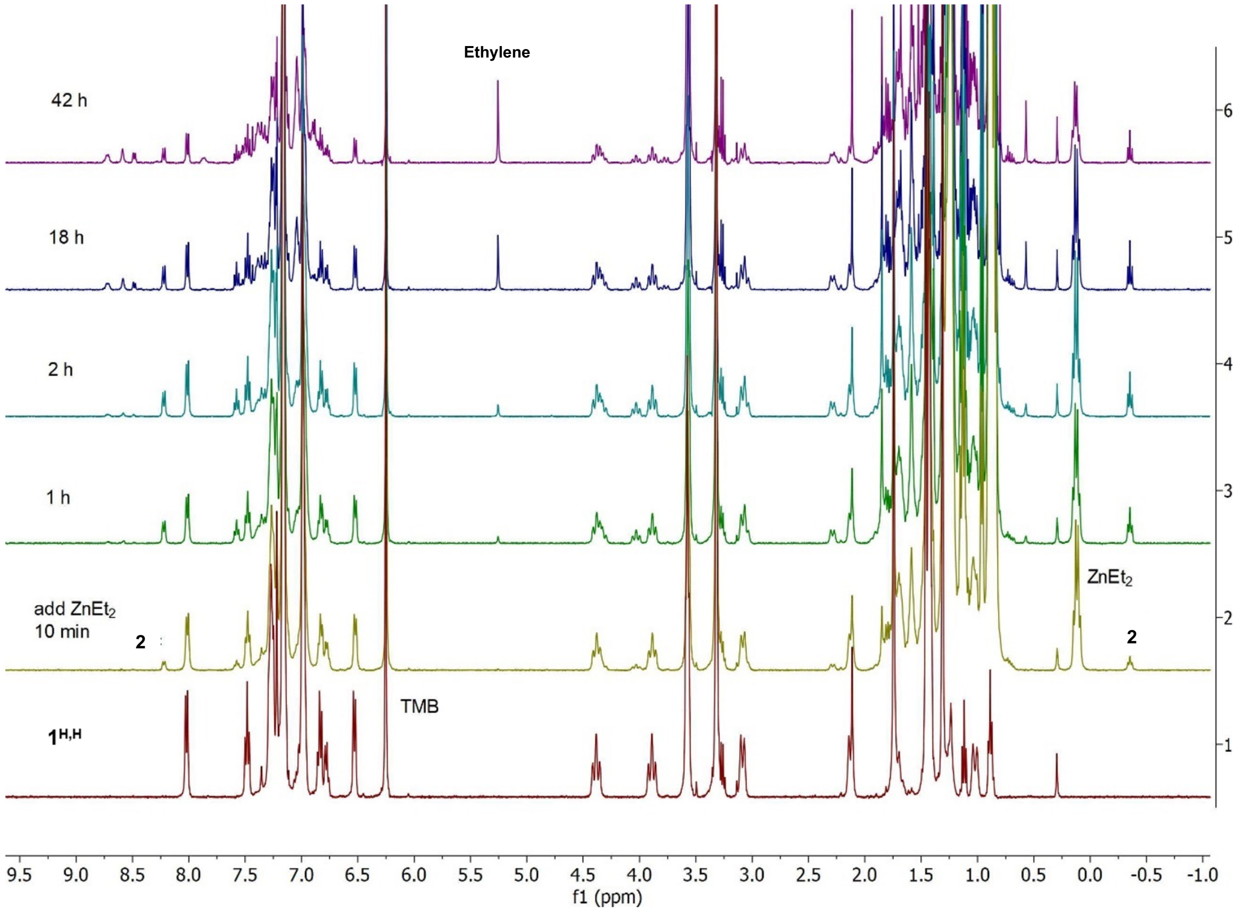

Figure S54. Fe-Zn exchange reaction of $\mathbf{1^{H,H}}$ with 1 equiv of $ZnEt_2$, as monitored by ^1H NMR spectroscopy in C_6D_6 (298 K).

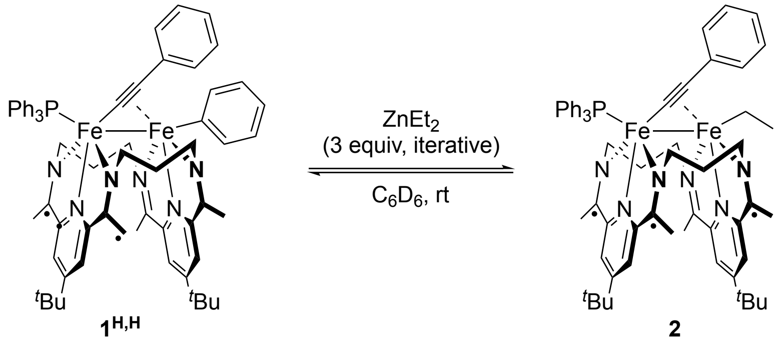

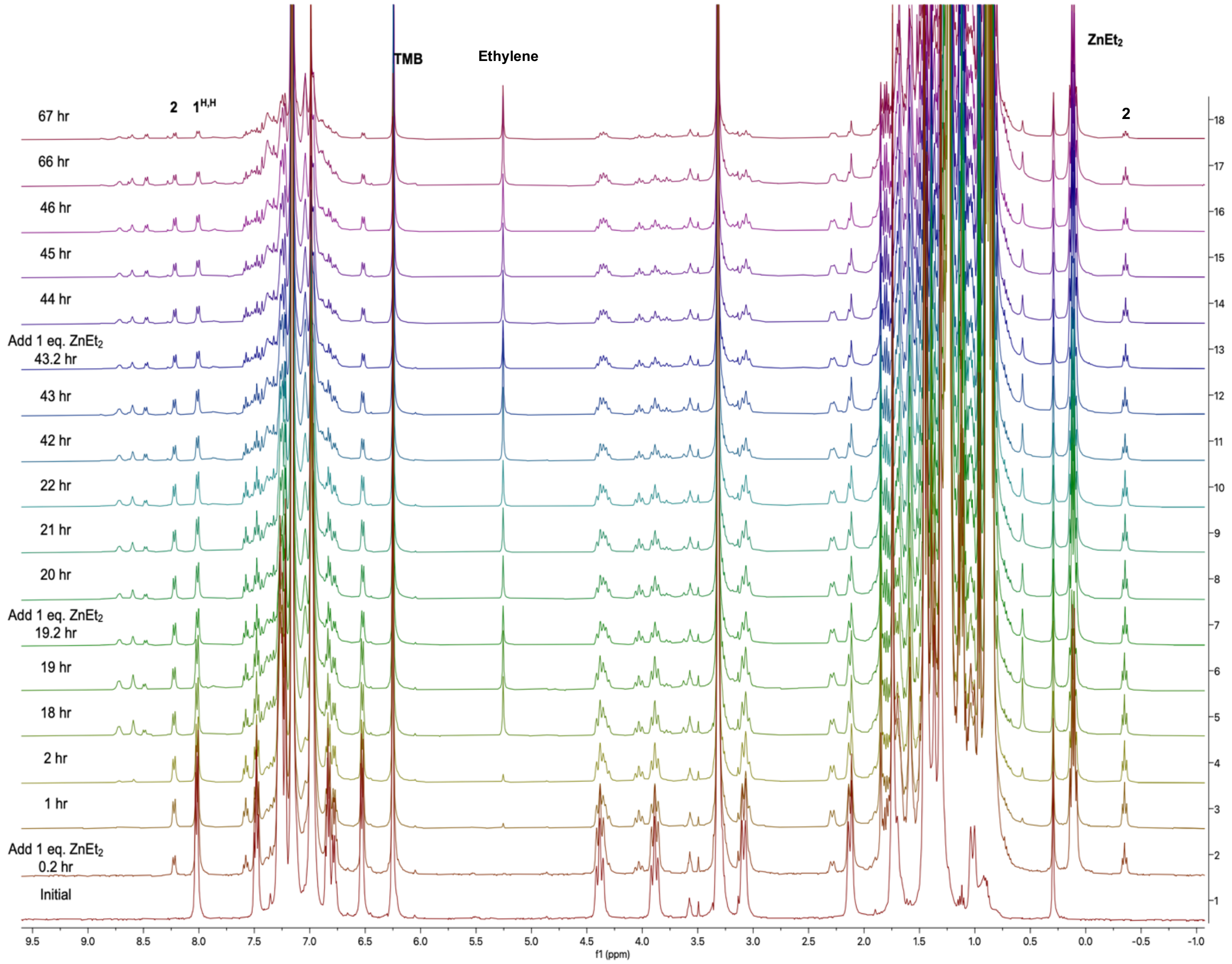

Figure S55. Fe-Zn exchange reaction of $\mathbf{1^{H,H}}$ with 3 equiv of $ZnEt_2$ added iteratively, as monitored by ^1H NMR spectroscopy in C_6D_6 (298 K).

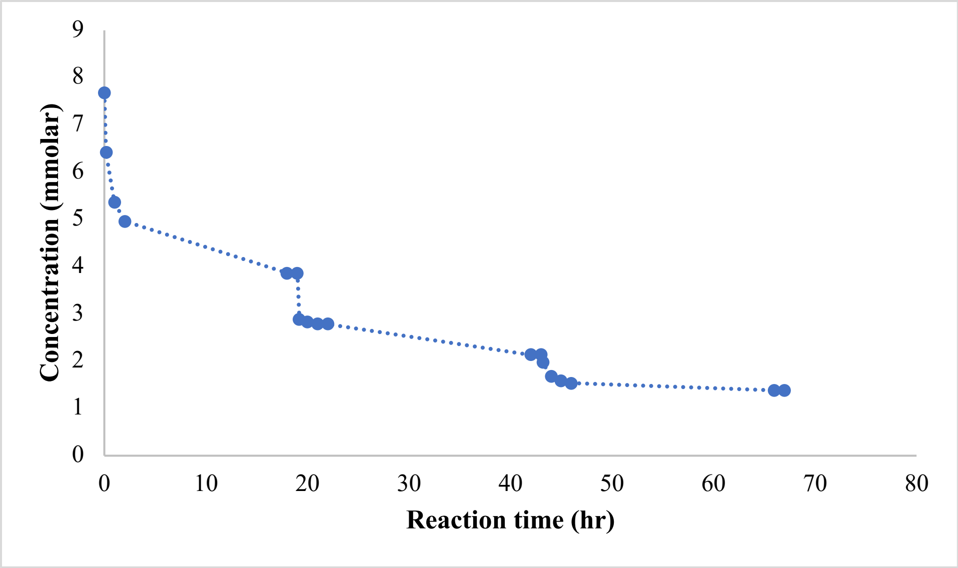

Figure S56. Graphical depiction of the concentration of $\mathbf{1}^{H,H}$ as a function of reaction time.

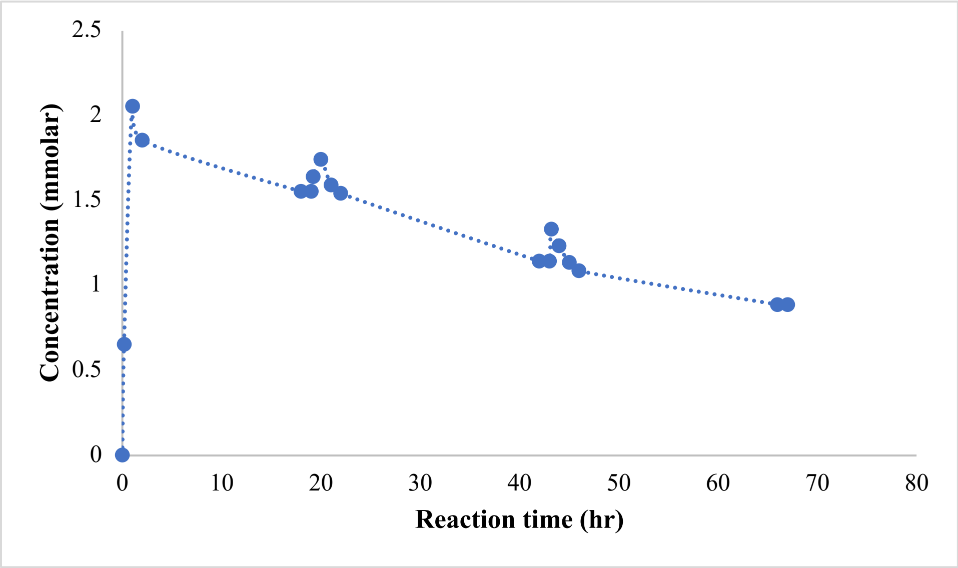

Figure S57. Graphical depiction of the concentration of **2** as a function of reaction time.

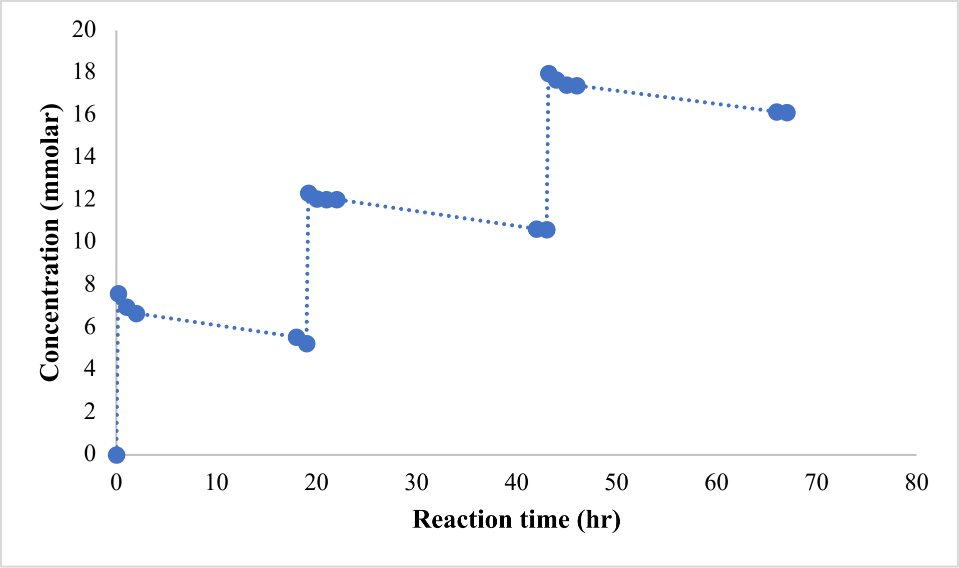

Figure S58. Graphical depiction of the concentration of $ZnEt_2$ as a function of reaction time.

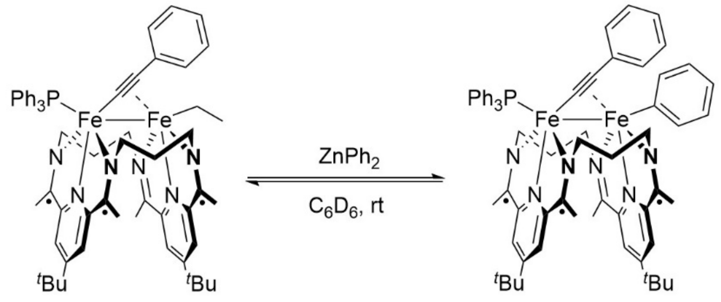

2 $\rightleftharpoons$ **1**^{H,H}

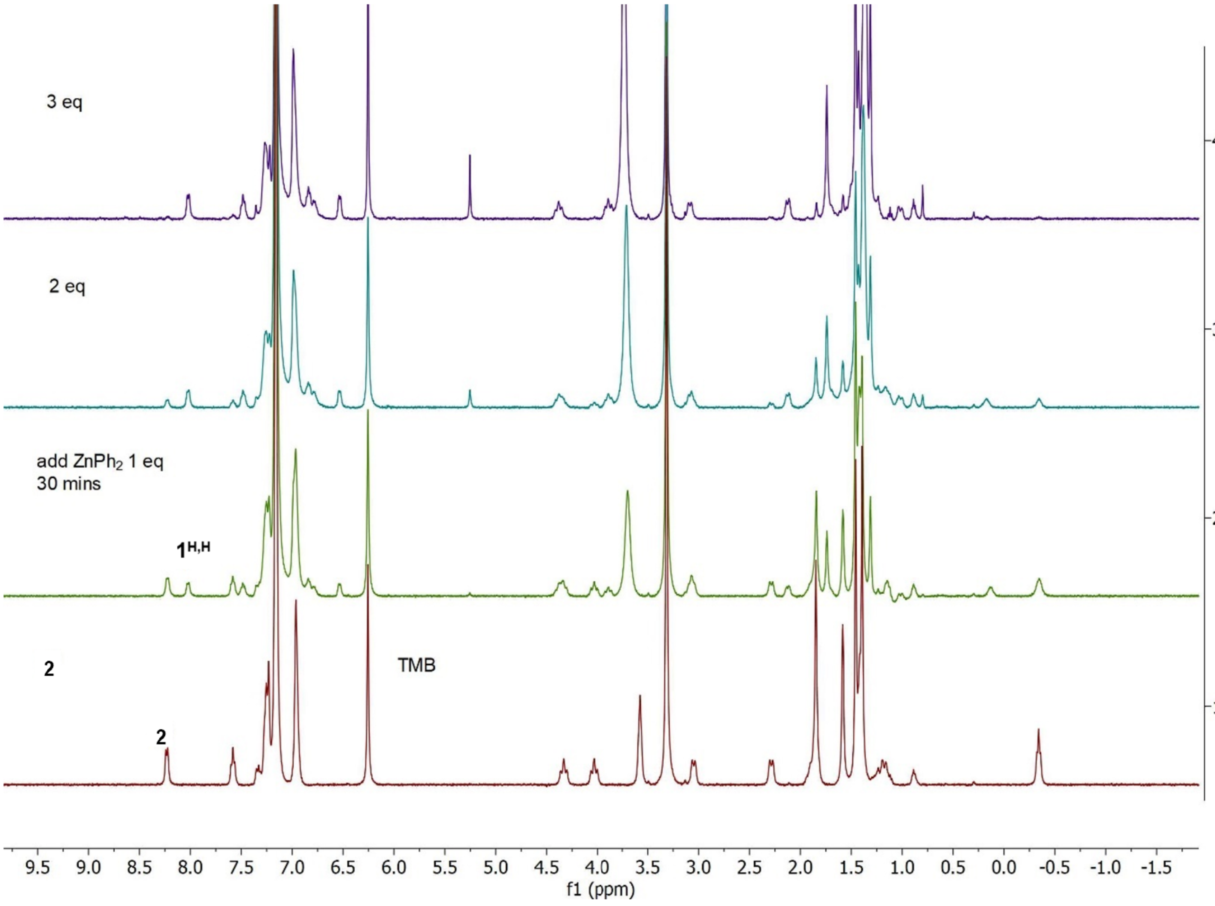

Figure S59. Fe-Zn exchange reaction of **2** with $ZnPh_2$, as monitored by ^1H NMR spectroscopy in C_6D_6 (298 K). The decomposition and **2** and the formation of ethylene appear to be promoted by the presence of the Lewis acid. Monitoring **2** solution at room temperature without the LA indicated a more thermally robust system.

Evaluation of the competence of [4]$^{2+}$ to form PhCCPh on oxidation.

We sought to determine if oxidation of **[4]**$^{2+}$ would yield PhCCPh. Standard analytical techniques were unable to ensure that bulk samples of **[4][PF$_6$]$_2$** were free of the proposed intermediate **[1][PF$_6$]$_2$**. Thus, knowing that the formation of **[4][PF$_6$]$_2$** occurs spontaneously, we performed several reactions in parallel that allowed the 2 e$^-$ oxidation of **1**^{H,H} to stir for varying lengths of time before treatment with O_2. Doing so revealed that longer reaction times correlated with lower yields of PhCCPh, suggesting that oxidation of **[4][PF$_6$]$_2$** is unable to produce PhCCPh under these conditions.

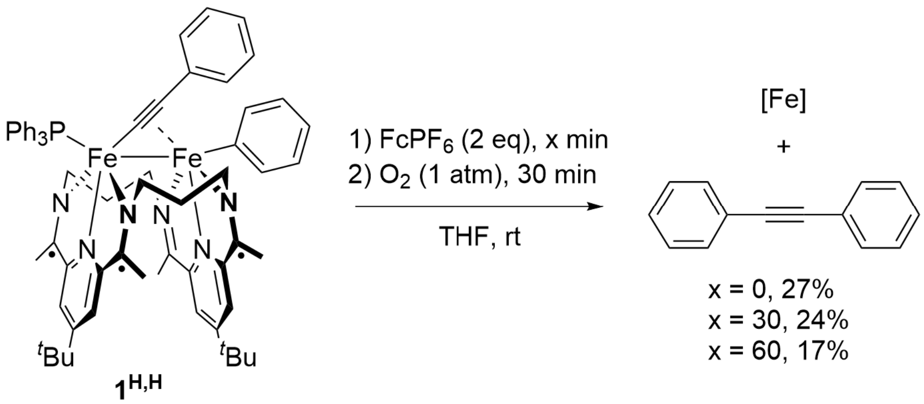

General Procedure: A 20 mL vial compatible with a septa-seal screw cap was charged with **1**^{H,H} (8.0 mg, 7.5 µmol), 5 mL of THF, and a stir bar. To the stirring solution was added $FcPF_6$ (5.0 mg, 15.1 µmol) as a slurry in 1 mL of THF, affording a color change from dark green to reddish-brown. The vial was capped, and the seal was reinforced by tightly taping around the joint with electrical tape. The mixture was allowed to stir for x min (x = 0, 30, 60) before being removed from the glovebox. The headspace within the vial was purged via a needle with O_2 for 5 min, and the resulting brown mixture was to stirred for 30 min. The vial was opened to air and the mixture was filtered through celite, then dried with a steady stream of air. To the remaining gray-brown residue was added 10 mL of hexanes, and the mixture was sonicated for 10 minutes. The mixture was filtered through celite and dried with air flow, affording a yellow oil. The analyte was dissolved in 1.20 mL of hexanes, transferred to a Thermo Scientific 2 mL clear target DP vial with a screw septa cap, and subjected to GC-MS analysis.

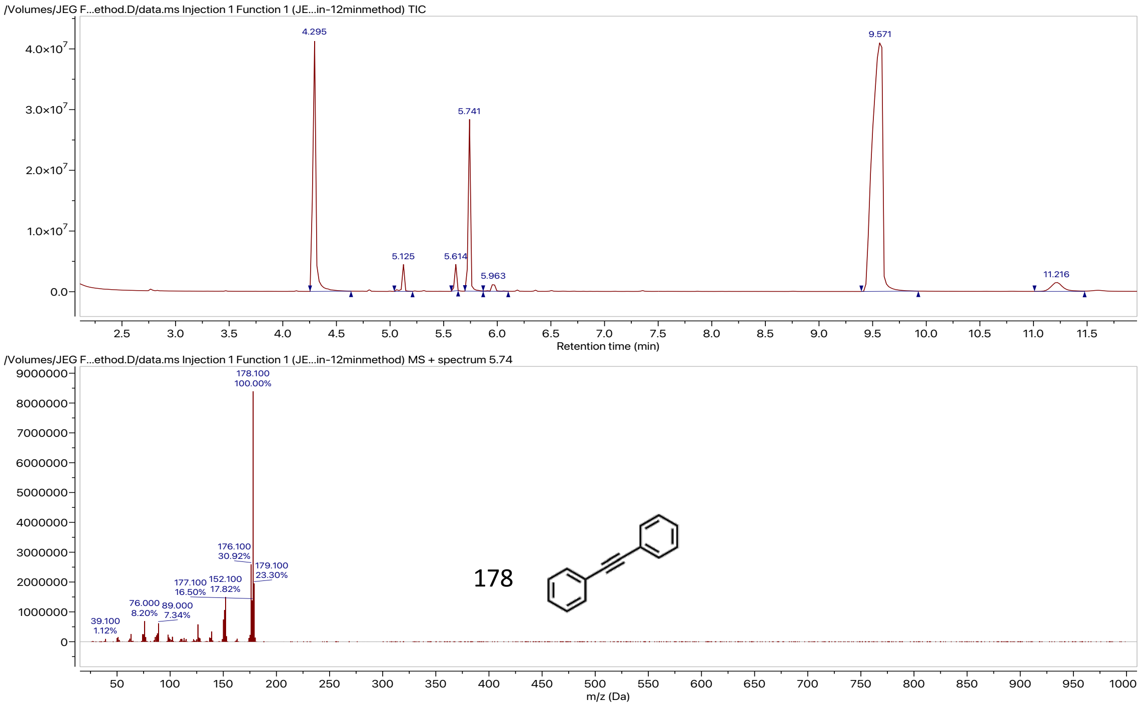

Figure S60. GC-MS data for the oxidatively-induced reductive elimination of PhCCPh from *in situ* generated **[4]**$^{2+}$, x = 0.

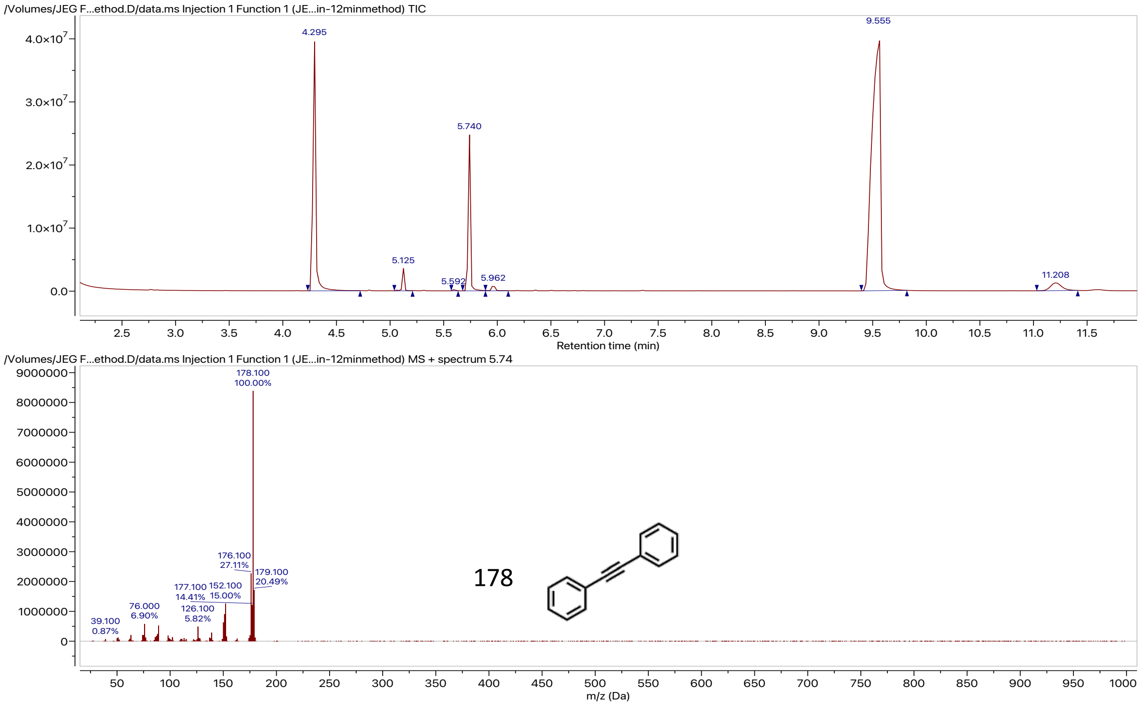

Figure S61. GC-MS data for the oxidatively-induced reductive elimination of PhCCPh from *in situ* generated **[4]**$^{2+}$, x = 30.

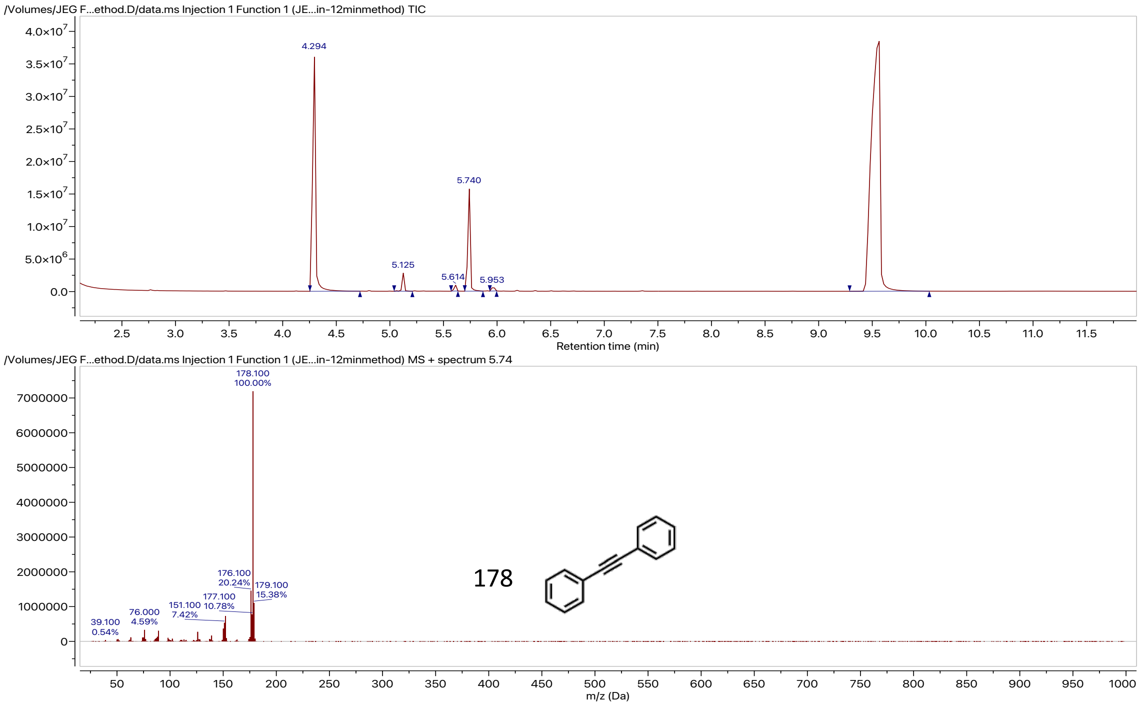

Figure S62. GC-MS data for the oxidatively-induced reductive elimination of PhCCPh from *in situ* generated **[4]**$^{2+}$, x = 60.

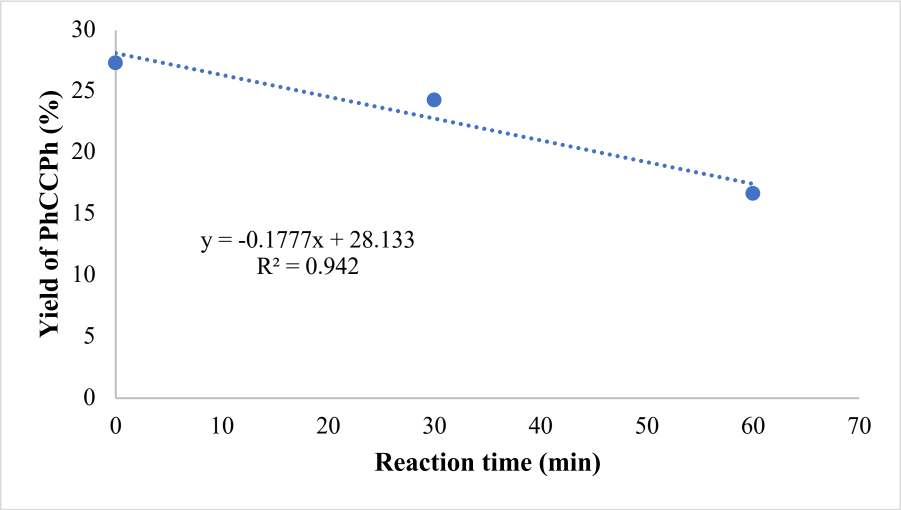

Figure S63. Graphical depiction of the yield of PhCCPh as a function of reaction time.

Investigation of the effect of PPh_3 addition to the Fe-Fe exchange reaction.

In a typical experiment, co-crystallized $\mathbf{1}^{\mathbf{Me,OEt}}$ and $\mathbf{1}^{\mathbf{OEt,Me}}$ (6.4 mg, 0.0057 mmol, 1 equiv) , PPh_3 (1.5 mg, 0.0057 mmol, 1 equiv), and a known amount of 1,2,4,5-tetramethylbenzene (3.0 mg) as internal standard were loaded into a J. Young NMR tube and dissolved with 0.8 mL of THF-d_8. ^1H and $^{31}P\{^1H\}$ NMR spectra were collected, then a THF solution of $ZnCl_2$ (0.1 M, 2.8 μL, 0.05 equiv) was added by a microsyringe. The reaction progress was monitored by ^1H and $^{31}P\{^1H\}$ NMR spectroscopy.

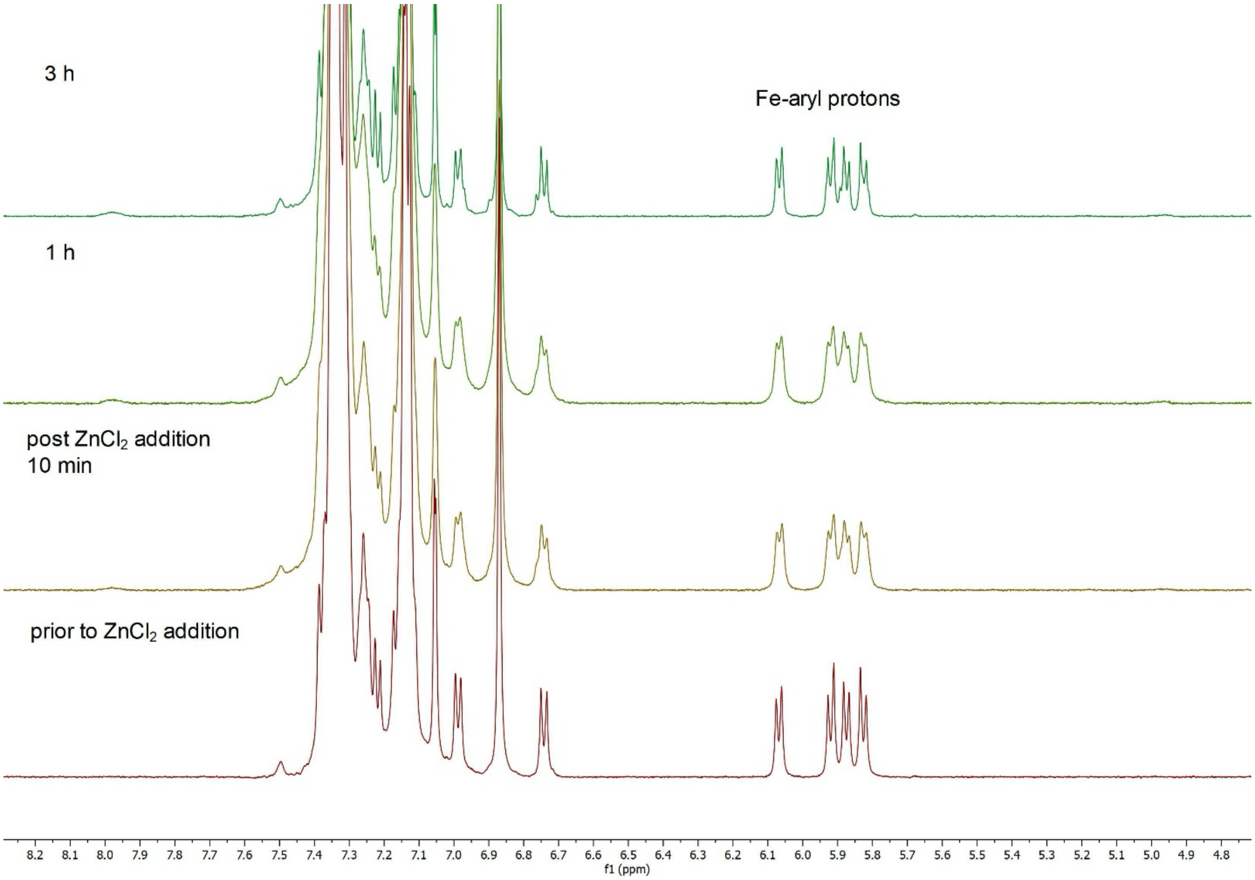

Figure S64. Reaction progress monitored by ^1H NMR spectroscopy in THF-d_8 (500 MHz, 298 K).

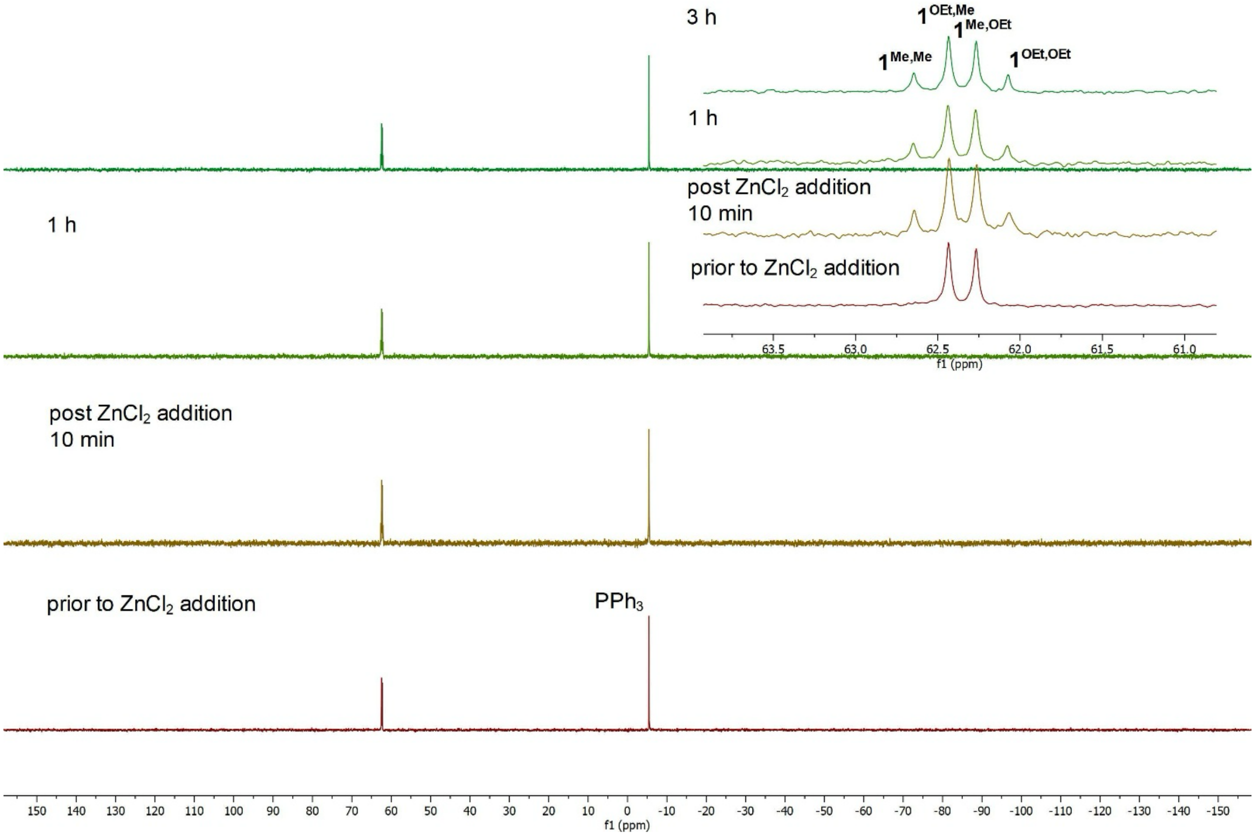

Figure S65. Reaction progress monitored by $^{31}P\{^{1}H\}$ NMR spectroscopy in THF-d_8 (202 MHz, 298 K).

Computational Studies

General procedures. All density functional theory (DFT) calculations were performed using the ORCA program package, version 4.2.1.[13-14] Geometry optimizations and frequency calculations were performed using unrestricted DFT at the B97-D3 level of theory.[15-17] All Fe, N, P, Zn, Al and bound carbon atoms were calculated with def2-TZVP basis sets, due to their proximity to the metal centers, while everything else was calculated with def2-SVP basis sets. Def2-TZVP/J auxiliary basis sets were used on all atoms.[18-19] Calculations were started from crystallographic coordinates, when available, and all Fe_2 complexes were truncated by substitution of the *tert*-butyl groups for hydrogens ($^3PDI_2$') to minimize computational expense. The Conductor-like Polarizable Continuum solvation model (cpcmc) was employed for all structures, with the dielectric constant of THF.

Lewis Acid Screen. The thermodynamics of aryl group abstraction from (**1$^{H,H'}$**) with various Lewis acids was investigated. The energies of {**1$^{H,H'}$** + LA + PPh_3} were compared to those of the proposed aryl group abstraction products, {**[IM']$^+$** + **[PhLA]$^-$** + PPh_3}, and their phosphine adducts (**[3']$^+$** + **[PhLA]$^-$**). The results are given in the table below. The level of discrete solvation of each Lewis acid by thf was evaluated to determine the most favorable solvate at the level of theory used in this study.

	Acceptor Number	Relative Free Energies (kcal/mol)		
Lewis Acids		**1$^{H,H'}$** + LA + PPh_3	**[IM']$^+$** + **[PhLA]$^-$** + PPh_3	**[3']$^+$** + **[PhLA]$^-$**
$AlCl_3$	77	0	-28.1	-44.0
$ZnCl_2(thf)_2$	51	0	4.4	-11.6
$ZnEt_2$	14	0	53.9	37.9

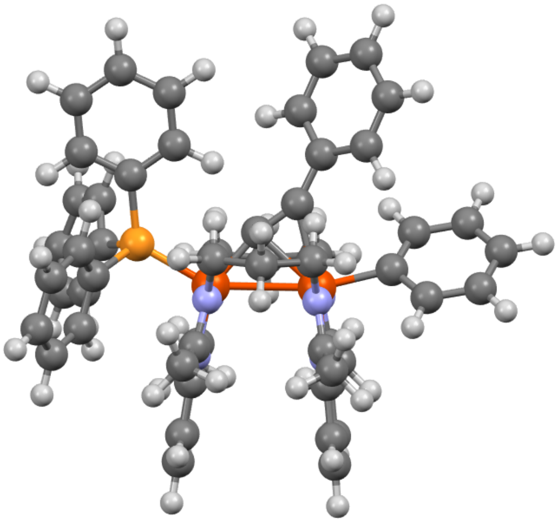

Figure S66. DFT-optimized structure of complex **1$^{H,H'}$**, [($^3PDI_2$')Fe_2(μ-CCPh)(Ph)(PPh_3)].

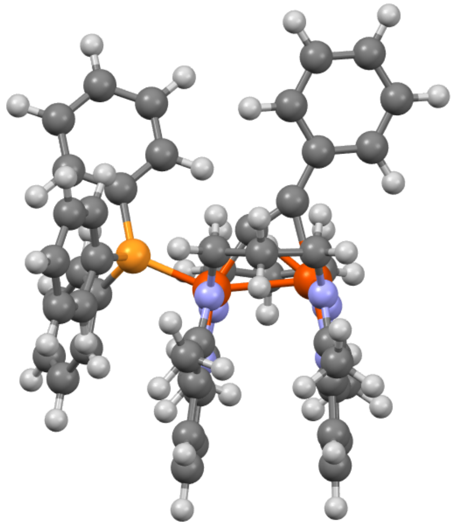

Figure S67. DFT-optimized structure of complex **[IM']**$^{+}$, $[(^{3}PDI_2')Fe_2(\mu\text{-}CCPh)(PPh_3)]^{+}$.

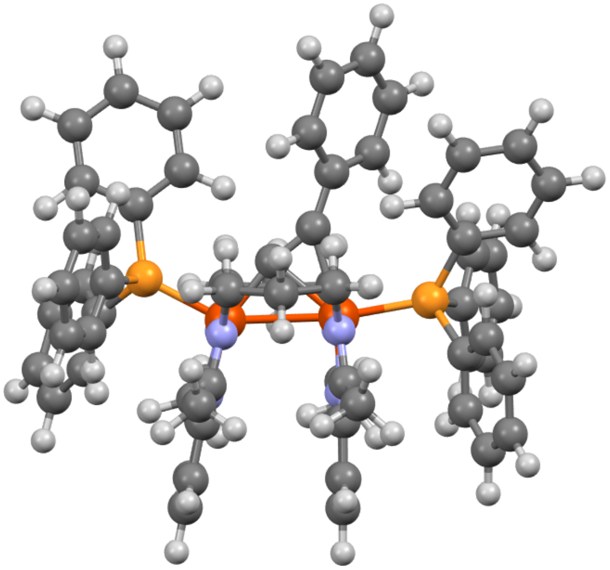

Figure S68. DFT-optimized structure of complex **[3']**$^{+}$, $[(^{3}PDI_2')Fe_2(\mu\text{-}CCPh)(PPh_3)_2]^{+}$.

Alternate Pathways. Potential alternative mechanisms for Fe-LA exchange were evaluated. Dissociation of triphenylphosphine from $\mathbf{1}^{\mathbf{H,H'}}$ to form [(^{3}PDI$_2$')Fe_2(μ-CCPh)(Ph)] (**A**) was found to require an input of 24.4 kcal/mol. Further intermediates were all found to be unreasonably high in energy, including a $ZnCl_2$ adduct (**B**, +207.4 kcal/mol relative to $\mathbf{1}^{\mathbf{H,H'}}$), a chloride-bound ate-complex (**C**, +223.8 kcal/mol relative to a tolyl derivative of $\mathbf{1}^{\mathbf{H,H'}}$, $\mathbf{1}^{\mathbf{H,Me'}}$), and a diaryl ate-complex (**D**, +76.4 kcal/mol relative to $\mathbf{1}^{\mathbf{H,H'}}$).

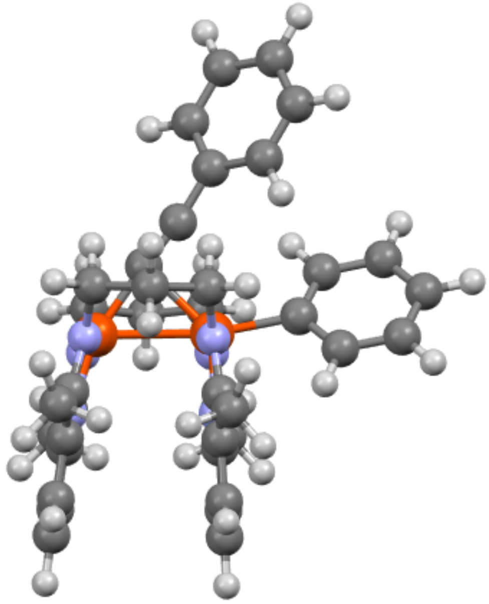

Figure S69. DFT-optimized structure of complex **A**, [(^{3}PDI$_2$')Fe_2(μ-CCPh)Ph].

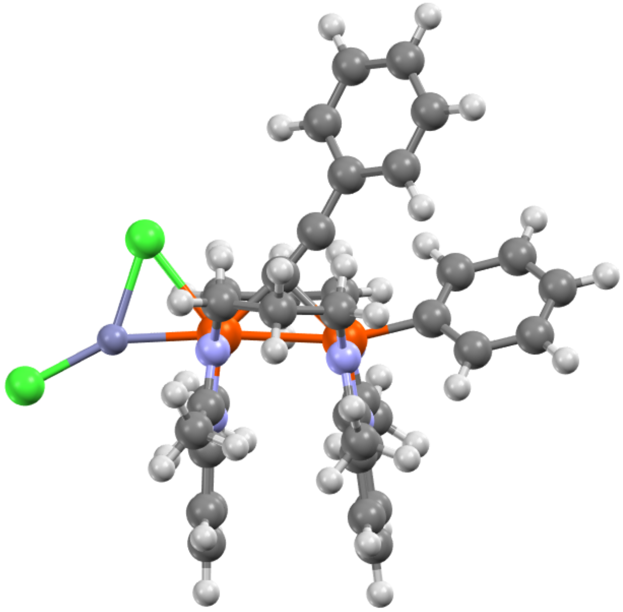

Figure S70. DFT-optimized structure of complex **B**, [(^{3}PDI$_2$')Fe_2(μ-CCPh)(Ph)($ZnCl_2$)]$^+$.

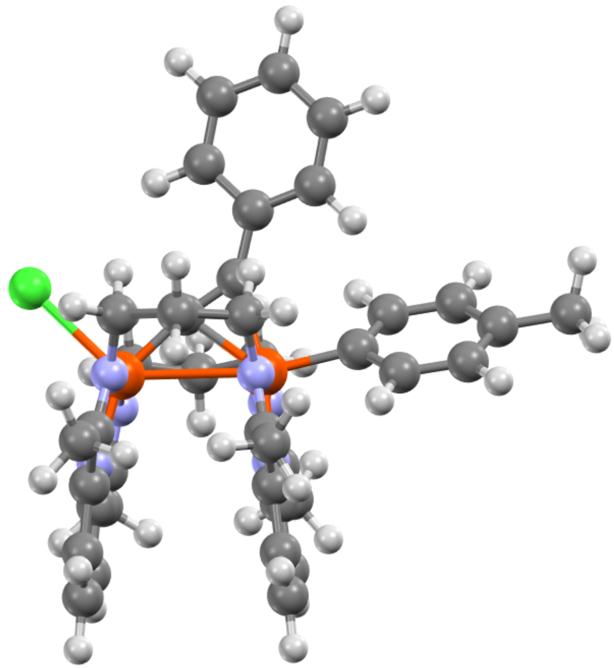

Figure S71. DFT-optimized structure of complex **C**, [(^{3}PDI$_2$')Fe$_2$(μ-CCPh)(4-MePh)(Cl)].

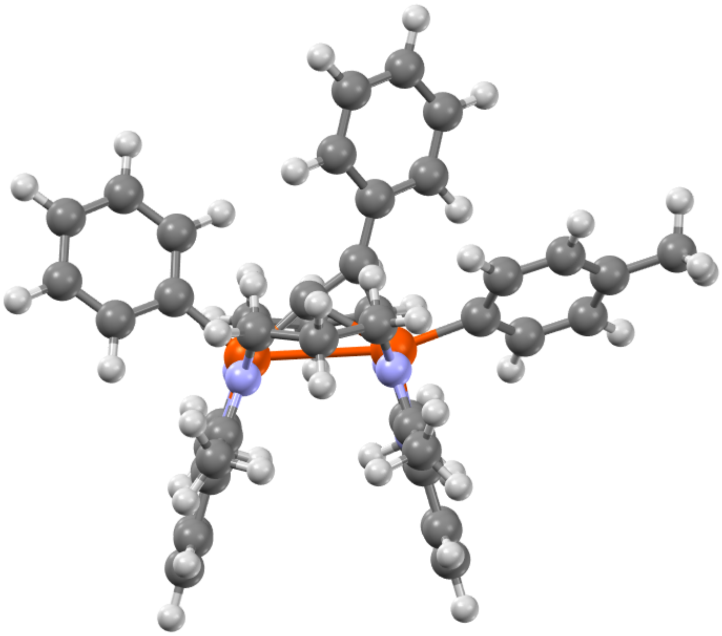

Figure S72. DFT-optimized structure of complex **D**, [(^{3}PDI$_2$')Fe$_2$(μ-CCPh)(Ph)(4-MePh)].

DFT Coordinates, Energies and Spin States

[(^{3}PDI$_2$')Fe$_2$(μ-CCPh)(Ph)(PPh$_3$)] (**1$^{H,H'}$**)

G = -5361.83507476 E$_h$, Singlet

This species has 1 imaginary frequency (-42.66 cm^{-1}), corresponding to a phenyl group rotation

Fe	3.53212827175952	10.02467225766170	8.66696055316902
Fe	5.80818093886833	10.76177754540644	9.96924994914797
C	2.90437177951509	10.16773646336848	10.79616221564168
C	4.14136052525427	10.39449766473077	10.63214872161100
C	1.70209558503914	9.41349299466287	8.17625475002549
P	6.88743746071847	11.35307427389361	11.77605489463607
N	4.10001315688327	8.16347658607974	8.64549573504401
N	4.26128429479762	9.96823924138890	6.99811376715758
N	3.15943204915053	11.82915547915749	8.13742398301021
N	5.51691247575240	12.61753436919895	9.49258066047773
N	6.99496716078921	10.89711176894802	8.55886069687547
N	6.50323871220083	8.96129687877315	9.97446928522556
C	0.44827872494872	10.42302591205670	11.16715817186825
C	-0.66520491529646	10.31142912713822	12.00664939358095
C	-0.53492964509016	9.80051242848817	13.31133233623913
C	0.73503389140189	9.39480538777974	13.76223084368702
C	1.85281245598656	9.50010253806921	12.92657085875641
C	1.74291898861760	10.03227286787167	11.60853009121210
C	0.99886721133076	8.40666685259518	8.86835963182798
C	-0.26046266675944	7.94901656204562	8.44949781648642
C	-0.86835007164401	8.48676983633488	7.30271478647699
C	-0.18288176015788	9.48182668890001	6.58845044160339
C	1.08069831009738	9.92613764835381	7.02000023258339
C	4.68522624914358	11.61535740089417	13.52726252882284
C	4.06907489738240	11.89612986495539	14.75850352966757
C	4.84087724991982	12.31279630482237	15.85242000723646
C	6.23493876546942	12.44536770752369	15.71384641572618
C	6.84870262104662	12.16230484104623	14.48764647918341
C	6.07599541914331	11.74655138772351	13.37944016812984
C	7.90910385711694	9.31453875015647	13.45389111811395
C	8.75945200773829	8.24622686360126	13.77459243394121
C	9.84854617537430	7.93425015783394	12.94648004150081
C	10.07610621942007	8.70171996734168	11.79283004267867
C	9.22608162653201	9.76818191730216	11.47133422897008
C	8.13462335686317	10.10221360356122	12.30377990356301
C	8.79601225357402	12.93871823182208	10.40074337917150
C	9.41006105274114	14.14071976416094	10.02510570376637
C	9.05824738241427	15.34386468605063	10.65556807787784
C	8.08337154501700	15.32835210046278	11.66484322476239
C	7.47852835310047	14.12252013265969	12.04976672111363

C	7.83586671600055	12.90117391886436	11.43646203815409
C	4.99671998241455	6.31898493630151	7.16766253368929
C	3.20785045966045	13.45249961315264	6.19654409800502
C	6.36091291514868	14.54079850384870	8.10052139263500
C	8.22514936715728	7.40243148953367	8.98959201724758
C	6.11523260098932	7.95102755542350	10.96280244642081
C	5.27779317657722	6.80085821828682	10.39224778662823
C	3.94701745831511	7.23673172674170	9.76869805423463
C	4.62607784702675	7.73643570061135	7.50446373532795
C	4.78911249045784	8.79199135216810	6.53015204382479
C	5.39034197461591	8.74583393894248	5.26077064902449
C	5.41949886325264	9.91277230034638	4.47540897881505
C	4.82069911542255	11.09359894254869	4.94574798412318
C	4.22533946834182	11.10017224133697	6.22028576907886
C	3.52453447193127	12.16245961971117	6.89985110300139
C	2.47728167016510	12.81024386595033	8.98219221974892
C	3.38417916106057	13.96295704559674	9.43170987416089
C	4.61620571759147	13.51226180307995	10.22596753097976
C	6.27195166661914	13.09863922203494	8.50854202546130
C	7.09951472226367	12.09045887801651	7.88875081260534
C	7.94941813743253	12.19310480588637	6.77526972261210
C	8.68278200981971	11.06530685210543	6.36768388383083
C	8.57461719877957	9.85508805021522	7.07814060809441
C	7.71500991920485	9.78828045343082	8.18588391749209
C	7.44503840439554	8.68310776301877	9.07623036442947
H	9.33912339843529	11.12733852710925	5.49263128089969
H	5.90122339210837	9.89792720253847	3.49141750780946
H	0.33481582155716	10.80337513667890	10.14874390711301
H	-1.65082284390885	10.62274994530848	11.63736543221372
H	-1.41135273021605	9.71183325708872	13.96455791936228
H	0.85333801049761	8.98616502814057	14.77430198768176
H	2.83763048292921	9.17343916176252	13.27856931077413
H	1.41793013497540	7.98100268101512	9.78279374886631
H	-0.77475115859202	7.17182562742179	9.03199902601962
H	-1.85463743603030	8.13624656194001	6.97254759345066
H	-0.63187811591775	9.91746645090572	5.68438661599029
H	1.58025691224193	10.69941034709158	6.42538143839159
H	4.09174748977162	11.28081862965992	12.67237996688171
H	2.98346711577225	11.77857761897127	14.85597054841844
H	4.36204553327411	12.53170539841081	16.81445301495253
H	6.84466350461162	12.76661242994969	16.56694363926681
H	7.93538192447187	12.26115316326043	14.38925449816683
H	7.05388425516346	9.52599592348172	14.10191555311448
H	8.56321551534460	7.65181179291475	14.67511401028217

H	10.51181456859025	7.09713635996502	13.19439895958606
H	10.91426555335760	8.46256101716612	11.12757729901512
H	9.41257314364188	10.32231410075323	10.55066979416804
H	9.04939253692033	12.03192373600156	9.85058292187912
H	10.15053878913476	14.13544060757433	9.21641902419694
H	9.52900430658073	16.28691217674892	10.35351498278116
H	7.78742830920625	16.26119859043677	12.16005943402629
H	6.71298563905296	14.13971707544827	12.83005892401095
H	4.48879495634177	5.58855147359384	7.81504271016939
H	4.71879343911409	6.09567548600392	6.12292171381405
H	6.08546778057231	6.14039184154139	7.24917479250314
H	2.34942202610121	13.97175080521720	6.64914167963646
H	4.06174939675644	14.15617518099422	6.19769336553174
H	2.96265779156532	13.25508808315424	5.13838192452008
H	6.16546129823220	15.21450605894719	8.94944641030776
H	7.37228761595240	14.76623673656466	7.72597771095912
H	5.64643270326421	14.79934179647230	7.29725799894059
H	8.29501470405025	6.89935937987587	9.96601202512078
H	7.78038220008949	6.68632582531884	8.27355941897812
H	9.25298282174624	7.60625761545260	8.64623297271635
H	7.01610673383473	7.53395504333682	11.44748175626204
H	5.53661138235189	8.47199524502735	11.73867802663732
H	5.04905872223888	6.11126428430395	11.22701481445653
H	5.87256730056914	6.22139045407028	9.66841484437316
H	3.35403161724642	7.75662860464684	10.53361334816732
H	3.37616881036695	6.34281997592251	9.45339036858791
H	5.83665016016583	7.81450908021006	4.89854003828212
H	4.82302083584032	12.00427978483991	4.33860817733136
H	1.59187886644906	13.21739834610969	8.45672680168802
H	2.11794735857844	12.27038596341022	9.86729855785586
H	2.79027804223288	14.62811506278625	10.08702873924758
H	3.69045484216657	14.57387559340590	8.56763171992801
H	4.28407191288973	12.96907615483614	11.11964621164307
H	5.17398473245081	14.40342223358899	10.56881427589379
H	8.02637392863666	13.13828971234824	6.22894042331369
H	9.13995041941323	8.97121385909434	6.76651899801358

[(^{3}PDI$_2$')Fe$_2$(μ-CCPh)(PPh$_3$)]$^+$ (**[IM']**$^+$)
G = -5130.42346212 E$_h$, Quintet
This species has 2 imaginary frequencies (-48.93 and -79.17 cm^{-1}), both corresponding to imine methyl rotations

Fe	5.75310729455607	10.79658336918619	10.04657021865202
Fe	3.51210805562755	10.10873018500800	8.93494099483346
C	4.13083743628696	10.46801155751040	10.83468471503673

C	2.88976313217391	10.16247179580287	10.93378876303433
P	6.94028400272045	11.39958349726906	11.79741383011243
N	6.38809153998073	8.95743644399882	10.03385473039375
N	6.92471721272430	10.89139578537479	8.61781268769184
N	5.46470028153424	12.65123286431747	9.52113656898729
N	3.01119731044684	12.04568464990385	8.08166593746522
N	4.17867230650117	10.02567464948859	7.01822940382547
N	4.02027742591348	8.01781941734350	8.60176683356902
C	7.34782064588886	8.67316403077876	9.15785493931015
C	7.64537511522906	9.77563121273758	8.27136469419357
C	8.52790300400451	9.82571208622290	7.18157745726560
C	8.65570852223026	11.02519270982003	6.45754777992674
C	7.92550221741128	12.16228625840290	6.84268791370905
C	7.05783781463270	12.07792779485666	7.94269423362014
C	6.23976498781899	13.10804947101215	8.54126154690798
C	4.56696755049171	13.56960184710533	10.23492522132584
C	3.39551282265733	14.12316907747322	9.41035342531029
C	2.38715141897876	13.07815980332754	8.90490955217935
C	3.43907968955252	12.27434351631770	6.87137198633713
C	4.17705362870129	11.15591773293747	6.26281604300404
C	4.85164714482867	11.19751944289929	5.03335981595315
C	5.51477258619898	10.04634241027878	4.57953743636471
C	5.44612369695701	8.86758620153894	5.33968695853338
C	4.75578214709806	8.88293091968790	6.56058064680706
C	4.58846828061005	7.73275606001930	7.46390551806814
C	3.87618774027706	7.04361064003345	9.68048980900661
C	5.20531417016151	6.73408778759758	10.38825198372162
C	5.94783189989645	7.93504531634273	10.99433288779332
C	8.12299206229372	7.38833292078296	9.08965336705697
C	6.37191402607700	14.54482800970791	8.12457385057572
C	3.23213015920218	13.53592470871983	6.07965671781567
C	5.05263763772022	6.37374182351579	7.01779198621756
C	7.90850289468092	12.90943008267353	11.38138739989047
C	7.58882560378906	14.15186504656728	11.97384069416607
C	8.21230545085354	15.33390575294150	11.54831733087581
C	9.16712785769128	15.30313007238387	10.52015289742517
C	9.48021373769528	14.07907736025310	9.91090744151740
C	8.84661130940442	12.89982327029952	10.32547041765846
C	8.13899761967195	10.09875031417826	12.29565478079591
C	9.23915712812461	9.75108801548263	11.48198406437540
C	10.04810284591334	8.65206318381798	11.80168304873722
C	9.76797545792181	7.86805906439390	12.93175842980640
C	8.66805027171617	8.19459627636947	13.74006607385360
C	7.85865805629561	9.29463457113340	13.42273915084750

C	6.19341678798040	11.85038601766274	13.41275028286180
C	7.03419428952974	12.22492030983188	14.48556497760764
C	6.48724602832804	12.56542812533937	15.72841523212151
C	5.09378963956913	12.53491773097538	15.91896672422628
C	4.25407164293058	12.16326208242864	14.85941060870971
C	4.80334505236176	11.82387166073939	13.61172894758651
C	1.70641847939377	9.88454824119787	11.68974243533847
C	1.78214109240917	9.60519641599732	13.08194765514076
C	0.62368862421168	9.33829541326832	13.82031335875235
C	-0.63670943696412	9.33914878155641	13.19516741873556
C	-0.72622927945502	9.60691356434748	11.81791960909623
C	0.42884312809015	9.87299685696706	11.07283735520926
H	9.10011867748846	8.93775907686084	6.89647648247593
H	8.02652618826961	13.10418529510332	6.29530600602169
H	5.15695545347074	14.41803953575075	10.62889272354823
H	4.17247656289012	13.02267504254677	11.10013132251488
H	3.77136801199395	14.71642318017186	8.56153390708375
H	2.84467762920825	14.82935515556482	10.05899863195311
H	1.92513561586126	12.56882290338554	9.76636036105894
H	1.57862737348096	13.59536337815575	8.35387897284431
H	4.86770188701550	12.11591086381062	4.43988097381548
H	5.92967236249563	7.95255852194798	4.98645568625527
H	3.44135095061964	6.09702241302745	9.30682375286150
H	3.16393828669560	7.46860054660125	10.40579814920626
H	5.86976117654400	6.18574285140218	9.70169673294020
H	4.98283274032259	6.03863516853339	11.21889333331330
H	5.29950406846299	8.44067329979551	11.72337050857512
H	6.82588158487913	7.55708350078385	11.54766049509849
H	9.15259316342805	7.58764730974620	8.75130953882551
H	7.68258213440646	6.66362495561183	8.38084533787194
H	8.18646053711002	6.89971253416163	10.07355615432038
H	5.69625057938470	14.81364460741609	7.29251278381439
H	7.40149286628493	14.74312987374111	7.78708699979116
H	6.16262586242608	15.22951361965944	8.96067729287408
H	2.80703448352532	13.29463989196335	5.08967109962481
H	4.19205818275476	14.04930504593371	5.89393044368182
H	2.55741582905016	14.24190809916532	6.58273680950157
H	6.15081264374748	6.35070471654636	6.90222058594113
H	4.62590178520226	6.13459176279879	6.02794937266176
H	4.76476186307001	5.57963259092454	7.72002386281889
H	6.84331919922420	14.20315856115135	12.77181357303495
H	7.94806077620140	16.28424116170190	12.02724876958424
H	9.65424977690888	16.22758506147591	10.18881643598703
H	10.20568784131694	14.03818514269633	9.09009757864712

H	9.07354267368920	11.97511706002008	9.79425488002293
H	9.47395127351272	10.32430934743483	10.58443441383907
H	10.89789054584772	8.40396052062094	11.15508919953608
H	10.40045424679207	7.00742926306155	13.17860476337598
H	8.43297393915549	7.58885356185081	14.62322195776215
H	6.99534324915141	9.52063731252816	14.05522252366720
H	8.12053067636514	12.24693489807618	14.34612371879470
H	7.14920180848664	12.85407870084937	16.55335459256630
H	4.66745411752265	12.79890898518845	16.89394949907202
H	3.16711061481809	12.13057597303391	14.99853021406206
H	4.15287761233842	11.52342061385188	12.78651252613874
H	2.76270611882549	9.59841708358644	13.56990188688013
H	0.70276080815470	9.12571723118092	14.89366389738205
H	-1.54177791376526	9.12964259212333	13.77712908800272
H	-1.70445798836296	9.60668106288438	11.32191141077659
H	0.36068322409056	10.08221691939297	9.99829923351250
H	6.06558811030678	10.06246243981936	3.63378076768134
H	9.33075491630064	11.07415528162791	5.59668511563334

[(^{3}PDI$_2$')Fe$_2$(μ-CCPh)(PPh$_3$)$_2$]$^+$ (**[3']**$^+$)
G = -6165.60523918 E_h, Triplet

Fe	3.55032376237293	9.95221163804589	8.97157832468912
Fe	6.11884063245464	10.87089924701274	10.03846363045767
C	3.24376196539393	10.18508865562404	10.91650461481410
C	4.48162410613476	10.52693876533705	10.69359982583215
P	1.38647841783418	9.35688072918904	8.31470434402763
P	7.35127510447090	11.39913756960370	11.78404273012029
N	4.21105783880447	8.08912332061726	8.95931187288708
N	4.34414545440393	9.86660883053691	7.26049484639499
N	3.29349004400661	11.81757349504932	8.34133392372751
N	5.81518026220665	12.71653964973426	9.53310439678566
N	7.26897282287427	10.98440745672626	8.58445943481390
N	6.78977996567308	9.04856126095557	10.02253931113711
C	2.12198261611994	11.25817541964065	12.84348361052012
C	1.17107531814179	11.25074862323052	13.87089169134626
C	0.35819268396424	10.12333781762469	14.07787957696533
C	0.51010171291679	9.00117890509672	13.24628161502811
C	1.45023295118343	9.01265376680849	12.21182175723185
C	2.27455969068744	10.13942249364098	11.98426219759072
C	5.44460683981279	10.73801535108746	13.75299497709829
C	4.96266842272527	10.62384480443655	15.06707908376016
C	5.76542007183035	11.02434915630733	16.14475536493130
C	7.05238257956636	11.54030178005638	15.90464489680144
C	7.53069215177302	11.65621152530925	14.59373311801034

C	6.72592053582874	11.25543742572242	13.50315779552047
C	9.08242467028068	9.42107406038298	12.85629978651553
C	10.16795012249546	8.53417140584363	12.80995532801667
C	11.07102469875548	8.57124580473642	11.73581452486184
C	10.86942640674954	9.49841283975214	10.70209894902183
C	9.77863945986737	10.37665471125862	10.74141318951385
C	8.87734045677642	10.37143278524579	11.83078728348983
C	8.79834703232591	13.59194037599270	10.70840036986343
C	9.10507421461020	14.95224194780132	10.56998495665295
C	8.51613943170049	15.90368247206652	11.41729699627027
C	7.61468154660769	15.47843285864290	12.40602340839267
C	7.31055312472424	14.11716702508690	12.54937017558574
C	7.90901690338871	13.14851552003377	11.71313316594840
C	5.02373841098272	6.20823973785506	7.49304373689373
C	3.39778193944759	13.34575269329350	6.34084683589044
C	6.61591410787745	14.63168476544780	8.10597945297189
C	8.48256521403130	7.48023090253230	9.01748851183607
C	6.47675375656301	8.07458395172089	11.07431014929683
C	5.63130769478015	6.87619330429099	10.63055893594324
C	4.21962048191185	7.21513954082061	10.13713209141883
C	4.67865900919687	7.63427225100151	7.80933035199461
C	4.82576801256120	8.66827551136150	6.80907300555296
C	5.40296672731676	8.57421012791105	5.53234320464637
C	5.48763255050156	9.72777219259357	4.73388966097819
C	4.94833255759093	10.93790749300103	5.19629896266126
C	4.35155953957358	10.97797843041304	6.46665259319221
C	3.66952297305346	12.08348587403426	7.10414633242112
C	2.70064399000892	12.86838006330850	9.17398080932295
C	3.65557447880735	14.01992530065122	9.51522354386366
C	4.92471231991293	13.61416851986912	10.27500495862635
C	6.55714555527011	13.19319590174699	8.53368476467515
C	7.37366551668152	12.17852349937016	7.91021720727958
C	8.20552315559207	12.26762077298827	6.78323480890463
C	8.90613977171773	11.12437207775611	6.35597928992386
C	8.78585900191471	9.91356346742318	7.06240934366120
C	7.95565689623664	9.85947250650470	8.19272011817221
C	7.70579614928823	8.76256598187578	9.09825933657637
H	9.54718310920548	11.17706137822776	5.46940735116579
H	5.96111529874032	9.67855462189727	3.74796052973187
H	2.75844959122833	12.13578550507837	12.68794622643121
H	1.06522646982321	12.13044692655511	14.51754980349647
H	-0.38930312097954	10.12041486591865	14.87979679040177
H	-0.12028699680578	8.11671416415234	13.39520404553904
H	1.55016031237154	8.14203224721634	11.55960849756813

H	4.82452991630128	10.43355968255826	12.90734170810127
H	3.95446627327994	10.22948555125398	15.23871855656958
H	5.39251331364944	10.93853894772118	17.17233570345919
H	7.68372471114988	11.85561085776873	16.74383102126403
H	8.53274075253059	12.06088404792094	14.41460006255392
H	8.38672222280797	9.36309804167380	13.69759094924991
H	10.30563049318422	7.80930798332627	13.62125003095231
H	11.91889788413222	7.87721168710512	11.70034716402611
H	11.55174518228709	9.52797013858710	9.84438015927947
H	9.62271933926049	11.04895292800461	9.89732417122825
H	9.25344416996108	12.88342739938493	10.01574919040454
H	9.80018281683901	15.26897433603143	9.78348203876064
H	8.75407691598905	16.96789452447609	11.30496427922890
H	7.14132341953618	16.20967273275552	13.07212701669499
H	6.59260549731193	13.81019722409408	13.31521283512453
H	4.57560810180626	5.50393804099043	8.20771279811134
H	4.64509432644551	5.95328289882016	6.48865435453194
H	6.11600637316245	6.04066838476422	7.47829853036360
H	2.65435049400806	13.98606459723698	6.83432878809917
H	4.31938333375482	13.93502834627753	6.18742819201903
H	3.00892270923992	13.08844396845065	5.34059394714873
H	6.51212685903757	15.31151930437807	8.96668718851283
H	7.58387105739688	14.85055015996531	7.62854055659389
H	5.82401957609436	14.89323484663608	7.37999053947677
H	8.71573415128616	7.08801569133991	10.02057271546414
H	7.93754697431580	6.68932999007371	8.47009992135389
H	9.43891408216678	7.64138168747466	8.49677494781550
H	7.41610741285417	7.69918578402279	11.51958069815715
H	5.94163667856225	8.61687944389543	11.86493611887200
H	5.51860993183328	6.21182682531387	11.50776748433875
H	6.17112829280316	6.28830353992088	9.87218172339703
H	3.66854607423026	7.73152089776589	10.93181839072725
H	3.68044507690986	6.27795393347292	9.91414358083119
H	5.79384312112753	7.61613000907676	5.17806996175336
H	4.97541240938365	11.83862095785005	4.57704259281489
H	1.79383662739687	13.26913676200245	8.68225712213922
H	2.38482933745535	12.39189367201449	10.10988243580440
H	3.09574037397282	14.72379830684615	10.15919689032022
H	3.92411138415688	14.58494954018375	8.60965677556503
H	4.64871660843545	13.09545567256681	11.20384231523344
H	5.46907197812957	14.53036141029748	10.56457258873804
H	8.29579498385031	13.21326445724328	6.23982128706817
H	9.32863695109560	9.02144128939264	6.73535777015022
C	0.91045215470777	10.28524537094868	6.80192184468785

C	1.64359255176020	10.13610087373361	5.60232684225149
C	1.35209215031056	10.91764216725560	4.47582313062291
C	0.34143449615254	11.88851631068457	4.52950961520314
C	-0.38105546841904	12.06239702044439	5.72097578633509
C	-0.10324539877394	11.26879896409883	6.84285334994141
H	2.45111568664892	9.40616557865775	5.53578952669861
H	1.93547543258541	10.77454177598928	3.55887597670869
H	0.12053087509189	12.50729810599045	3.65207631453155
H	-1.17532763621751	12.81622277236031	5.77839151226116
H	-0.68627593572716	11.41657005960337	7.75593683660634
C	-0.08061299326471	9.60289909633968	9.38714646737894
C	-1.16071755483674	8.69816655162149	9.41629997093818
C	-2.26517480266273	8.94020656261852	10.24702420635607
C	-2.31440814465661	10.09616304873379	11.04217218522826
C	-1.25542811974783	11.01552640131114	10.99719459214767
C	-0.14690131451466	10.76526747075873	10.18039945229563
H	-1.14393593484388	7.80032134452110	8.79158632889929
H	-3.09432655146161	8.22285132015269	10.26674787663515
H	-3.17594524829346	10.27901547306815	11.69505694016841
H	-1.27769144509004	11.91840266876704	11.61705265220378
H	0.68018906534849	11.47429364982851	10.16691741736881
C	1.28013830566892	7.57672893173159	7.87164396941948
C	1.29975644695585	7.07483864986931	6.55538648727226
C	1.33590364775905	5.69129302777853	6.31530268070923
C	1.33727103819308	4.78322833336258	7.38310645296212
C	1.29093473522639	5.27122971745911	8.70011511903833
C	1.27035571685883	6.64979893058444	8.93826352854638
H	1.26726241990895	7.75302476331344	5.70103699159514
H	1.35456761690341	5.32664249635735	5.28141602982572
H	1.36484776972956	3.70396838809119	7.19362325048105
H	1.27575344996800	4.57593105400042	9.54764610503474
H	1.23643630572774	7.01157254795753	9.96921313166995

[(^{3}PDI$_2$')Fe$_2$(μ-CCPh)(Ph)], (**A**)
G = -4326.6316951 E$_h$, Singlet
This structure has one imaginary frequency (-6.46 cm^{-1}) from an imine methyl rotation.

Fe	3.49099227225670	10.00162713276617	8.61375067877356
Fe	5.70279921665881	10.73114806628827	9.78569062240826
C	2.92727005962313	10.24013937777108	11.14590380223288
C	4.05403270440278	10.42547154281117	10.63701352792641
C	1.68221184272323	9.42499185168489	8.06315321763762
N	4.09054286342571	8.15948943770209	8.63115353282896
N	4.28962810930041	9.96888720134234	6.99696605139341
N	3.14954332159793	11.82383462424873	8.10825324886255

N	5.55446995510980	12.62587640045521	9.46103093932948
N	7.02984026827140	10.90903036177338	8.49936713699373
N	6.50802383869460	8.98911848398000	9.94342406209335
C	0.46135581320531	10.43861820476658	11.36533558244432
C	-0.70512152603634	10.22330161637725	12.10583573067749
C	-0.64838354242555	9.61503674374050	13.37263606199384
C	0.59740278270618	9.21537035530796	13.88966342840526
C	1.76910931225798	9.41275419402506	13.15086526436189
C	1.72846130217808	10.03728132993038	11.87145509802184
C	0.92943475474158	8.53525933616315	8.85412309896724
C	-0.35984564744085	8.12274838541615	8.47745015412759
C	-0.92590656349196	8.57533775529674	7.27411122097961
C	-0.17945775346261	9.44302443828930	6.45994885543678
C	1.10577685986015	9.85534331973058	6.85470196093268
C	5.05080399188427	6.31807941361321	7.19090802889670
C	3.23209861433551	13.45584964402688	6.17885461178940
C	6.40111300300893	14.55163173314386	8.05205696025392
C	8.25802671625610	7.42829530668072	8.98512026927502
C	6.12206187668083	8.00452057184555	10.95857217394397
C	5.27278362349291	6.84435558931380	10.41748344814340
C	3.94121266810349	7.25598143029845	9.77275583426086
C	4.65178480649222	7.73285202550267	7.50399917650197
C	4.82305112479473	8.78580856816270	6.53523499694029
C	5.43374444323183	8.74509766817580	5.27115447762008
C	5.46036872669309	9.90828999297481	4.48104887513034
C	4.84217825551014	11.08426140358020	4.93825403753568
C	4.23929823602822	11.09787604909534	6.20796804578772
C	3.52802707528319	12.15741825880901	6.87490524128938
C	2.49260837175809	12.80669079476923	8.96969118984364
C	3.41033175463712	13.95277075374540	9.42091371185652
C	4.65863232554215	13.51810693046668	10.20422749225362
C	6.31934400098777	13.11142321746368	8.46940696161085
C	7.13713929089657	12.10341481936200	7.83607322181478
C	7.97464754748727	12.19366413665585	6.70617693672794
C	8.68405584243689	11.04971323599012	6.29590579356648
C	8.57979275833490	9.84140516786234	7.01009089475224
C	7.73398643600363	9.79059205824247	8.13586244825366
C	7.46679826674243	8.70291032522302	9.04828074095458
H	9.32593420674670	11.10031505505883	5.40894185499900
H	5.94991948600701	9.89268709328426	3.50126404687409
H	0.40785389096226	10.89643910170562	10.37430639173727
H	-1.67138045974239	10.53226902363099	11.68840628204616
H	-1.56586141132594	9.45011731415743	13.94974007777060
H	0.65423065439935	8.73633062206099	14.87510272192961

H	2.73725095185017	9.08877415984749	13.54931451435153
H	1.33312306215250	8.16379051173587	9.80032030670220
H	-0.92392970491401	7.44326370991114	9.13102048109900
H	-1.93079704173100	8.25287159868718	6.97327163103306
H	-0.60056772492869	9.80338664342559	5.51094127639149
H	1.65906736455913	10.53508334429732	6.19449169996253
H	4.53086684426998	5.58895948988102	7.83035353470427
H	4.80546250485090	6.08221074541366	6.14101788393011
H	6.13802547003364	6.15198551895838	7.30722086906376
H	2.36646700093356	13.97264164813583	6.62051593358519
H	4.08961161087511	14.15399169392540	6.20828960764368
H	3.00911650162603	13.27145716030875	5.11385134993021
H	6.15305932713179	15.23765739050120	8.87729465711361
H	7.41927936737143	14.79323104375191	7.70265592663003
H	5.71254587543884	14.78342802763444	7.21693378330857
H	8.26824677382125	6.89389759300055	9.94745127616743
H	7.85664190370423	6.73073169766130	8.22510170930112
H	9.30356549804860	7.63883460785651	8.70101196724872
H	7.02066525467972	7.58329652420530	11.45002833018665
H	5.54914292364931	8.54481217012450	11.72756057386995
H	5.04082394511642	6.17325169063404	11.26634687882728
H	5.86878569897914	6.24855815877328	9.70754922841118
H	3.32948299772330	7.77943593036047	10.52098609524040
H	3.38646408402212	6.34703524454963	9.47055957872223
H	5.88750020668166	7.81501594805310	4.91532584841900
H	4.83090029349696	11.98863075558362	4.32205554981041
H	1.60769375399078	13.23027606003784	8.45632694949481
H	2.13205980171064	12.26514793221035	9.85399526371348
H	2.81810537590164	14.61970974948924	10.07623758447835
H	3.70999524581436	14.56181909002000	8.55289217298727
H	4.34666828004100	12.97784104626682	11.11047768166167
H	5.20338214448848	14.42579548909500	10.52969138171951
H	8.05570674655478	13.13332928994528	6.15134403811792
H	9.13132129422977	8.95173087094749	6.69119421498347

[(^{3}PDI$_2$')Fe$_2$(μ-CCPh)(Ph)(ZnCl$_2$)], (**B**)

G = -7027.21944601 E_h, Singlet

This species has one imaginary frequency (-24.81 cm^{-1}) from an imine methyl rotation_.

Fe	3.61135153950768	10.01014689836580	8.61385762183386
Fe	5.83844950877134	10.78261450244879	10.07407025546167
C	2.90206862168775	10.21748513413560	10.97533564948613
C	4.05822400532579	10.38067907664012	10.53117873754864
C	1.79563328254598	9.45186286764450	8.07697689500457
N	4.17121906840094	8.14874492378779	8.64539239834798

N	4.31189941531694	9.92285348314717	6.95059989581646
N	3.28578046061763	11.83467567973380	8.10107325567195
N	5.58064848640128	12.64759153421157	9.65617482166622
N	6.91507491967857	10.90447595585425	8.59339021897724
N	6.57303525020191	9.00022535820506	10.08082736061175
C	0.42816096228488	10.37753320473987	11.18311396715813
C	-0.73031395485005	10.19345847258416	11.94540348871204
C	-0.65562888563751	9.67620329720322	13.25077398800141
C	0.60074735561919	9.34070393843895	13.78798659503271
C	1.76493634498812	9.51147993114918	13.03118140851387
C	1.70440916538925	10.03911933348496	11.70954375584048
C	1.09236651675724	8.45906188901054	8.78549459388871
C	-0.20131451030135	8.05951114820488	8.41364439030310
C	-0.83001737115538	8.63604540861171	7.29828456510976
C	-0.13632087233404	9.61085055424598	6.56474369861207
C	1.15850990945875	10.00322747499920	6.94980649006777
C	5.04470463225356	6.27705267471712	7.18913621692545
C	3.41262815670175	13.44827435618471	6.15643531131005
C	6.28644873622072	14.55506770962696	8.15369632035871
C	8.20250009754788	7.42353887427566	8.95976188156993
C	6.19022383270645	7.98397532301701	11.06695773779783
C	5.35264521261673	6.84506524000784	10.46797556276066
C	4.02965118140355	7.26180708761353	9.80710587969238
C	4.67281260315020	7.69662811813503	7.50810238403399
C	4.81188535997219	8.73220023760166	6.49879769763428
C	5.37198692501366	8.65362758358759	5.21378553825505
C	5.40736376866183	9.80965306127786	4.41332306504665
C	4.86939052562832	11.01684010137355	4.89012200243898
C	4.31427500298139	11.05552142440098	6.17999670203554
C	3.67445994278689	12.15640311488264	6.87590872349579
C	2.57360425485470	12.79587127112104	8.95326071333904
C	3.43064724315628	13.93025776401025	9.54151047849790
C	4.64295822188883	13.50825870080214	10.38658675550676
C	6.23155554888416	13.11864016660552	8.59047122592034
C	6.97575164955643	12.09637230904506	7.90133849679650
C	7.71125078042582	12.17157959904160	6.70738279657045
C	8.37353213011683	11.02726874589460	6.23353168230207
C	8.32551320383490	9.82849349416265	6.96642721417817
C	7.58879986025684	9.78101271118188	8.15957199174500
C	7.41641043611860	8.69669796874170	9.09238669378670
H	8.93186107814412	11.06959096001539	5.29251079453923
H	5.85135612576252	9.76741599517171	3.41307691197035
H	0.36088696187202	10.76531994830917	10.16354909530396
H	-1.70454248098120	10.45468703589274	11.51394699700777

H	-1.56692786232719	9.53359801655490	13.84350093160079
H	0.67199236225954	8.93586962202075	14.80531556803677
H	2.74370353282617	9.24578137088114	13.44637523057422
H	1.53373525508610	8.00181670593723	9.67363580335643
H	-0.72231155170037	7.29658273609369	9.00766538019860
H	-1.84138277012731	8.32840345793319	7.00432708269600
H	-0.60299395013144	10.07202007258300	5.68340239352161
H	1.66465160658590	10.76191411089513	6.34391267786923
H	4.70423202557296	5.56512348812478	7.95349011558400
H	4.59408005456467	5.98455840760875	6.22379842916388
H	6.13747775221482	6.15814056682113	7.07243773997216
H	2.96947126484919	14.22033235143058	6.79838202736997
H	4.34226685474380	13.85448393020462	5.71980410439212
H	2.72128487305088	13.26968398785552	5.31056835723417
H	6.06481591416179	15.24338577085501	8.98355956110336
H	7.29583880298445	14.79316920027312	7.77711875257415
H	5.57718902466884	14.77632811126652	7.33659597035713
H	8.25256260086137	6.86644745166066	9.90671923854882
H	7.77999385692290	6.74831516675752	8.19392486602030
H	9.23583304226496	7.65243994172374	8.64618470473841
H	7.09246407350756	7.55452329607591	11.54313175055893
H	5.62126088944691	8.50158988572658	11.85148700906560
H	5.09873885738981	6.15483912669349	11.29422394148009
H	5.96123712046074	6.26196325668719	9.75880422110599
H	3.42068481483727	7.79970461792482	10.54624032324702
H	3.47367386485457	6.35151721154210	9.51641687979253
H	5.78155388595302	7.70666080203723	4.85003302461916
H	4.88610715659728	11.92041351365452	4.27372472707221
H	1.73650598688281	13.24267152614227	8.38556109967312
H	2.14014198083751	12.21553845247900	9.77685329844964
H	2.76738494570040	14.52779351928031	10.19468130881844
H	3.75646984620325	14.60993429723398	8.73861407152715
H	4.31594720363399	12.94448959077114	11.27046131112782
H	5.16063618241561	14.41816942496751	10.74666903842556
H	7.74880800714603	13.11219969446726	6.14989113370028
H	8.84446907150767	8.93355156265439	6.61052186759720
Cl	6.13893656560871	11.16629806684724	12.68736546506801
Zn	7.83643863275049	11.44638548406424	11.12831900675673
Cl	9.94615801472263	12.02586055972300	11.19262066451459

[(^{3}PDI$_2$')Fe_2(μ-CCPh)(4-MePh)(PPh_3)] (**1^{H,Me}'**)
G = -5401.07445852E_h, Singlet
This species has 1 imaginary frequency (-4.87 cm^{-1}), corresponding to a methyl group rotation

Fe	3.55379380812605	10.02717185709812	8.66557391969987

Fe	5.82658703001925	10.76547534140018	9.97352524216045
C	2.92106982250375	10.16991182270623	10.79563013908732
C	4.15791224683378	10.39684643247740	10.63271369318660
C	1.71979633591976	9.42221316171589	8.17976124038073
P	6.89981849948494	11.35675700403449	11.78321676733791
N	4.12182411416842	8.16689746541670	8.64537091284449
N	4.28010640503614	9.97000693491512	6.99619802090065
N	3.17981270269159	11.83129005160599	8.13584624941400
N	5.53368185019148	12.62065827472195	9.49572278529448
N	7.01389050308155	10.90167205533126	8.56352202182365
N	6.52354434333460	8.96584101382994	9.97906923557642
C	0.46296105460405	10.42297199682604	11.15542965223604
C	-0.65417933403002	10.30881594911668	11.98972295812114
C	-0.52972229119304	9.79448186534079	13.29366025709501
C	0.73851648632473	9.38899053842903	13.74958961891936
C	1.86010359672351	9.49730821941446	12.91936379487840
C	1.75589431060479	10.03223823305531	11.60193974640126
C	1.01848766630660	8.40833361340964	8.86379155080292
C	-0.24477307224016	7.96138747380835	8.45269189271445
C	-0.88361038139786	8.50535157291588	7.31892525380090
C	-0.18644891625741	9.50703332860404	6.61961640819966
C	1.08414023083666	9.94501747562814	7.03899884916924
C	4.69385604704119	11.61857482669783	13.52991111470965
C	4.07490101259181	11.89952017479165	14.75974956855306
C	4.84426065807201	12.31589964247070	15.85552237356390
C	6.23872009807831	12.44774629907229	15.72027946787738
C	6.85523280691150	12.16468723717695	14.49544764244055
C	6.08508167202952	11.74921477251645	13.38536647615766
C	7.92233696620879	9.32010600400593	13.46349429018718
C	8.77435972190559	8.25365003190968	13.78605335705480
C	9.86562617241976	7.94372390831993	12.95998618873626
C	10.09318689547671	8.71093998496765	11.80612837189664
C	9.24145763058726	9.77551067979947	11.48283749011930
C	8.14810605221313	10.10784535323814	12.31345586594795
C	8.80789964513009	12.94520642631240	10.41095487349810
C	9.42140690561491	14.14783549688969	10.03658290205162
C	9.06818240128484	15.35036290033936	10.66750067590288
C	8.09287961877305	15.33343553884128	11.67633727236240
C	7.48844177317758	14.12693272652258	12.05986794304178
C	7.84715240793494	12.90622108767369	11.44612384444079
C	5.02109243079699	6.32201889008703	7.16903107092797
C	3.22526741261274	13.45386942646189	6.19451670105766
C	6.37561028126407	14.54461245883715	8.10360699038947
C	8.24705365656397	7.40815491746438	8.99474064365777

C	6.13520143273517	7.95479679907842	10.96637997452167
C	5.29932322011901	6.80465151600372	10.39367734549031
C	3.96909879985257	7.24030086636010	9.76880498598189
C	4.64838028986180	7.73928164611874	7.50440052113658
C	4.80885658819286	8.79368633063558	6.52879843163782
C	5.40847477715116	8.74711577940140	5.25853090167404
C	5.43521963370085	9.91331171664591	4.47200749656252
C	4.83662510614663	11.09436016845394	4.94242503792724
C	4.24320291933197	11.10160770695885	6.21782962039296
C	3.54320072248464	12.16414060297145	6.89777982285096
C	2.49537655122520	12.81125263880330	8.98003247899891
C	3.40024954870400	13.96468726276502	9.43163164769785
C	4.63148013446314	13.51499757020351	10.22765726780805
C	6.28888894654021	13.10238268090108	8.51193945092102
C	7.11755398460862	12.09495758430407	7.89287502615162
C	7.96777874909934	12.19793806340823	6.77959701033006
C	8.70276767891965	11.07088426398503	6.37284619520482
C	8.59583346943041	9.86093393369601	7.08401418079436
C	7.73565412501394	9.79362382797299	8.19127730774177
C	7.46607696639321	8.68830828751646	9.08134340140851
H	9.35944115827351	11.13335734507645	5.49807056688553
H	5.91511034236295	9.89785850162020	3.48709002867786
H	0.35384625433413	10.80533086739323	10.13733449178127
H	-1.63838935757471	10.61999148240715	11.61643849138435
H	-1.40900420502820	9.70407594298165	13.94278836289935
H	0.85227912987821	8.97713977426891	14.76089267144001
H	2.84354690107239	9.17064709309908	13.27521545477058
H	1.44313066102901	7.96820321497743	9.76887953682642
H	-0.75339158538474	7.17813035731719	9.03389399679588
C	-2.25186204690239	8.02790899396555	6.88686003916276
H	-0.64346237531091	9.95621185918348	5.72529623736720
H	1.57495259129584	10.72341887235261	6.44359768623461
H	4.10226367206595	11.28428625531841	12.67359080469209
H	2.98893946941423	11.78305142059273	14.85454304211756
H	4.36327351890683	12.53466506726502	16.81650499099529
H	6.84651393906750	12.76911535679416	16.57469924762283
H	7.94218206203462	12.26317197358585	14.39968677094570
H	7.06546665238101	9.52978745580371	14.10991373061893
H	8.57829402380138	7.65968245084148	14.68691492611950
H	10.53008020889664	7.10793802598951	13.20920447002551
H	10.93321403087671	8.47356537771968	11.14257781452531
H	9.42802905006664	10.32953092041484	10.56211663697139
H	9.06193720804697	12.03898425651612	9.86010336138080
H	10.16203905141725	14.14370984668299	9.22802545099033

H	9.53912707038131	16.29381962747135	10.36700827779327
H	7.79523686099392	16.26586952747364	12.17131953083444
H	6.72257203401074	14.14310760298555	12.83986596739720
H	4.51481654564924	5.59152245183454	7.81763291750519
H	4.74283393485281	6.09694374244553	6.12472936201029
H	6.11020693401763	6.14522847991885	7.25008116488454
H	2.36964191413428	13.97458581809983	6.65059387885134
H	4.08028904438046	14.15622793416689	6.19137121461843
H	2.97544541676025	13.25579713129574	5.13752101104390
H	6.18525934219285	15.21801738875152	8.95404167174679
H	7.38478270095171	14.77051292441249	7.72343443843110
H	5.65656498626822	14.80376578750348	7.30467154333724
H	8.31500897198366	6.90348645135214	9.97044791530393
H	7.80408286864887	6.69305843320631	8.27655553323376
H	9.27552276230525	7.61296295195872	8.65379906694210
H	7.03577623079334	7.53802176759807	11.45190803485619
H	5.55518934222274	8.47477045290353	11.74188808916236
H	5.06988276503953	6.11406322273534	11.22745010065197
H	5.89545924800973	6.22629182606996	9.67007194248720
H	3.37524685856049	7.75995473812054	10.53325352997907
H	3.39903924483105	6.34597374369616	9.45320467446525
H	5.85496070024564	7.81583576269226	4.89635307248767
H	4.83731352151937	12.00449983543510	4.33444812097218
H	1.61064504837875	13.21786414325706	8.45311088267053
H	2.13515906584568	12.27048085276950	9.86418773109847
H	2.80463073367946	14.62895100381609	10.08632773120088
H	3.70691982801123	14.57621748048221	8.56814076064027
H	4.29856090433197	12.97188698944950	11.12109485832331
H	5.18828973397248	14.40662044019499	10.57092711593193
H	8.04362835977123	13.14276128050029	6.23250241441541
H	9.16244353436798	8.97755297352544	6.77322449249525
H	-2.59868349503247	8.55654153816249	5.98151249194036
H	-3.00742908518228	8.18789130524509	7.68060806664877
H	-2.25368720425487	6.94289196591942	6.66507014455429

$[(^3PDI_2')Fe_2(\mu\text{-}CCPh)(Ph)(Cl)]^-$, (**C**)

G = -4826.11667617 E_h, Singlet

This structure has two imaginary frequencies (-20.0 and -5.35 cm^{-1}) from imine methyl rotations.

Fe	3.70578121633860	9.98378403760976	8.51685916206062
Fe	6.20035398736371	10.74883252468473	9.68528635683711
C	3.33644853436330	10.19446372305262	10.69228111055286
C	4.55179769074611	10.38143653331304	10.36759101677317
C	1.84679687050124	9.38104875628873	8.22400956027027
Cl	7.03917253135347	11.18256084713382	12.14068550790444

N	4.34454360297361	8.14897983965996	8.47272957620577
N	4.26091616580129	9.92532879454186	6.78508149603462
N	3.33914446576366	11.80509820791536	8.05151735715017
N	5.91212601685513	12.63733329932411	9.27996878526923
N	7.29531099179952	10.93406516115081	8.17796294794128
N	6.97547364448008	8.96538882608823	9.60212486482598
C	0.93998116154800	10.32598978040228	11.42620500749079
C	0.00524258522595	10.41280375927184	12.46613732677561
C	0.41778389208580	10.43676962026544	13.81074529182246
C	1.79777849376954	10.38551020815349	14.09989513933761
C	2.73928238231398	10.29912497060997	13.07166726202280
C	2.33610542152770	10.25726013422887	11.69934134504817
C	1.26344148282296	8.29891240357250	8.91767853074279
C	-0.03834767834108	7.85146328139097	8.65315670135856
C	-0.83901706605839	8.46313415137146	7.66490805819345
C	-0.26309898864344	9.53350018246440	6.95647959907764
C	1.04693488765316	9.97183472180857	7.22759866967111
C	5.13503925832551	6.30354568026098	6.93572072934818
C	3.25483716222624	13.45796586619666	6.13636146625820
C	6.53348681909237	14.56833568579562	7.76511841825340
C	8.53646560994705	7.41368620316692	8.35277704073319
C	6.68654705094246	7.94145704097232	10.60802874573230
C	5.76763557455959	6.81886174440191	10.10430721641060
C	4.37007541777209	7.25125839714911	9.63192754704619
C	4.74800915151437	7.71424867614956	7.28350840348896
C	4.76082041714442	8.75235381116816	6.27728469532388
C	5.22330795697237	8.70336359219180	4.95083126932013
C	5.15939661940013	9.86634141163050	4.16094164776472
C	4.61892220865571	11.05125772022670	4.68966210705098
C	4.16125677927679	11.06303711156935	6.02027870718708
C	3.57883768015555	12.14053776010231	6.78300336717768
C	2.78447746787779	12.79093138994524	8.98280809401650
C	3.74453131172826	13.92237873688374	9.38578726801678
C	5.04400939309372	13.49688097915729	10.09025289201422
C	6.53290134906822	13.12854204714147	8.19998800798614
C	7.27654945097519	12.12656327231777	7.47561247940069
C	7.90856398893435	12.21652761331743	6.22386782195805
C	8.54471125017360	11.08038653081358	5.69013349449021
C	8.55734892763648	9.87146498635976	6.41216402539953
C	7.92307205860294	9.81255660115315	7.66353621327232
C	7.78667070040707	8.69849839012287	8.57172554969257
C	-2.24711929990900	7.98361106028493	7.39141560611310
H	9.02183201335550	11.13332706010994	4.70476212284314
H	5.53361838860721	9.84849502494590	3.13098550615095

H	0.61037792080555	10.30993083157983	10.38464372598284
H	-1.06457685944782	10.46327372710246	12.22206787308583
H	-0.31935866340325	10.50016802805421	14.62064958846181
H	2.13945705524600	10.41708028391705	15.14342572949977
H	3.81420998775573	10.27963420357172	13.28979832027933
H	1.82003578880551	7.80781011377156	9.72081883696728
H	-0.44961142019963	7.01358001998811	9.23580252050571
H	-0.84987608547552	10.03656689107156	6.17304421799937
H	1.44007508277357	10.80621323072716	6.63428523545506
H	4.71868065035761	5.57129456345010	7.64432542533063
H	4.76210303964595	6.04890718652533	5.92826525270259
H	6.23194900510115	6.16126751925346	6.91222126659465
H	2.48049018174010	14.01295232415994	6.68794528511687
H	4.14076797659952	14.11590286450068	6.05479141416399
H	2.88573926325854	13.29499883149702	5.10853154222662
H	6.34821641089316	15.25360972165569	8.60750779996971
H	7.51020548409176	14.83197912970240	7.32394622217478
H	5.76795595536771	14.78122331825562	6.99466707109771
H	8.69616615929637	6.86197159517545	9.29257793062667
H	8.00868635908283	6.73291788946826	7.65762786421207
H	9.52551866490973	7.61844705609037	7.90778253719283
H	7.62889581612752	7.48559801265255	10.97268880217817
H	6.22944996281066	8.46557966768601	11.45981351490281
H	5.61966573627602	6.11149376732173	10.94345496014825
H	6.27909958953619	6.24773148237262	9.31287034192295
H	3.86841850842623	7.78542521765287	10.45164811928005
H	3.77553565429345	6.34327192652715	9.41095835296925
H	5.64232990320976	7.77500897509857	4.54975351546578
H	4.56788946312543	11.96249617046008	4.08510482824340
H	1.87184664158758	13.24114092915945	8.54493063835443
H	2.47789902778546	12.23846182290895	9.87976802721002
H	3.19288877028105	14.58279332964111	10.08293826623850
H	3.98507646481925	14.54209041394116	8.50687218821582
H	4.81737956458062	12.93513177003795	11.00803894045672
H	5.59160703350188	14.41193616807407	10.39351917111191
H	7.88514687316759	13.15798312606283	5.66479116076730
H	9.03867896130343	8.97879722418618	5.99897120180068
H	-2.72127953063679	8.56131888105172	6.57820006748153
H	-2.89073376385444	8.07593483247753	8.28830457338826
H	-2.26561063192686	6.91506711032819	7.10030891509898

[(^{3}PDI$_2$')Fe$_2$(μ-CCPh)(Ph)(4-MePh)]$^{-}$, (**D**)
G = -4597.253277 E_h, Singlet

Fe	6.20838897003157	10.72272629517311	9.79278689092099

Fe	3.66093820734863	9.99839886938983	8.56043177697982
C	1.80800516217255	9.39892581671469	8.28117520743771
C	4.56588422790600	10.35233145078774	10.46046696590713
C	3.31674063871184	10.16305767499431	10.68200746084138
C	7.04406636060222	11.18251520086807	11.52271167733960
N	6.97748962958434	8.97566395366061	9.70798232438461
N	7.33989281008557	10.96777878337791	8.28039138991647
N	5.88674242044024	12.85988498874756	9.22281997139585
N	3.32458243013263	11.82681747015472	8.09666940881475
N	4.26357373773967	9.95489719941997	6.83574884339980
N	4.32874948004530	8.17054707861407	8.51257139595769
C	8.45805768407888	11.15890581208800	11.61956205302119
C	9.13288612230132	11.51473288632217	12.79985715439321
C	8.41114704261403	11.90889664063164	13.94080474243967
C	7.00693620514669	11.93339772190911	13.87382195867073
C	6.34210989696089	11.57282277765950	12.68858403522821
C	-2.30247758959745	8.03927874860242	7.45342240410048
C	7.83783884102474	8.72170652348609	8.70493638550946
C	8.00592450539537	9.83992009437411	7.80982220537023
C	8.68533666779037	9.87640040497427	6.58177500651051
C	8.67770336122296	11.05814385985038	5.82068567566526
C	7.99494582544857	12.19215133833639	6.30017274269859
C	7.32595321528121	12.13315811703127	7.53217671938466
C	6.54942012051616	13.20463419933731	8.13872792282033
C	5.02035061090568	13.76166412846515	9.96106666201893
C	3.67043194685051	14.05179202484631	9.28115392988960
C	2.77957002601654	12.82664174942978	9.01708789115099
C	3.55422792036521	12.15886274381065	6.81799029663787
C	4.15395443173357	11.09168003168891	6.06489083264437
C	4.62562521747777	11.08469275425094	4.73779007020320
C	5.19136481734617	9.90833966963110	4.21543370518852
C	5.26191952020304	8.74550283834048	5.00629243048825
C	4.78158184862572	8.78638693006266	6.32733714118023
C	4.76499674642154	7.74691284369162	7.32477253588542
C	4.35039496434159	7.25788463208311	9.65894798331465
C	5.74367305006415	6.81623227819534	10.13871851789240
C	6.65048410127147	7.93039412401257	10.68277620495608
C	8.58263071006496	7.43028493734917	8.51429669247934
C	6.56598973834984	14.58609590507963	7.53134946430216
C	3.17827106439276	13.45904823443536	6.16088270477999
C	5.17928392139157	6.34246035752050	6.97995584679918
C	1.01114714695984	9.99512158865636	7.28360013837190
C	-0.30409528301870	9.57080461953582	7.01613739449142
C	-0.89027426785992	8.50706238125282	7.72707545777749

C	-0.09327474076942	7.88789800305882	8.71374969661602
C	1.21269817427407	8.32319258859409	8.97626455659681
C	2.28412663239498	10.12639559623997	11.66202865777927
C	2.59107527736573	9.83042531772322	13.02671231363346
C	1.59827454501543	9.81301667664063	14.01123825341263
C	0.25461192462984	10.08480718778584	13.68220266582031
C	-0.06850731287353	10.37881628893800	12.34369822148628
C	0.91901938349031	10.39773853492938	11.35223194543028
H	-2.32618553659398	6.98699198568380	7.10684808394048
H	-2.93181485793688	8.08324407095537	8.36399153486469
H	-2.79059994831844	8.65673909880848	6.67833444557840
H	5.24573473433427	11.58410137344080	12.66787826743368
H	9.04733506273416	10.85375491276763	10.74389606901051
H	10.23189537761659	11.48465743683142	12.83029763738852
H	8.93338904608687	12.18747181653464	14.86539947901485
H	6.42180253833325	12.23032898763781	14.75629511211570
H	9.19262967275242	8.97859199900685	6.21250795125301
H	7.96766858031353	13.11247740986231	5.70795837660832
H	5.52755198141558	14.73073268622590	10.15088908179776
H	4.83889433654183	13.29418819202965	10.94202823606227
H	3.84159979660111	14.61859948362365	8.35192652135623
H	3.10531806344806	14.72848237825622	9.95207720027933
H	2.59586661395709	12.30736180829158	9.96740660817847
H	1.79663711223425	13.17560803115423	8.63924182544351
H	4.56666072991536	11.99464217487839	4.13129414132556
H	5.69819390616552	7.82305133051268	4.60907165665052
H	3.75762655374507	6.35167079751620	9.42301237256072
H	3.84251242548652	7.77818888023802	10.48385634328516
H	6.26876936679189	6.26105865442567	9.34575788901702
H	5.58771984337497	6.09216714731282	10.96272707327531
H	6.15420519575211	8.43289953741143	11.52545748709053
H	7.58064971229452	7.46993183652741	11.07143365918111
H	9.54115665888413	7.61027848579495	7.99925744814211
H	8.01975862635472	6.69748435166805	7.90339688214147
H	8.80735902669624	6.94013769969054	9.47617739788178
H	5.98532949305275	14.62259485069241	6.59057199637853
H	7.59755569994950	14.89041003938722	7.28089271930052
H	6.14449729966812	15.34082269405862	8.21200093683841
H	2.92063980401817	13.28518685412507	5.10184516425422
H	3.99367991577682	14.20763797372489	6.17220865783377
H	2.30237786224159	13.92314646657723	6.64347099868648
H	6.27619723876699	6.19749105006463	7.02560658766360
H	4.87336806587479	6.10127212195573	5.94702457765064
H	4.71901591200008	5.59728022030348	7.64783549613104

H	1.41364300201704	10.82581955951028	6.69023802679009
H	-0.88813917363921	10.07936452747922	6.23366479058857
H	-0.51281792740705	7.05478692157166	9.29801778604325
H	1.76619725009341	7.82900587930386	9.78014819753576
H	3.63464028434309	9.61605837848675	13.28703520440037
H	1.87099871730884	9.58213984116059	15.05018330354062
H	-0.52397352755087	10.06559428752480	14.45480709265006
H	-1.11001212234092	10.59402326023375	12.06841762050572
H	0.65867347694784	10.61870488902907	10.31386135369828
H	5.57838996805052	9.89651174696280	3.18968352833243
H	9.18819605343110	11.09233699800413	4.85149264996218

PPh_3

G = -1035.16456246 E_h, Singlet

P	-4.08443719833809	1.33304112811152	-0.18498923750382
C	-6.12307760133046	-0.15380649858288	-1.36097599128357
C	-6.77038733672061	-1.30581180640646	-1.82969570744156
C	-6.12372136319318	-2.55170836747564	-1.76428779077136
C	-4.83120254013272	-2.63769867753407	-1.22254321080048
C	-4.18759547943214	-1.48673139985443	-0.74082702379198
C	-4.82826217559330	-0.23272268098636	-0.80317742613405
C	-5.45477337103904	0.36449325825650	2.15041639080542
C	-5.93143116694111	0.50788538175673	3.46314529999280
C	-5.71463561217118	1.70239334270125	4.16849251921679
C	-5.02364545943439	2.75969121331656	3.55246448379852
C	-4.55931529710871	2.62239407015402	2.23668457031265
C	-4.76149430590462	1.41996898552642	1.52419562160535
C	-1.45880307893041	0.84912582271201	-0.97352895370720
C	-0.12306003363890	0.44984570217202	-0.82553941815520
C	0.35231816306569	0.03553640483776	0.43033771299399
C	-0.51735569299340	0.01758306586756	1.53236949572592
C	-1.85870758642173	0.40408310626413	1.38208733324413
C	-2.34395466795166	0.82201447924375	0.12648710686822
H	-6.62768377557250	0.81897923668624	-1.42277171883005
H	-7.77842900946193	-1.23030247451983	-2.25500249815586
H	-6.62502773895349	-3.45229728952888	-2.13853239983595
H	-4.32123622552514	-3.60732698034638	-1.16929020925000
H	-3.18412741898003	-1.56530697388636	-0.30934846413466
H	-5.61870125657687	-0.57519899242400	1.61248402030063
H	-6.47071787737154	-0.32064001730352	3.93832467998360
H	-6.08618145007142	1.81154240484605	5.19437750331610
H	-4.85358699968590	3.69704059665089	4.09580774432626
H	-4.02869504731078	3.45421238399410	1.75573767203026
H	-1.82360763390687	1.18119322664240	-1.95407351753009

H	0.55119618986350	0.46989761311513	-1.69026315816965
H	1.39904500188604	-0.26865777033994	0.54960904546674
H	-0.15213210871971	-0.30442772658415	2.51524264746183
H	-2.53229487650327	0.37487313351785	2.24503219854626

THF

G = -232.05396082 E_h, Singlet

O	-0.84030690016647	-1.25982974094550	0.29386584341957
C	-1.90758579210694	-0.69358425005083	-0.48403008723327
C	-1.37794613924771	-1.93873940592072	1.43926512708850
C	-2.88064429348685	-2.08584002908166	1.17895716702234
H	-1.19277999821077	-1.33597287823361	2.35565416126886
H	-0.85766257798562	-2.90804186391232	1.55963271161343
C	-3.17356854049295	-0.81145245659172	0.37343144942194
H	-3.47069276782298	-2.16729041025817	2.10788010506835
H	-3.07925865498634	-2.98311945066167	0.56334864173380
H	-3.26307791518885	0.05759309634859	1.05176205469606
H	-4.09484114970946	-0.87207835226271	-0.23084372981820
H	-2.01644169243171	-1.26137823387730	-1.43368304110855
H	-1.65390690926332	0.35277723574762	-0.74161995127284

$ZnCl_2(thf)_2$

G = -3165.01928417 E_h, Singlet

This species has one imaginary frequency (-20.13 cm^{-1}) from a THF moiety rotation.

Zn	0.53908324146208	0.92314717970981	0.45776225899752
Cl	0.35333427751583	2.76563181845596	1.69939155108126
O	-1.19619702452866	-0.18610290441199	0.66587175135626
O	1.63243892563688	-0.42969263573376	1.59576027281671
C	-1.74670850132864	-1.07583071628378	-0.35391240368121
C	-1.72906659414006	-0.51268954720787	1.98773791666130
C	-2.44164955183808	-1.84847184709380	1.79387641177634
H	-2.41987562551266	0.29723353122465	2.28341889965187
H	-0.88426040029800	-0.54885130188435	2.69446704546957
C	-2.92358473385183	-1.75751730860171	0.33687259543045
H	-3.26202348681604	-1.98837238242603	2.51665390112581
H	-1.72899859203599	-2.68504418516956	1.91174137709890
H	-3.82775355047982	-1.12637658757816	0.26795210915255
H	-3.14973866547941	-2.74021503113129	-0.10858052133230
H	-0.96133450132467	-1.79511968216940	-0.65004912526878
H	-2.01987867842644	-0.46077698443039	-1.22583685521995
C	2.63761657712703	-0.05177580906355	2.58678978341825
C	1.81613786202784	-1.81445136115541	1.16913765765402
C	3.16491440164544	-2.22408084089274	1.75395524675663
H	1.77489063284075	-1.84014825553696	0.06789671722193

H	0.98103700114270	-2.40946591318740	1.58309731077340
C	3.23885834953901	-1.37937570574774	3.03655250899503
H	3.98101877396229	-1.94717759545457	1.06276513133360
H	3.22129507099044	-3.30867059351130	1.94282887467628
H	4.26602560625921	-1.25887495599335	3.41816169379055
H	2.62104495755533	-1.83142008026683	3.83354218165230
H	2.12689099062240	0.51483242511958	3.38135229520335
H	3.38343928158151	0.59961238319982	2.09528024019309
Cl	1.19015910035155	0.69899541952223	-1.66663303938491

$[ZnPhCl_2]^-$
G = -2932.31602703 E_h, Singlet

Zn	-0.28220964727056	0.28347458199633	-0.35883063091753
Cl	1.23077357291017	-1.21830081350822	-1.20048962101065
Cl	-0.25851608856102	0.25899430517880	1.93085750633590
C	-2.35803982334592	2.34765478427078	-0.88180244538079
C	-3.18809884605832	3.17133388023868	-1.66273937812061
C	-3.12374237766762	3.10392373389453	-3.06417053414695
C	-2.22668703545063	2.21032475086286	-3.67184483091854
C	-1.40334026342721	1.39335409224875	-2.87702011555801
C	-1.45012573329982	1.44324861488065	-1.46910232702951
H	-3.88618502884638	3.86656549844431	-1.17764169124035
H	-2.42477056828289	2.41661674283308	0.21287661495077
H	-3.76890843659836	3.74414258274954	-3.67943628284479
H	-2.16906992549498	2.15046836027498	-4.76690511764013
H	-0.70888018980645	0.70165557633489	-3.37376830427879

$[Zn(4\text{-}MePh)(Ph)_2]^-$
G = -2513.44097824 E_h, Singlet

Zn	-0.18787403666120	1.96884784344003	0.37641846597622
C	-1.51381326490544	4.54875680075413	1.29826193594220
C	-1.63196597327765	5.92527551915797	1.56688118477860
C	-0.55966014821904	6.78805597716188	1.28421440144256
C	0.62048370225568	6.25605646930465	0.73798824743372
C	0.71561585992749	4.87452803636116	0.48599651264841
C	-0.34197818046998	3.97567357649107	0.75360792491511
C	-3.08551311312667	1.47943786776368	-0.40114653615863
C	-1.94188604846940	-0.51556068698492	0.26892293445631
C	-3.10832873115586	-1.27316597863756	0.05372882132464
C	-4.27845510996085	-0.64228651817852	-0.40113901594881
C	-4.26320507403185	0.74381582006504	-0.63077609297094
C	5.61473800614060	-0.84916430083111	0.23219103316822
C	2.78472536236101	1.64035148060046	0.95586759744791
C	4.05009567310316	1.02968984051343	0.93103229697037
C	4.25048936325553	-0.19648419692015	0.26184144986968
C	3.13629108098237	-0.77971962986349	-0.37264589306212

C 1.87341743313663 -0.15845610669189 -0.33342031792886
C 1.64399324533612 1.07387731610437 0.32827393102779
C -1.88817570874529 0.88023262503234 0.05201389948748
H -0.64295243491915 7.86401141830331 1.48757199594625
H 1.46677256048365 6.92079952626070 0.51279214996914
H 1.65578623509027 4.48670849565743 0.06484419975674
H -2.56102997542338 6.32969101172708 1.99365959925358
H -2.37390744503874 3.89955601213974 1.52278445209714
H 2.68562517717174 2.59894913287571 1.48798554893265
H 1.03690292559832 -0.66177409405146 -0.84198980865691
H 3.26206387100262 -1.73604317325813 -0.90162025666389
H 4.90226447523373 1.50927039190988 1.43549337522637
H -3.10752690346812 -2.35725612275882 0.23685345871801
H -1.03922221204659 -1.03928970327692 0.61898222165342
H -5.19298059849469 -1.22491820504709 -0.57484821120472
H -5.17286590899449 1.24919720752688 -0.98561225477915
H -3.10659150030089 2.56494517552056 -0.58262836675858
H 6.36384386590757 -0.19696280634164 -0.25725209914282
H 5.98972302598617 -1.04835602218129 1.25461894069208
H 5.59451438223662 -1.80859401994857 -0.31384738115919

$AlCl_3$
G = -1622.83108452 E_h, Singlet
Cl -0.57612380835037 0.39516158241989 1.23045865138408
Cl -0.57407006212034 3.08099137496920 -1.23010167650186
Cl 2.57954084366620 1.73938795790690 -0.00102299624688
Al 0.47642216820451 1.73773552100402 0.00066602136466

$[AlCl_3]^-$
G = -1854.28744575 E_h, Singlet
Cl -1.67908149874717 -0.40285729959827 -2.99418905023713
Cl 1.22952877540175 -1.46243324253350 -1.24308819745944
Cl -2.03523187233348 -2.08125949705652 0.12308402171840
Al -0.79655968951300 -0.63549216140918 -0.98645119809727
C 0.38112956648700 1.96185963458520 -0.15825861090007
C -0.72490432719151 1.09574625367548 -0.02866632110879
C 0.41156948466844 3.22430163539821 0.45802515599950
C -0.68240865981787 3.65376812244159 1.22720648299599
C -1.79608284129765 2.81075502589027 1.37490042923513
C -1.80835729832770 1.55163667247677 0.75126095567922
H 1.25063046350668 1.63844508497220 -0.74783247899948
H 1.28856427510482 3.87472834459224 0.34068755893630
H -0.66588661278740 4.63876006421560 1.71143853011019
H -2.65412144495368 3.13626253966986 1.97803305298068

H	-2.68419713919920	0.90147355538004	0.88700744384677

$ZnEt_2$

G -1938.46616230 E_h, Singlet

C	-1.66235658299990	2.27697536738268	0.12510776860475
C	-0.14446420577613	2.43906393595949	-0.06772595131614
H	0.29935365798656	3.10354344268593	0.69709577215383
H	0.10107355731865	2.87676397460483	-1.05341454679835
H	0.39330001181116	1.47095244313059	-0.00317529492197
Zn	-2.68224070574529	3.97266599982793	0.01976387862609
H	-1.87672669925635	1.81597030648475	1.10138405220666
H	-2.07255379088012	1.58996763152757	-0.63075958076953
C	-3.70047175431765	5.66944557861914	-0.08420848766825
C	-5.22862964815828	5.49414614014181	-0.04544141239293
H	-3.39843684178070	6.18725356882749	-1.00737686407382
H	-3.36823792319322	6.31152581172895	0.74584478294000
H	-5.59359747735605	4.87406781738979	-0.88553767803653
H	-5.76474258938687	6.46347431324388	-0.10503311975000
H	-5.56297794556574	4.99979662514509	0.88585306019619

$[ZnEt_2Ph]^-$

G -2169.75277861E_h, Singlet

Zn	-3.41783458797549	3.27742261304672	0.24334505315193
C	-7.45362786825901	0.61529659091305	1.32454406422209
C	-7.58496452650437	1.97992331468566	1.01683076017809
C	-6.17369360106438	0.03528187128620	1.31900336101537
C	-5.04972809283942	0.82286829950816	1.00702546593465
C	-6.44252606840544	2.74348653857920	0.71005324116964
C	-5.13690457511905	2.19929664271097	0.69256608285520
C	-4.76982113737122	6.05183943208653	-0.12597049601433
C	-3.42585180383026	5.30560704799314	-0.15111726388830
C	-0.38904550487435	2.99384073773528	-0.12210868604849
C	-1.65293184244839	2.18815140197482	0.21986384768299
H	-6.58159155276087	3.80876264902685	0.47549638944690
H	-8.33825933829690	0.01075992016267	1.56582268103927
H	-8.58097777696723	2.44623174212164	1.01741340806873
H	-6.05666857280462	-1.03182887033932	1.55798896152120
H	-4.06031329473098	0.33888168390044	1.00901880286329
H	-5.27040644729740	5.96066305057600	0.85920592748527
H	-4.70188170682191	7.14537642456791	-0.34052330942288
H	-5.48295613690021	5.63570242938688	-0.86618376195102
H	-2.72446197460553	5.78807185725522	0.56826060543505
H	-2.93793862236669	5.46006013117593	-1.14108923213840
H	-1.75498254734737	1.33930683651295	-0.49489897330058

H	-1.51868223512318	1.69304181850846	1.20924756491899
H	0.55992581656788	2.40545497657616	-0.12986297635751
H	-0.46669627845205	3.46966961317483	-1.12130438662680
H	-0.23098014080148	3.82749694087360	0.59263438025963

References

1. Liu, T.; Gau, M. R.; Tomson, N. C., Mimicking the Constrained Geometry of a Nitrogen-Fixation Intermediate. *J. Am. Chem. Soc.* **2020,** *142* (18), 8142-8146.
2. Liu, T.; Murphy, R. P.; Carroll, P. J.; Gau, M. R.; Tomson, N. C., C–C σ-Bond Oxidative Addition and Hydrofunctionalization by a Macrocycle-Supported Diiron Complex. *J. Am. Chem. Soc.* **2022,** *144* (31), 14037-14041.
3. Chenniappan, V. K.; Rahaim, R. J., Titanium-Promoted Cross-Coupling for the Selective Synthesis of Polysubstituted, Conjugated Amides. *Org. Lett.* **2016,** *18* (19), 5090-5093.
4. Fu, S.; Chen, N.-Y.; Liu, X.; Shao, Z.; Luo, S.-P.; Liu, Q., Ligand-Controlled Cobalt-Catalyzed Transfer Hydrogenation of Alkynes: Stereodivergent Synthesis of Z- and E-Alkenes. *J. Am. Chem. Soc.* **2016,** *138* (27), 8588-8594.
5. Korb, M.; Hosseini Ghazvini, S. M. B.; Moggach, S. A.; Meunier, J.-F.; Bousseksou, A.; Low, P. J., Rip It off: Nitro to Nitroso Reduction by Iron Half-Sandwich Complexes. *Inorg. Chem.* **2021,** *60* (7), 4986-4995.
6. Fulmer, G. R.; Miller, A. J. M.; Sherden, N. H.; Gottlieb, H. E.; Nudelman, A.; Stoltz, B. M.; Bercaw, J. E.; Goldberg, K. I., NMR Chemical Shifts of Trace Impurities: Common Laboratory Solvents, Organics, and Gases in Deuterated Solvents Relevant to the Organometallic Chemist. *Organometallics* **2010,** *29* (9), 2176-2179.
7. Evans, D. F., 400. The determination of the paramagnetic susceptibility of substances in solution by nuclear magnetic resonance. *J. Chem. Soc.* **1959,** (0), 2003-2005.
8. CrysAlisPro 1.171.41.109a: Rigaku Oxford Diffraction, Rigaku Corporation, Oxford, UK. (2019).
9. SCALE3 ABSPACK v1.0.7: an Oxford Diffraction program; Oxford Diffraction Ltd: Abingdon, UK, 2005.
10. SHELXT v2014/4: Sheldrick, G.M., Acta Cryst., A, 71, 3-8 (2015).
11. SHELXL-2018/3: Sheldrick, G.M., Acta Cryst., A, 71, 3-8 (2015).
12. Gutmann, V., Solvent effects on the reactivities of organometallic compounds. *Coord. Chem. Rev.* **1976,** *18* (2), 225-255.
13. Schäfer, A.; Horn, H.; Ahlrichs, R., Fully optimized contracted Gaussian basis sets for atoms Li to Kr. *J. Chem. Phys.* **1992,** *97* (4), 2571-2577.
14. Neese, F., Software update: the ORCA program system, version 4.0. *Wiley Interdiscip. Rev. Comput. Mol. Sci.* **2018,** *8* (1), e1327.
15. Grimme, S., Semiempirical GGA-type density functional constructed with a long-range dispersion correction. *J. Comput. Chem.* **2006,** *27* (15), 1787-1799.
16. Grimme, S.; Antony, J.; Ehrlich, S.; Krieg, H., A consistent and accurate ab initio parametrization of density functional dispersion correction (DFT-D) for the 94 elements H-Pu. *J. Chem. Phys.* **2010,** *132* (15).
17. Grimme, S.; Ehrlich, S.; Goerigk, L., Effect of the damping function in dispersion corrected density functional theory. *J. Comput. Chem.* **2011,** *32* (7), 1456-1465.
18. Weigend, F.; Ahlrichs, R., Balanced basis sets of split valence, triple zeta valence and quadruple zeta valence quality for H to Rn: Design and assessment of accuracy. *Phys. Chem. Chem. Phys.* **2005,** *7* (18), 3297-3305.
19. Weigend, F., Accurate Coulomb-fitting basis sets for H to Rn. *Phys. Chem. Chem. Phys.* **2006,** *8* (9), 1057-1065.
